# Transcriptomic Profile and Probiotic Properties of *Lactiplantibacillus pentosus* Pre-adapted to Edible Oils

**DOI:** 10.3389/fmicb.2021.747043

**Published:** 2021-10-14

**Authors:** Esther Alonso García, Juan José de la Fuente Ordoñez, Leyre Lavilla Lerma, María D. Estudillo-Martínez, Sonia Castillo-Gutiérrez, Nabil Benomar, Charles W. Knapp, Hikmate Abriouel

**Affiliations:** ^1^Área de Microbiología, Departamento de Ciencias de la Salud, Facultad de Ciencias Experimentales, Universidad de Jaén, Jaén, Spain; ^2^Área de Estadística e Investigación Operativa, Departamento de Estadística e Investigación Operativa, Facultad de Ciencias Experimentales, Universidad de Jaén, Jaén, Spain; ^3^Centre for Water, Environment, Sustainability and Public Health, Department of Civil and Environmental Engineering, University of Strathclyde, Glasgow, United Kingdom

**Keywords:** *Lactiplantibacillus pentosus*, probiotics, vegetable edible oils, transcriptomics, metabolic pathways

## Abstract

In this study, we determined whether pre-adapting *Lactiplantibacillus pentosus* strains, isolated from Aloreña green table olives, to vegetable-based edible oils improved their robustness and functionality; this may have great importance on their stress response during fermentation, storage, and digestion. Pre-adapting the strains to the corresponding oils significantly increased their probiotic functionality (e.g., auto-aggregation, co-aggregation with pathogens, and mucin adhesion), although results depended on the strain and the oil used for pre-adaptation. As such, we selected olive-adapted (TO) *L. pentosus* AP2-16, which exhibited improved functionality, and subjected it to transcriptomic profiling with the aim to understand the molecular mechanisms involved in the adaptation and the increased functionality. Global transcriptomic analysis of oil-adapted (olive or almond) and non-adapted (control) *L. pentosus* AP2-16 realized that 3,259 genes were expressed, with 2,779 mapped to the reference database. Comparative transcriptomic analysis showed that 125 genes (olive vs. control) and 108 genes (olive vs. almond) became significantly differentially expressed. TO *L. pentosus* AP2-16 responded by rerouting its metabolic pathways to balance energy production and storage, cell growth and survivability, host interactions (glycoconjugates), and other physiological features. As such, the pre-adaptation of lactobacilli with olive oil switches their transcriptional network to regulate robustness and functionality, possibly representing a novel approach toward the design and manufacture of probiotic products with improved stability and functionality.

## Introduction

*Lactobacillus* genus comprises a phenotypically and genetically diverse group of species able to colonize several nutrient-rich ecosystems associated with humans, animals, plants, soil, and foods (Bernardeau et al., 2006; Duar et al., 2017). Their role in food fermentation (e.g., dairy, meat, and vegetables), their GRAS (Generally Recognized As Safe) or QPS (a Qualified Presumption of Safety) status, and their preservative property make them potential candidates as starter and protective cultures in many artisanal and industrial applications (Bernardeau et al., 2008; Bintsis, 2018). Furthermore, some lactobacilli strains are potential probiotics, which are defined as “live microorganisms that, when administered in adequate amounts, confer a health benefit on the host” (Food and Agricultural Organization of the United Nations and World Health Organization, 2002; Hill et al., 2014). Lactobacilli and bifidobacteria are dominant groups in the global probiotic market^1^ since they exhibit health benefits beyond their basic nutritional value. However, lactobacilli of vegetable origin deserve special attention since their novelty and their singular functionality remain yet under-exploited (Granato et al., 2010; Ranadheera et al., 2010; Abriouel et al., 2012; Pérez Montoro et al., 2016).

Recently, a polyphasic approach based on whole genome sequences reclassified *Lactobacillus* genus into 25 genera, including the emended genus *Lactobacillus* (including host-adapted organisms; e.g., *Lactobacillus delbrueckii*, *Lactobacillus iners*, *Lactobacillus crispatus*, *Lactobacillus jensenii*, *Lactobacillus johnsonii*, *Lactobacillus acidophilus*, *Lactobacillus apis*, and *Lactobacillus bombicola*), *Paralactobacillus*, and 23 novel genera (Zheng et al., 2020). This new reclassification reflects the phylogenetic position of lactobacilli groups into robust clades with shared ecological and metabolic properties, with probiotics largely distributed in the new different genera separated from the original *Lactobacillus* genus; yet, they remained within the family *Lactobacillaceae* and “lactobacilli.” The high heterogeneity of lactobacilli is of great importance for the probiotics industry, since health benefits are inherently specific to the strain and host response. As such, health benefits associated with homeostasis maintenance and disease prevention may be disclosed in the specific cross-talk between the probiotic and the host, which is potentiated by the colonization of the gastrointestinal tract (GIT) and adherence to mammalian cells (Corthésy et al., 2007; Bienenstock et al., 2013; Kemgang et al., 2014; Marchix et al., 2018). Furthermore, specific probiotic features of each strain may be potentiated by several factors before and after consumption; in this sense, several studies aimed to increase the stress response in lactobacilli to stressors, such as extreme temperature, pH, osmotic pressure, oxygen, and starvation, during which multiple physiological and molecular mechanisms become involved (De Angelis and Gobbetti, 2011). Diet affects probiotic functionality; thus, substances in the diet can enhance the activity of probiotics, such as prebiotics, and conversely there are also compounds that inhibit or decrease (i.e., stress) the probiotic activity of some strains (Ranadheera et al., 2010; Markowiak and Śliżewska, 2017). Vegetable edible oils are widely consumed as a component of the diet, and some oils are extensively used in the Mediterranean diet, such as olive oil; however, argan oil is only consumed in a few regions (i.e., Morocco). In this sense, Piekarska-Radzik and Klewicka (2021) recently reported a mutual influence of polyphenols (present in oils) and *Lactobacillus* sp. in foods, by which the adaptions in *Lactobacillus* genome (under the influence of polyphenols) occur and the influence of *Lactobacillus* spp. bacteria to generate antimicrobial properties of polyphenols becomes possible.

This work explored the impact of edible oils (e.g., olive, argan, soy, linen, corn, sunflower, and almond) on probiotic features of *Lactiplantibacillus pentosus* (formerly *Lactobacillus pentosus*) isolated from naturally fermented Aloreña table olives (Abriouel et al., 2012; Pérez Montoro et al., 2016), which with further adaptations may improve food production. Up to now, *L. pentosus* has been currently investigated for its role in food fermentation—as the most commonly isolated bacteria from vegetables—and also for its probiotic benefits as a starter culture for the production of foods of dairy origin (Escobar-Ramírez et al., 2020). Thus, the aim of the present study is the application of oil-adapted *L. pentosus* with added value in foods either as starter or protective culture or as a probiotic adjunct in different foods (dairy and non-dairy) and the eventual use of the prebiotic oils as a microencapsulation-based formulation. Pre-adaptation with the mentioned oils showed, in a previous study by Alonso García et al. (2019), that the corresponding oils significantly increased cell viabilities and also induced gene expression (e.g., *fus*, *rpsL*, *pgm*, *groEL*, *enol*, and *prep*) for moonlighting proteins involved in several stress responses and other functions. Furthermore, increasing robustness and functionality of adapted strains may improve the lactic-acid fermentation food production as starter cultures with greater resistance to acids and resilience to less-optimal environmental conditions. As such, this study examined the impact of oils (before and after pre-adaptation) on probiotic activities such as adhesion, auto-aggregation, and antimicrobial activity. Furthermore, here we aimed to decipher the molecular mechanisms underlying oil effects on *L. pentosus* probiotic activity at transcriptomic level and identify the bacterial effectors involved.

## Materials and Methods

### Bacterial Strains and Growth Conditions

Seven strains of *L. pentosus* (previously *L. pentosus*) isolated by Abriouel et al. (2012) from naturally fermented Aloreña green table olives, developed by four small–medium enterprises from Malaga (Spain), were used in this study. Strains were adapted to different oils: sunflower, olive, linen, soy, corn, almond, and argan according to methods by Alonso García et al. (2019). Strains were routinely cultured at 30°C in Man Rogosa and Sharpe (MRS) broth (Fluka, Madrid, Spain) for 24 h and kept in 20% glycerol at −80°C for long-term storage.

### Effect of Oils on Probiotic Properties

All probiotic properties were studied under the following conditions: (1) strains in the presence of 2% of different vegetable edible oils (sunflower, olive, linen, soy, corn, almond, and argan), (2) strains pre-adapted with oils according to Alonso García et al. (2019) and comparing in all cases with (3) strains never exposed to oils (controls).

#### Auto-Aggregation

The auto-aggregation capacity of lactobacilli was determined as reported by Vizoso Pinto et al. (2007). Overnight cultures of lactobacilli strains were grown in the presence or the absence of 2% oil; strains were harvested, washed, and resuspended in sterile Dulbecco’s phosphate-buffered saline (DPBS). After 2 h at room temperature, 100 μl of the suspension was removed and transferred to 900 μl DPBS, and the OD580 (optical density at 580 nm) was measured at times 0 and 2 h. The percentage of auto-aggregation was calculated as follows:


ODattime 0-ODattime 2hODattime 0×100

#### Co-aggregation

The co-aggregation capacity of lactobacilli (strains grown in the presence or the absence of 2% oil and strains pre-adapted with oils) with pathogenic bacteria (*Listeria innocua* CECT 910 and *Salmonella* Enteritidis UJ3449) was carried out according to Vlková et al. (2008). First, 10 ml of overnight cultures of lactobacilli, grown in the presence of 2% oil, and pathogenic bacteria were each harvested, twice washed with sterile DPBS, and then resuspended in DPBS to OD_600_ = 1.0. In new tubes, 3 ml of each suspension (*L. pentosus* and one pathogenic bacteria) were mixed, and the OD_600 *nm*_ of upper suspension were measured at time = 0 and time = 1 h.

The percentage of co-aggregation was calculated as follows:


1-ODattime 1hODattime 0×100

#### Mucin Adhesion

*In vitro* evaluation of the mucin adhesion was performed according to Pérez Montoro et al. (2018) in 96-well plates. One hundred microliters of a mucin solution (1 mg/ml in sterile DPBS, previously filtered and stored at −20°C) was added. The immobilized mucin remained for 1 h at room temperature and was then incubated overnight at 4°C; after this, the wells were washed twice with 200 μl of sterile DPBS (pH 7.2) before adding 100 μl of overnight bacterial suspension in DPBS. Plates were incubated for 1 h at 37°C, washed five times with 200 μl of citrate buffer to remove non-adhered bacteria, and finally resuspended in 200 μl of 0.5% Tween to harvest the adhered bacteria. Viable counts were determined by plating onto MRS agar, and the percentage of mucin-adhered bacteria was calculated as follows:


$$\%\mathrm{Relative}\text{adhesion}=\frac{\frac{\text{CFU}}{\text{ml}}\mathrm{after}\mathrm{adhesion}}{\frac{\text{CFU}}{\text{ml}}\mathrm{before}\mathrm{adhesion}}\times 100.$$

### Transcriptomic Analysis of Oil Pre-adapted vs. Non-adapted Strains

#### RNA Extraction

RNA were extracted from the non-adapted and the *L. pentosus* AP2-16 adapted with two oils (almond and olive); extractions started with the addition of 500 μl of TESL (25 mM Tris, 10 mM EDTA, 20% sucrose, and 20 mg/ml lysozyme; all from Sigma) and 20 μl mutanolysin (20 U) to cell pellets, followed by incubation at 37°C for 60 min with slight shaking. After this, 300 μl of Tri Reagent was added to the lysates and centrifuged to separate cellular debris, and then the supernatants were transferred into an RNase-free tube before proceeding to RNA purification using Direct-zol RNA Miniprep (Zymo Research, CA, United States) according to the manufacturer’s instructions. RNA quantification and quality assessment were carried out using a NanoDrop 2000 spectrophotometer (Thermo Scientific), and TapeStation. RNA concentrations were adjusted to 2 μg/ml per sample for sequencing.

#### RNA Sequencing and Bioinformatic Analysis

The Illumina TruSeq Stranded Total RNA Kit (Illumina, Inc., San Diego, CA, United States) was used to prepare the RNA samples for the reduction of bacterial rRNA and sequencing according to the manufacturer’s instructions. Sequencing was done on the Illumina NovaSeq platform (2 × 150 bp read lengths) at Lifesequencing S.L. (Valencia, Spain).

Raw sequencing reads were processed with the “reformat.sh” script from the BBTools suite (Bushnell; sourceforge.net/projects/bbmap/) to remove low quality bases (Q20) from both ends and short sequences (40 nucleotides). Filtered reads were then scanned for traces of ribosomal RNA with “sortmerna” v2.1 (Kopylova et al., 2012), parameters and databases by default. Resulting reads were aligned to the protein-coding transcriptome of *L. pentosus* BGM48 (GCA_002850015.1) using “Salmon” v1.1 (Patro et al., 2017), with default parameters. Counts were collapsed with “tximport” v1.12.3 (Soneson et al., 2015) and analyzed with “DESeq2” v1.24.0 (Love et al., 2014) packages on R v3.6. Only genes with 10 or more counts across all samples were kept for further comparisons. *p*_*adj*_ < 0.05 and | log_2_*FC*| > 1.5 (where *FC* represents “fold change”) was requested for considering whether a gene was differentially expressed between the studied groups. Gene ontology (GO) terms were obtained from the *L. pentosus* BGM48 page of Uniprot. Only terms with two or more counts across differentially expressed genes (DEG) of at least one comparison were used to generate the figures. Metabolic pathways images were generated with the “Pathview” v1.24.0 (Luo and Brouwer, 2013) R package, and nodes were colored by adding log_2_FC of its genes for each comparison. Volcano plots were generated with the Enhanced Volcano v1.2 (Blighe et al., 2020) R package.

#### Quantitative Real-Time PCR Validation of the Transcriptome Data

To validate the transcriptome data, four genes were selected for real-time PCR (RT-PCR) experiments with *pheS* gene as internal reference (Table 1). Total RNA was extracted from *L. pentosus* AP2-16 strains pre-adapted with either olive or almond oil and non-adapted strain (control) as described above. RNAs were adjusted to a concentration of 500 ng/ml and frozen at −80°C until required for analysis.

**TABLE 1:** Primers and PCR conditions used in this study.

Gene (product)	Primer	Sequence (5′-3′)	Annealing temperature (°C)	PCR product size (bp)	References
*greA* (Transcription elongation factor GreA)	*greAF*	TAACCGCGATCACCTTAAC	56	212	This study
	*greAR*	ACTGGATATTGGCAAGTTCG			
BB562_01140 (Cysteine synthase)	*cysF*	ATGCTCGTACACCATATCCA	56	200	This study
	*cysR*	TCAATGATGGTGGTCGTGG			
BB562_00810 (PTS beta-glucoside transporter subunit EIIBCA)	*ptsF*	TTATAAATTAACCGCGGCAG	56	200	This study
	*ptsR*	ATCAAGGTGACTTACTCGGT			
BB562_11880 (Transketolase)	*trakF*	ATGAACTTAA GCTGAAAGCG	56	199	This study
	*trakR*	ACCGCATGGCCTTTCGATT			
*pheS* (Phenylalanyl-tRNA synthase alpha subunit)	*pheS-21F*	CAYCCNGCHCGYGAYATGC	60	411	Naser et al., 2005
	*pheS-23R*	GGRTGRACCATVCCNGCHCC			

The expression of selected genes (Table 1) was analyzed by quantitative real-time PCR (qRT-PCR) using SensiFAST^TM^ SYBR & Fluorescein One-Step Kit (BIOLINE). Phenylalanyl-tRNA synthase alpha-subunit (*pheS*) gene was used as a housekeeping gene (Naser et al., 2005), and a no-template control was used as a negative control. Quantitative PCRs (qPCRs) were performed in triplicate 20-μl reactions on a CFX96 TouchTM Real-Time PCR Detection System from BioRad. The qPCR conditions were as follows: pre-denaturation at 95°C for 10 min, denaturation at 95°C for 15 s, annealing at X°C for 30 s, and extension at 72°C for 30 s (Table 1); fluorescence signals were collected during annealing and extension, and the whole process was repeated for 40 cycles. Melting-curve analysis included 95°C for 10 s, 65°C for 5 s, and 95°C for 50 s.

### Statistical Analysis

Except for transcriptome analysis (as described in section “*RNA Sequencing and Bioinformatic Analysis*”), statistical analyses were conducted using Excel 2016 (Microsoft Corporation, Redmond, WA, United States) program to determine averages and standard deviations. All analyses were performed in triplicate. Statistical treatment of data was done by analysis of variances in XLStat by Addinsoft 2020.2.2 (New York, United States) software, using Shapiro–Wilk test and Levene test to check data normality and Tukey honestly significant difference test to determine the significance of differences between strains, where *p* < 0.05 was considered statistically significant.

Principal component analysis (PCA) was used to examine variation and highlight correlations between probiotic properties (e.g., auto-aggregation, co-aggregation, and mucin adhesion capacity); *L. pentosus* strains and oils were appraised by Pearson’s correlation coefficient. This multivariate statistical projection method (PCA) was also applied to reduce the dimensionality and analyze the data. Furthermore, dendrograms were designed following a hierarchical analysis according to Ward’s method and the squared Euclidean distance.

## Results

### Probiotic Profile of Grown or Pre-adapted *L. pentosus* Strains With Edible Oils

**FIGURE 1:**
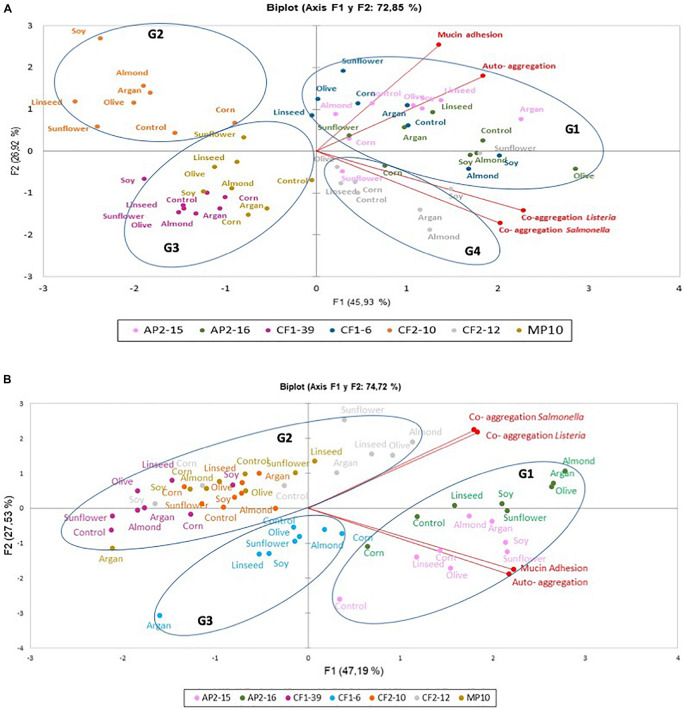
Principal component analysis (PCA) plot based on four probiotic parameters (auto-aggregation, co-aggregation with *Listeria innocua* CECT 910, co-aggregation with *Salmonella* Enteritidis UJ3449, and mucin adhesion) and the position of seven *L. pentosus* strains. **(A,B)** *L. pentosus* strains grown or pre-adapted with 2% edible oils, respectively. Groups were encircled (G1, G2, G3, or G4); each color represents one bacterial species (*L. pentosus* AP2-15, *L. pentosus* AP2-16, *L. pentosus* CF1-6, *L. pentosus* CF1-39, *L. pentosus* CF2-10, *L. pentosus* CF2-12, and *L. pentosus* MP-10), and every dot corresponded to untreated (control) and treated strains with edible oil.

Figure 1A presents the auto-aggregation, co-aggregation of *L. pentosus* with pathogens and mucin adhesion in the absence and the presence of different oils. Results were analyzed by PCA to reduce the number of dimensions to two principal components (F1 and F2), which together explained 72.85% of the total variance. In this context, F1 represented 45.93% variability and comprised co-aggregation variables; however, F2 represented 26.92% variability containing mainly auto-aggregation and mucin adhesion variables (Figure 1A). PCA biplot highlighted the dissimilarity of probiotic *L. pentosus* strains regardless of the oils used; however, PCA highlighted the high correlation between mucin adhesion and auto-aggregation, which did not correlate well with co-aggregation properties (Figure 1A). Furthermore, high correlation of co-aggregation with *Listeria innocua* CECT 910 and *Salmonella* Enteritidis UJ3449 was detected. PCA of strains showed four defined groups regardless of the oils used: G1 (high F1 and F2 components) containing CF1-6, AP2-15, and AP2-16; G2 (high F2) represented by *L. pentosus* CF2-10; G3 (low F1 and F2 components) contained *L. pentosus* CF1-39 and MP-10 strains; and G4, with low mucin adhesion, represented by *L. pentosus* CF2-12. Regarding oils, no differentiated groups were established; however, almond oil showed negative values for auto-aggregation and mucin adhesion variables (Figure 1A).

Oil-adapted *L. pentosus* strains had different induction responses, in respect to probiotic properties, when compared with those grown in the presence of oils (Figure 1). In this sense, PCA biplot showed that the two principal components (F1 and F2) together explained 74.73% of the total variance (Figure 1B). Thus, F1 explained 46.61% of the variability and mainly represented auto-aggregation and mucin adhesion variables; however, F2 represented 28.12% variability with co-aggregation variables (Figure 1B). Pearson correlations indicated highly positive and statistically significant correlation between co-aggregation with *Listeria innocua* CECT 910 and *Salmonella* Enteritidis UJ3449, and (with PCA) between auto-aggregation and mucin adhesion (Figure 1B).

Principal component analysis biplot analysis determined three defined groups of *L. pentosus* strains: G1 represented by *L. pentosus* AP2-15 and AP2-16 strains being *L. pentosus* AP2-16 with high values in both F1 and F2 factors (i.e., olive, argan, and almond adapted strain) and thus represented the best candidate for further study; G2 represented by *L. pentosus* CF1-39, CF2-10, CF2-12, and MP-10 with positive values for co-aggregation variables for *L. pentosus* CF2-12 the best strain with these traits; and G3 represented by *L. pentosus* CF1-6 with negative F1 and F2 variables (Figure 1B).

**FIGURE 2:**
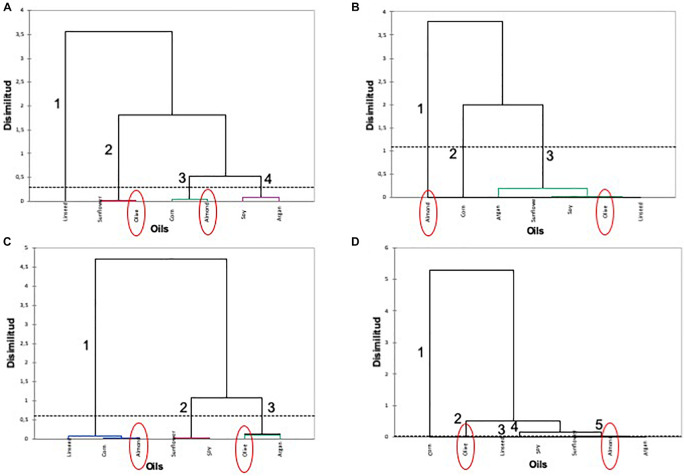
Hierarchical clustering of oils on the basis of each probiotic activity of oil-adapted *L. pentosus* AP2-16. **(A)** Auto-aggregation, **(B)** co-aggregation with *Listeria innocua* CECT 910, **(C)** co-aggregation with *Salmonella* Enteritidis UJ3449, and **(D)** mucin adhesion.

Hierarchical clustering was used to identify similarities using Euclidean distances between the oil treatments used in the adaptation of the selected *L. pentosus* AP2-16 on the basis of its probiotic properties (Figure 2). The oil-adapted *L. pentosus* AP2-16 with almond, argan, and olive oils were characterized by highly exhibited probiotic properties (i.e., auto-aggregation, co-aggregation with *Listeria innocua* CECT 910 and *Salmonella* Enteritidis UJ3449, and mucin adhesion) and high values in F1 and F2 components in PCA analysis (Figure 1B). According to cluster analysis, olive and almond oils were clearly distinguished (Figure 2) belonging to different groups related to auto-aggregation (Figure 2A), co-aggregation with *L. innocua* CECT 910 (Figure 2B), co-aggregation with *S.* Enteritidis UJ3449 (Figure 2C), and mucin adhesion capacity (Figure 2D).

### Global Transcriptomic Analysis of the Oil-Adapted *Lactiplantibacillus pentosus*

**FIGURE 3:**
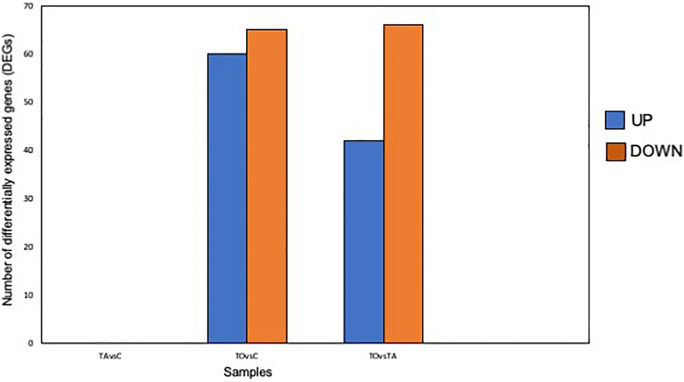
Number of differentially expressed genes (DEGs) between non-adapted and oil-adapted *Lactiplantibacillus pentosus* AP2-16. Samples: TAvsC, almond-adapted strain vs. control; TOvsC, olive-adapted strain vs. control; and TOvsTA, olive-adapted strain vs. almond-adapted strain.

**FIGURE 4:**
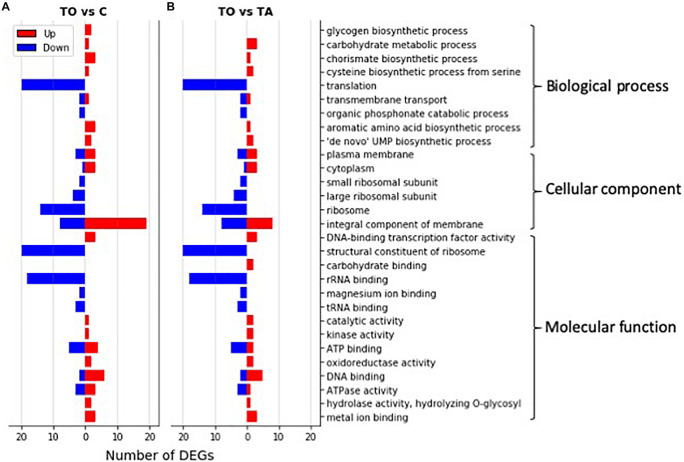
GO classification of significantly differentially expressed genes (DEGs) in oil-adapted *L. pentosus* AP2-16. Significantly up-regulated (red bars) and down-regulated (blue bars) DEGs (log_2_ FoldChange > 1.5) between olive-adapted (TO) and non-adapted (C; **A**) and between olive-adapted (TO) and almond-adapted (TA; **B**) *L. pentosus* AP2-16 were annotated into GO terms in three main GO categories.

Pre-adapted *L. pentosus* AP2-16 with almond and olive oils were selected on the basis of their high values of all probiotic properties and their dissimilarity in hierarchical clustering of oils (Figure 2). They were subjected to transcriptomic analysis, via Illumina Novaseq platform, to compare gene expressions with those of non-adapted control. Raw data could be downloaded from ENA European Nucleotide Archive Database under accession numbers ERR5262947 to ERR5262954, and correlation of gene expression levels between three independent biological replicates was shown by the Pearson correlation coefficient. Clean reads of the three libraries were mapped to *L. pentosus* BGM48 reference genome. Among these reads, about 2,779 genes from 3,259 (85.27%) were successfully mapped (Supplementary Table 1). The degrees of similarity were evaluated between the different samples using a PCA plot (Supplementary Figure 1A) and a heat map (Supplementary Figure 1B); the condition with greatest difference from the control was TO (Olive Treatment), while TA (Almond Treatment) did not seem different from the “control” condition (Supplementary Figure 1). Comparative analysis showed TO *L. pentosus* AP2-16 had 125 and 108 DEGs vs. control (C) and almond-adapted (TA) *L. pentosus* AP2-16, respectively (Figure 3). However, no differential expressed genes (log_2_“fold change” >1.5 at statistically significance level *p* < 0.05) were detected between TA and control (C) of *L. pentosus* AP2-16 (Figure 3). GO terms analysis was used to decipher the biological processes involved in the adaptation of *L. pentosus* AP2-16 to each oil comparing with (non-adapted) controls. While adaptation with almond oil did not produce significant changes in gene expression, the olive-oil adaptation upregulated genes whose putative functions were classified (by GO terms) as biological processes, molecular function, and cellular component (Figure 4).

Figure 4 clearly shows the number of DEGs up-regulated in TO vs. C, and TO vs. TA, were related with carbohydrate metabolism, amino acid biosynthesis, membrane and cytoplasm components, and several other metabolic activities. However, the down-regulated DEGs corresponded to genes involved in translation and ribosome components and activity (Figure 4).

### Analysis of TO vs. C

**FIGURE 5:**
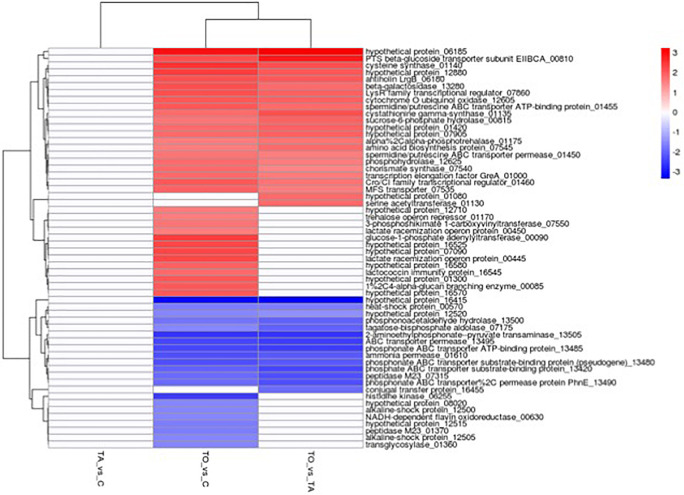
Expression of differentially expressed genes in the oil-adapted (TO or TA) *L. pentosus* AP2-16 vs. control (C). Samples in columns and the encoded genes on rows. Differential expression is indicated with the color key. Samples: TAvsC, almond-adapted strain vs. control; TOvsC, olive-adapted strain vs. control; and TOvsTA, olive-adapted strain vs. almond-adapted strain.

Comparative transcriptomics of TO vs. C indicated that among 2,779 genes, 125 genes were differentially expressed (*p* < 0.05): 60 genes were up-regulated and 65 were down-regulated, involving 18 pathways (Figure 3 and Supplementary Table 1). GO term analysis revealed that adaptation with olive oil up-regulated metabolic pathways: glycogen biosynthetic process, carbohydrate metabolism, amino acid biosynthesis, membrane and cytoplasm components, and several other metabolic activities (e.g., DNA binding transcription factor, kinase activity, catalytic activity, hydrolase activity, oxidoreductase activity, and metal ion binding; Figure 4A). However, the downregulation of translation, ribosome (small and large subunit), organic phosphonate catabolic process, rRNA binding, tRNA binding, and magnesium ion binding metabolic pathways was detected in the adapted strain (Figure 4A). Other genes involved in ATP binding, ATPase activity, and DNA binding were up-regulated and down-regulated (Figures 4A, 5).

### Olive Oil Adaptation Changes Carbohydrate Metabolism in *L. pentosus* AP2-16

Glycolysis/gluconeogenesis, galactose metabolism, pentose phosphate pathway (PPP), and starch and sucrose metabolism were increased in TO *L. pentosus* AP2-16 (Supplementary Table 1 and Supplementary Figure 2). In this sense, the gene encoding 6-phospho-beta-glucosidase (BB562_01895 = BB562_02820) became up-regulated by 1.65-fold (Supplementary Table 1 and Supplementary Figure 2); this is involved in glycolysis/gluconeogenesis and also in starch or glycogen storage (Supplementary Table 1 and Supplementary Figure 2). Similarly, the transketolase coding gene (BB562_03115 = BB562_11880) involved in PPP was up-regulated by 2.04-fold (Supplementary Table 1 and Supplementary Figure 2), and enhanced galactose metabolism was mediated by the over-expression of genes coding for sucrose-6-phosphate hydrolase (BB562_00815) and beta-galactosidase (BB562_13280; Supplementary Table 1 and Supplementary Figure 2). Regarding starch and sucrose metabolism, up-regulated genes coding for 1,4-alpha-glucan branching enzyme (BB562_00085), glucose-1-phosphate adenylyltransferase (BB562_00090), phosphotransferase system (PTS) beta-glucoside transporter subunit EIIBCA (BB562_00810), sucrose-6-phosphate hydrolase (BB562_00815), and alpha, alpha-phosphotrehalase (BB562_01175) were detected (Supplementary Table 1 and Supplementary Figure 2).

### Olive Oil Adaptation Changes Nucleotide and Amino Acid Metabolism in *L. pentosus* AP2-16

Pathway analysis revealed that purine metabolism was down-regulated including the expression of genes coding for adenylate kinase (BB562_12000) and 3′,5′-cyclic-nucleotide phosphodiesterase (BB562_14870) decreasing by 1.91- and 1.67-fold, respectively (Supplementary Table 1 and Supplementary Figure 2). However, pyrimidine metabolism was enhanced, and the corresponding up-regulated genes were those encoding for aspartate carbamoyltransferase (BB562_04230) and dihydroorotase (BB562_04235; Supplementary Table 1 and Supplementary Figure 2).

Several genes involved in amino acid metabolism were up-regulated such as cysteine and methionine; phenylalanine, tyrosine, and tryptophan biosynthesis; and histidine metabolism. However, thiamine metabolism decreased, and alanine, aspartate, and glutamate metabolism was down-regulated and up-regulated (Supplementary Table 1 and Supplementary Figure 2). The up-regulated genes were aspartate carbamoyltransferase (BB562_04230; alanine, aspartate, and glutamate metabolism); cysteine synthase (BB562_01140; cysteine and methionine metabolism); histidinol-phosphatase (BB562_04885; histidine metabolism); and genes coding for chorismate synthase (BB562_07540), 3-phosphoshikimate 1-carboxyvinyltransferase (BB562_07550), and shikimate dehydrogenase (BB562_11875), representing phenylalanine, tyrosine, and tryptophan biosyntheses, respectively. However, the down-regulated genes corresponded to asparagine synthase (BB562_02530; asparagine synthesis and glutamine hydrolysis; Supplementary Table 1 and Supplementary Figure 2) and adenylate kinase thiamine metabolism (BB562_12000; thiamine metabolism; Supplementary Table 1 and Supplementary Figure 2). On the other hand, amino sugar and nucleotide sugar metabolism was increased with up-regulated glucose-1-phosphate adenylyltransferase (BB562_00090; Supplementary Table 1 and Supplementary Figure 2).

### Olive Oil Adaptation Changes Other Metabolic Pathways in *L. pentosus* AP2-16

In metabolic pathways involved in the transport of a variety of substrates, such as ATP-binding cassette (ABC) transporters and PTS, various upregulations and downregulations were observed (Supplementary Table 1 and Supplementary Figure 2). The genes encoding spermidine/putrescine ABC transporter permease (BB562_01450) and spermidine/putrescine ABC transporter ATP-binding protein (BB562_01455) were up-regulated in response to olive adaptation in *L. pentosus* AP2-16 (Supplementary Table 1 and Supplementary Figure 2). However, the genes coding for an iron ABC transporter ATP-binding protein (BB562_08670), glycerol-3-phosphate ABC transporter permease (BB562_10810 and BB562_10815), glycerol-3-phosphate ABC transporter ATP-binding protein (BB562_10820), phosphate ABC transporter substrate-binding protein (BB562_13370 = 13420), phosphonate ABC transporter ATP-binding protein (BB562_13485), phosphonate ABC transporter permease protein PhnE (BB562_13490 gene was 4.29-fold down-regulated; Supplementary Table 1 and Supplementary Figure 2). With respect to PTS, PTS beta-glucoside transporter subunit EIIBCA was up-regulated (BB562_00810 gene), and PTS sugar transporter subunit IIB was down-regulated (BB562_02830 = BB562_16190; Supplementary Table 1 and Supplementary Figure 2).

On the other hand, metabolic pathways involved in regulatory systems such as two-component system were mainly up-regulated, such as genes phosphatase (BB562_00305 = BB562_06980), antiholin LrgB (BB562_06180), and a hypothetical protein (BB562_06185; Supplementary Table 1 and Supplementary Figure 2). However, a phosphate ABC transporter substrate-binding protein was down-regulated (BB562_13370 = BB562_13420). The genes (BB562_11985, BB562_12020, BB562_12025, BB562_12030, BB562_12035, BB562_12040, BB562_12045, BB562_12050, BB562_12055, BB562_12060, BB562_12065, BB562_12070, BB562_12080, BB562_12085, BB562_12090, BB562_12095, and BB562_12100), and the genes encoding 30S and 50S ribosomal proteins involved in nucleic acid and protein synthesis were down-regulated (Supplementary Table 1 and Supplementary Figure 2). With respect to other metabolic pathways, phosphonate and phosphinate metabolism was down-regulated, while sulfur metabolism was up-regulated (Supplementary Table 1 and Supplementary Figure 2). The down-regulated genes and encoding for phosphonoacetaldehyde hydrolase (BB562_13500) and 2-aminoethylphosphonate-pyruvate transaminase (BB562_13505) are involved in phosphonate catabolism (Supplementary Table 1 and Supplementary Figure 2). Nevertheless, a cysteine synthase gene (BB562_01140) involved in sulfur metabolism and cysteine synthesis was up-regulated (Supplementary Table 1 and Supplementary Figure 2).

### Analysis of TO vs. TA

Comparative transcriptomics of TO vs. TA (those pre-adapted with olive and almond oils, respectively) indicated that among 2,779 genes, 108 genes were differentially expressed (*p* < 0.05): 42 genes up-regulated and 66 down-regulated, involving 21 pathways (Figures 3, 5, Supplementary Figure 3, and Supplementary Table 2). GO terms analyses between TO vs. C (Figure 4A) and TO vs. TA (Figure 4B) indicated similar results, although differences in the level of DEGs were detected (Figure 4). Thus, several pathways were similarly regulated; no significant changes were described between control and TA *L. pentosus* AP2-16. However, few changes were recorded, and here, we will highlight these changes with respect to the data analyzed in the previous section.

With respect to changes in carbohydrate metabolism (Supplementary Table 2 and Supplementary Figure 3) produced by olive-oil adaptation vs. almond-oil adaptation of *L. pentosus* AP2-16, additional genes involved in glycolysis/gluconeogenesis were up-regulated in comparison with TO vs. C, such as genes encoding galactose mutarotase (BB562_08780) and phosphoenolpyruvate carboxykinase (BB562_15510; Supplementary Table 2 and Supplementary Figure 3). Furthermore, phosphoenolpyruvate carboxykinase (BB562_15510) was also involved in citrate-cycle metabolism (Supplementary Table 2 and Supplementary Figure 3) and pyruvate metabolism (Supplementary Table 2 and Supplementary Figure 3), and galactose mutarotase was also involved in galactose metabolism (Supplementary Table 2 and Supplementary Figure 3). The up-regulated genes encoding proteins involved in PPP were the same as reported in TA vs. C (Supplementary Table 2 and Supplementary Figure 3). Regarding starch and sucrose metabolism, all the genes up-regulated in the case of TO vs. C were detected, except two genes coding for 1,4-alpha-glucan branching enzyme (BB562_00085) and glucose-1-phosphate adenylyltransferase (BB562_00090; Supplementary Table 2 and Supplementary Figure 3).

Regarding changes in nucleotide metabolism, upregulation and downregulation of genes encoding proteins involved in purine and pyrimidine metabolism were the same as in TO vs. C (Supplementary Table 2 and Supplementary Figure 3). Amino acid metabolism (Supplementary Table 2 and Supplementary Figure 3) exhibited similarity in alanine, aspartate, glutamate, and thiamine metabolism (Supplementary Table 2 and Supplementary Figure 3); however, few changes were detected in other amino acid metabolic pathways. In this manner, additional up-regulated genes included cysteine and methionine metabolism such as serine acetyltransferase (BB562_01130; Supplementary Table 2 and Supplementary Figure 3). On the other hand, we detected the absence of up-regulated genes coding histidinol-phosphatase (BB562_04885) involved in histidine metabolic pathway, glucose-1-phosphate adenylyltransferase (BB562_00090) involved in amino sugar and nucleotide sugar metabolism, and 3-phosphoshikimate 1-carboxyvinyltransferase (BB562_07550) and shikimate dehydrogenase (BB562_11875) implicated in phenylalanine, tyrosine, and tryptophan biosynthesis (Supplementary Table 2 and Supplementary Figure 3).

Concerning other metabolic pathways (Supplementary Figure 3), similar behaviors were observed in the induction of genes coding for PTS, two-component system, and phosphonate and phosphinate metabolism (Supplementary Table 2 and Supplementary Figure 3). On the other hand, ABC transporters, ribosome, and sulfur metabolism exhibited some changes regarding the genes up-regulated or down-regulated in TO vs. TA, when comparing with TO vs. C (Supplementary Table 2 and Supplementary Figure 3). In this case, we observed the absence of down-regulated gene BB562_10815 encoding glycerol-3-phosphate ABC transporter permease (ABC transporters), down-regulation of two additional genes coding for ribosome proteins (BB562_12015 and BB562_11990; Supplementary Table 2 and Supplementary Figure 3), and encoding serine acetyltransferase (BB562_01130) involved in sulfur metabolism (Supplementary Table 2 and Supplementary Figure 3). Other new metabolic pathways were differentially repressed or induced between TO and TA, such as bacterial secretion system through upregulation of a gene encoding a conjugal-transfer protein (BB562_16455; Supplementary Table 2 and Supplementary Figure 3), RNA polymerase via downregulation of DNA-directed RNA polymerase subunit alpha gene (BB562_11980; Supplementary Table 2 and Supplementary Figure 3), and sulfur relay system by the upregulation of a gene coding for molybdopterin biosynthesis protein MoeB (BB562_09880; Supplementary Table 2 and Supplementary Figure 3).

### Analysis of TA vs. C

Comparative transcriptomics of TA vs. C indicated that among 2,779 genes, no genes were differentially expressed [*p* < 0.05 and | log_2_*FC*| > 1.5 (where *FC* represents “fold change”)] (Figures 3, 5 and Supplementary Table 3). Thus, no metabolic pathways were up-regulated or down-regulated according to mapped known genes.

### Quantitative Real-Time PCR Validation of Differential Expression

**FIGURE 6:**
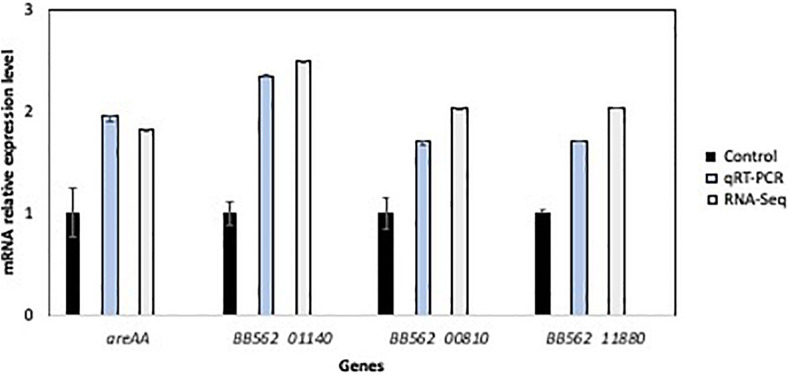
Validation of relative RNA sequence results of four selected genes in the non-adapted cells (control) and olive-adapted *L. pentosus* AP2-16, by comparing with qRT-PCR results. All values were normalized to “control” values.

To verify the RNA-Seq results, qRT-PCR was used to compare the expression activity of non-adapted cells (control) and TO *L. pentosus* AP2-16. In general, gene expressions in the TO bacteria were higher for the following genes: *greA* (1.95- and 1.82-fold higher, as determined by qPCR and RNA-seq, respectively), *BB562_01140* (2.35- and 2.49-fold), *BB562_00810* (1.7- and 2.04-fold), and *BB562_11880* (1.7- and 2.04-fold higher; Figure 6). The qRT-PCR validates the differential expression, and it was consistent with the RNA-Seq; thus, the transcriptomics results were considered representative.

## Discussion

Under environmental, diet, and digestive conditions, probiotic bacteria encounter several stressors including temperature, acid, bile, dietary components, oxidative and osmotic stresses, and antimicrobials, among others. To counteract the detrimental effects of hostile conditions and stress, probiotic responses resultant from adaptation mechanisms induce biochemical and physiological changes to assure survivability or even enhanced probiotic activity. In this sense, several reports focused on the search for new strategies to increase the robustness of beneficial bacteria, taking advantage of the natural adaptations under adverse conditions. Previous studies (e.g., Desmond et al., 2001; Corcoran et al., 2006; Mills et al., 2011; Pérez Montoro et al., 2018; Gaucher et al., 2019) showed that adaptation to different stresses (e.g., salt, low pH, bile, and high temperature) could be strategically used to enhance the technological performance of probiotic lactobacilli. Furthermore, Casado Muñoz et al. (2016) indicated that adaptation to sub-lethal concentrations of antimicrobials could be a good way to achieve desirable robustness of the probiotic *L. pentosus* MP-10 to various environmental and gastrointestinal conditions (e.g., acid and bile stresses), as revealed by proteomic analysis. Similarly, Pérez Montoro et al. (2018) showed that *L. pentosus* strains pre-exposed to acids further displayed enhanced probiotic function, such as auto-aggregation ability via surface proteins. Furthermore, Alonso García et al. (2019) demonstrated that pre-adaptation of probiotic lactobacilli to edible oils increased their survivability and stress resistance. Taking these reports into consideration, the current study had two main goals: first, to evaluate *in vitro* how dietary oils affect probiotic activities, and second, to determine how pre-adaptation with vegetable edible oils increases the functionality of probiotic lactobacilli and which molecular mechanisms are involved.

Here, we analyzed the impact of edible oils (sunflower, olive, linen, soy, corn, almond, and argan) on the probiotic activities of *L. pentosus* strains isolated from Aloreña green table olives (Abriouel et al., 2012; Pérez Montoro et al., 2016), and the results showed that their growth in the presence of some edible oils may decrease probiotic properties in several cases, depending on the strain and also the oil used. However, an increase in the auto-aggregation and mucin adhesion capacities of probiotic bacteria was also observed, which also depended on the strain tested and the oil used. On the other hand, previous adaptations of *L. pentosus* strains with oils improved their response, and thus probiotic activities increased in some cases, which were strain and oil dependent. In fact, a previous study carried out with the same *L. pentosus* strains by Alonso García et al. (2019) showed that the survival and growth of these strains in the presence of vegetable-based edible oils (i.e., sunflower, olive, linseed, soy, corn, almond, and argan) exhibited a slight decrease in viability. However, pre-adaptation increased their viability and resistance to acidic and bile conditions, including the induction of genes (e.g., *fus*, *rpsL*, *pgm*, *groEL*, *enol*, and *prep*) for moonlighting proteins related to several stress responses and functions. As such, this study confirms that pre-adaptation of probiotic *L. pentosus* strains with oils constitutes a new strategy to increase their robustness and functionality. Multivariate analysis of probiotic properties in both exposure cases (i.e., either with oils in growth media or after previous adaptation with oils) suggest that the response of *L. pentosus* strains displayed dissimilarities; however, mucin adhesion and auto-aggregation were highly correlated, suggesting commonality in the molecular mechanisms involved in such activities. On the basis of this analysis, we selected *L. pentosus* AP2-16, with the best probiotic profile, and subjected their results to PCA and hierarchical clustering to determine dissimilarity between oils among the same strain and ascertain the best strategy for determining their molecular responses for probiotic properties. As such, taking into consideration the varied adaptive responses between strain and conditions, it was decided that TO and TA *L. pentosus* AP2-16 were to be analyzed at transcriptomic level against those under control conditions (i.e., without oil adaptation) to explore the underlying bacterium-mediated mechanisms responsible for improving probiotic properties (i.e., auto-aggregation, co-aggregation, and mucin adhesion) under oil adaptation conditions. However, the same strain (*L. pentosus* AP2-16) did not exhibit enhanced acid resistance and bile tolerance after olive and almond pre-adaptation, whereas sunflower-adapted *L. pentosus* AP2-16 showed improved acid resistance and bile tolerance, involving different molecular mechanisms such as the over-expression of genes inducing several moonlighting proteins related with stress response and other functions (Alonso García et al., 2019).

This is the first study to focus on the transcriptomic analysis of the impact of edible oils on probiotics, especially the responses of a potentially probiotic bacteria of vegetable origin. The results showed no changes exhibited between control and TA *L. pentosus* AP2-16; however, the TO strain showed significant differences vs. control (125 significantly DEGs) and also vs. the TA strain (108 significantly DEGs). In this case, the enhanced probiotic properties, shown by pre-adapted almond strain, may rely on changes in structural properties of the cell surface induced by oil adaptation. Among the 125 DEGs in TO *L. pentosus* AP2-16, 60 genes were up-regulated involving the glycogen-biosynthesis process, carbohydrate metabolism, amino acid biosynthesis, membrane and cytoplasm components, and several metabolic activities. However, genes involved in translation, organic phosphonate catabolic process, ribosome (small and large subunit), rRNA binding, tRNA binding, and magnesium-ion binding were down-regulated. These results shed light on the applicability of -omics to decipher the molecular mechanisms behind probiotic activity enhancement. Oil exposure and adaptation of probiotics in different environments (e.g., probiotic preparation, food matrix, or gut) may induce several responses responsible for affecting the host. Thus, depending on the probiotic strain, environment, and stressor, different responses will be obtained, and an in-depth analysis will be necessary to achieve the desirable probiotic effects of strains.

With respect to carbohydrate metabolism, the upregulation of genes coding for proteins participating in glycolysis/gluconeogenesis, galactose metabolism, PPP, and starch and sucrose metabolism were detected. This metabolic shift in carbohydrate was employed by *L. pentosus* to enhance energy production to resist olive oil adaptation. In this sense, glycoside hydrolase enzymes represented by 6-phospho-β-glucosidase and beta-galactosidase (Family 1), alpha, alpha-phosphotrehalase (Family 13) and sucrose-6-phosphate hydrolase (Family 32) were over-produced; these catalyze the hydrolysis of the glycosidic bond between two or more carbohydrates, or between a carbohydrate and a non-carbohydrate moiety of β-glycosides, to yield monosaccharides. Furthermore, the over-expression of genes coding for glycosyltransferases, such as glucose-1-phosphate adenyltransferase and 1,4-alpha-glucan branching enzyme which potentiate the biosynthesis of glycoconjugates by the transfer of sugar moieties from an activated donor to a specific substrate (Lairson et al., 2008), contribute to the biosynthesis of disaccharides, oligosaccharides, and polysaccharides like glycogen (storage function). On the other hand, substrate transport was increased by means of the over-production of PTS-beta-glucoside transporter subunit EIIBCA, involved in the uptake of carbohydrates necessary for growth.

Overall, the over-produced proteins participating in carbohydrate metabolism and transport may contribute a key role in the adaptation of *L. pentosus* AP2-16 to olive oil but also create an advantage in its adaptation during fermentation of plant-based foods and also GIT environments (Corcoran et al., 2005). In food fermentation, the increased carbohydrate metabolism is a desirable trait, and the upregulation of transketolase (enzyme of the PPP) encoding gene may give to the TO strain a competitive advantage in plant-based foods and also in GIT since ribose is abundant in plant materials and also in the diet.

Furthermore, as a probiotic, the specific interaction with the host may be facilitated and enhanced by glycoconjugates (Kay et al., 2010). This was previously reported in *L. pentosus* MP-10 (Abriouel et al., 2017) and also in other probiotics (Kim et al., 2009). On the other hand, transcriptomes of TO *L. pentosus* AP2-16 showed additional differences when compared with TA strain, since other genes were also up-regulated being involved in galactose metabolism, citrate cycle, and pyruvate metabolism. All adaptive mechanisms triggered by TO *L. pentosus* AP2-16 with respect to carbohydrate metabolism showed a coherent picture of changes in gene expression expected to result in energy production, energy storage, and other biological processes to help the bacteria adapt to the new environment by enhancing its capacity to auto-aggregate, co-aggregate to pathogenic bacteria, and also to adhere to mucin.

Transcriptomic profiling provided evidence that *L. pentosus* AP2-16 also responded to olive oil adaptation by regulating nucleotide and amino acid metabolism. Upregulation and downregulation of genes involved in nucleotide metabolism showed that olive oil adaptation of *L. pentosus* AP2-16 induced downregulation of adenylate kinase involved in adenine synthesis and conversion of AMP to ADP (Sanchez et al., 2005), catalyzing the reversible interconversion of ATP and AMP into two ADP molecules, and 3′,5′-cyclic-nucleotide phosphodiesterase involved in the phosphodiester bond degradation in the second messenger molecules cAMP and cGMP. Therefore, both enzymes related with ATP synthesis were regulated to control energy balance in the adapted strain. However, enzymes involved in *de novo* pyrimidine biosynthesis were induced, such as aspartate carbamoyltransferase and dihydroorotase, suggesting that adaptation to olive oil may be advantageous for probiotic lactobacilli in terms of adhesion to the host; Vogel-Scheel et al. (2010) showed that purine and pyrimidine synthesis are necessary for a successful colonization of mouse intestine by *E. coli*. On the other hand, previous adaptation of *L. pentosus* AP2-16 with olive oil induced a reverse transcriptional response of genes involved in purine and pyrimidine metabolism since Piekarska-Radzik and Klewicka (2021) reported that the exposure of *Lactobacillus* to polyphenols (present in olive oil) can cause increased expression of the genes responsible for the biosynthesis of purines and contribute to the reduced transport of pyrimidines. Thus, the adaptive response may highly rely on the strain and the polyphenol used, which in turn depend on the oil composition suggesting by the way that the adaptation strategy used to optimize the functionality of probiotics should be investigated case by case. The genes coding for sugar nucleotides and amino sugars were up-regulated including proteins precursors for extracellular exopolysaccharide (EPS) biosynthesis (Boels et al., 2001). This carbon flux toward polymer production from glucose is supported by the fact that glycosyltransferases (glucose-1-phosphate adenyltransferase and 1,4-alpha-glucan branching enzyme) were up-regulated. The role of EPS in probiotic lactobacilli was largely reported due to their benefits and health potential (Angelin and Kavitha, 2020).

Concerning amino acid biosynthesis, the over-production of some key enzymes (e.g., proteins involved in cysteine, methionine, phenylalanine, tyrosine, tryptophan, and histidine biosynthesis) by the TO *L. pentosus* AP2-16 is an interesting avenue for probiotic bacteria since phenylalanine, methionine, and tryptophan are among the essential amino acids for humans. Furthermore, histidine, cysteine, and tyrosine required by children (Joint WHO/FAO/UNU, 2007) also increased. These data suggest that the adaptation of lactobacilli strain with olive oil is of great biotechnological importance to increase its use as a starter culture and also as a probiotic, supplying valuable human amino acids. The role of these amino acids in the adaptation of *L. pentosus* AP2-16 to olive oil seems to be related with cell growth and survivability, playing a key role in bacterium physiology such as intracellular pH control, generation of metabolic energy or redox power, and stress resistance (Fernández and Zúñiga, 2006). However, nucleic and protein synthesis decreased in the TO *L. pentosus* AP2-16 by means of genes coding for 30S and 50S ribosomal proteins, suggesting that their abundance was affected by olive-oil adaptation controlling primarily at the level of translation, and rRNA transcription as the rate-limiting step in ribosome biosynthesis. This fact is dependent on the levels of ATP available for consumption since ATP promotes the initiation of ribosomal RNA (rRNA) transcription and is consumed by translating ribosomes (Schneider et al., 2002). This change in transcription after adaptation seems to be involved in energy saving.

Regarding ABC transporters and PTS proteins, both pathways (important for the influx of essential nutrients and efflux of toxic molecules, and carbohydrate uptake, respectively) were up-regulated and down-regulated after olive oil adaptation. Thus, to maintain cell growth, metabolism regulation was achieved by upregulation and downregulation of several genes involved in transport, uptake of nutrients, and regulatory systems (two-component system).

## Conclusion

Probiotic lactobacilli retain a broad arsenal of molecular mechanisms to combat the frequent harsh environmental stresses encountered during processing and ingestion, especially *L. pentosus* due to its genetic heterogeneity and plasticity. Thus, understanding the molecular mechanisms involved in the adaptation of potential probiotic lactobacilli is crucial for its use as probiotics improving their viability during technological stresses, storage, and digestion and also their functionality. In the present study, transcriptional changes were reported in the potential probiotic TO *L. pentosus* AP2-16 which exhibited improved functionality, rerouting its metabolic pathways highly connected and linked to energy balance control (energy production and energy storage), cell growth and survivability, interaction with host (glycoconjugates), and other physiological features. Pre-adaptation of some lactobacilli with olive oil may represent a novel approach for manufacturing probiotic products with improved stability and functionality.

## Data Availability Statement

The datasets presented in this study can be found in online repositories. The names of the repository/repositories and accession number(s) can be found in the article/Supplementary Material.

## Author Contributions

HA: supervision, project administration, conceptualization, methodology, writing – original draft, and writing – review and editing. EAG: conceptualization, methodology, and writing. JFO: conceptualization and methodology. LLL, ME-M, and SC-G: methodology, writing – review and editing. NB: supervision, conceptualization, methodology, writing – original draft, and writing – review and editing. CK: conceptualization, writing – original draft, and writing – review and editing. All authors contributed to the article and approved the submitted version.

## Conflict of Interest

The authors declare that the research was conducted in the absence of any commercial or financial relationships that could be construed as a potential conflict of interest.

## Publisher’s Note

All claims expressed in this article are solely those of the authors and do not necessarily represent those of their affiliated organizations, or those of the publisher, the editors and the reviewers. Any product that may be evaluated in this article, or claim that may be made by its manufacturer, is not guaranteed or endorsed by the publisher.

## Footnotes

1
https://www.marketsandmarkets.com/Market-Reports/probiotic-market-advanced-technologies-and-global-market-69.html

## Abbreviations

TO: olive-adapted strain
TA: almond-adapted strain
C: control
DEG: differentially expressed genes.
