# Supplementary material for: Transcriptomic Profile and Probiotic Properties of Lactiplantibacillus pentosus Pre-adapted to Edible Oils
Source: Front Microbiol. 2021 Oct 14;12:747043. doi: 10.3389/fmicb.2021.747043 (PMC8553220; doi:10.3389/fmicb.2021.747043)
Supplement: Supplementary file 1 [file Data_Sheet_1.PDF]

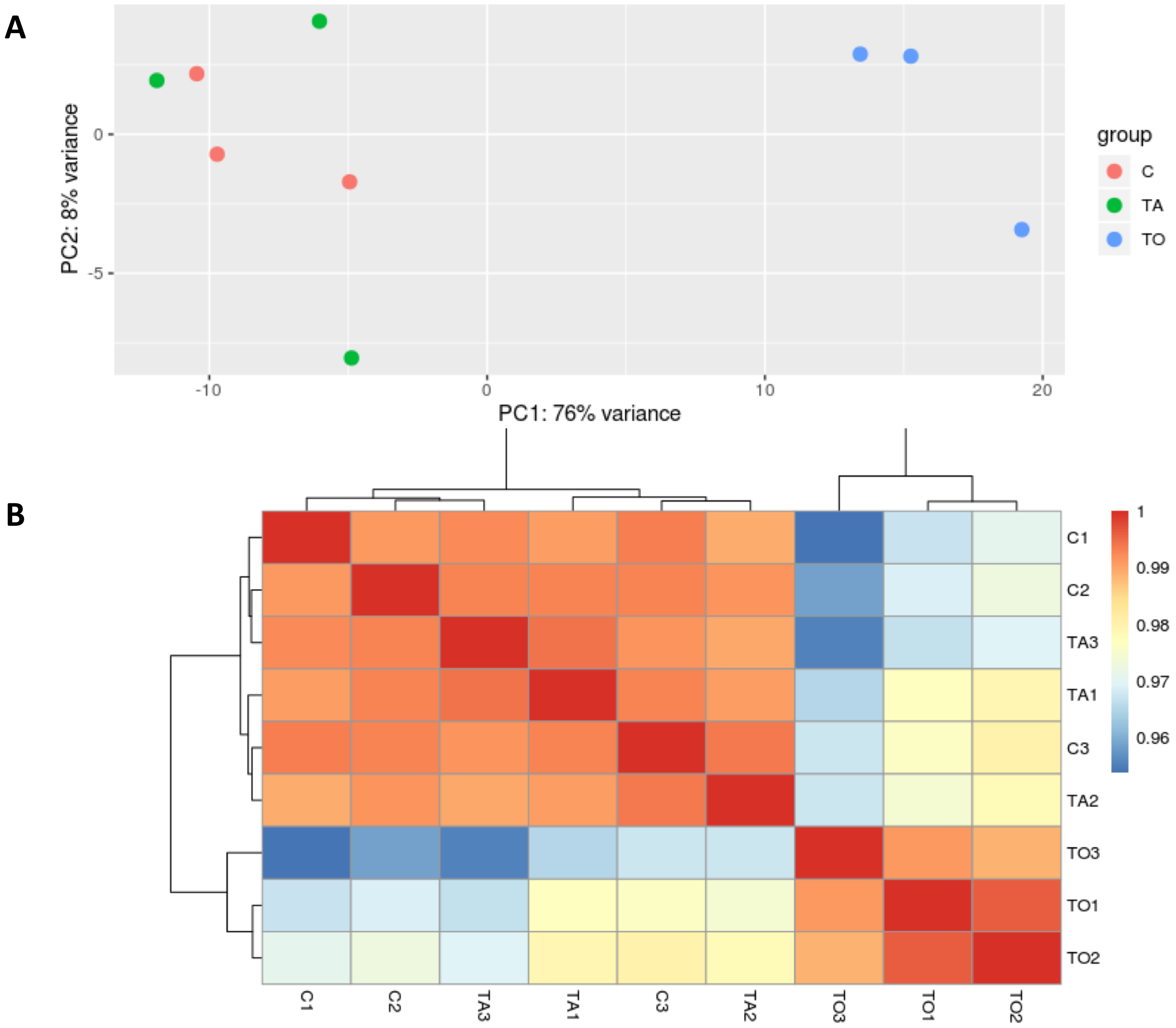
A
PC2: 8% variance
PC1: 76% variance
0
-5
-10
0
10
20
group
C
TA
TO
B
C1
C2
TA3
TA1
C3
TA2
TO3
TO1
TO2
1
0.99
0.98
0.97
0.96

Figure S1

Legend:

Figure S1. Correlation among *Lactiplantibacillus pentosus* AP2-16 RNA-seq samples. (A) Principal component analysis (PCA) of transcript levels of RNA-seq samples. The analysis was performed on R using the expression data of all expressed genes. (B) Heat map of the Pearson correlation of sequenced samples based on gene expression level. Clustering was done according to the expression data of all expressed genes.
