# Supplementary material for: Transcriptomic Profile and Probiotic Properties of Lactiplantibacillus pentosus Pre-adapted to Edible Oils
Source: Front Microbiol. 2021 Oct 14;12:747043. doi: 10.3389/fmicb.2021.747043 (PMC8553220; doi:10.3389/fmicb.2021.747043)
Supplement: Supplementary file 2 [file Data_Sheet_2.PDF]

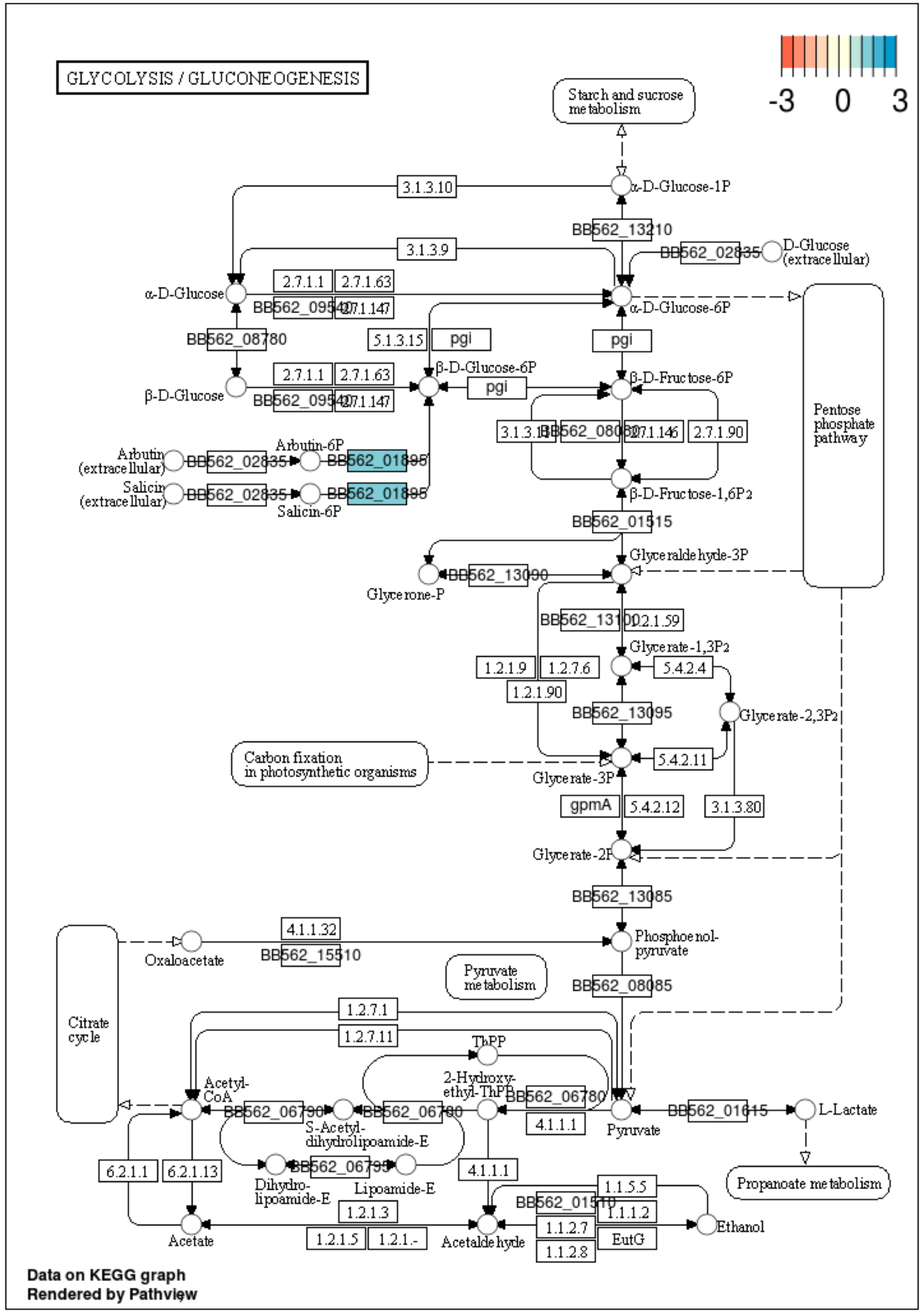
GLYCOLYSIS / GLUCONEOGENESIS
-3 0 3
Starch and sucrose metabolism
3.1.3.10
α-D-Glucose-1P
BB562_13210
3.1.3.9
BB562_02835
D-Glucose (extracellular)
α-D-Glucose
2.7.1.1
2.7.1.63
BB562_09540
2.7.1.147
α-D-Glucose-6P
BB562_08780
5.1.3.15
pgi
pgi
β-D-Glucose
2.7.1.1
2.7.1.63
BB562_09540
2.7.1.147
β-D-Glucose-6P
pgi
β-D-Fructose-6P
3.1.3.11
BB562_08060
2.7.1.146
2.7.1.90
Pentose phosphate pathway
Arbutin (extracellular)
BB562_02835
Arbutin-6P
BB562_01895
Salicin (extracellular)
BB562_02835
Salicin-6P
BB562_01895
β-D-Fructose-1,6P2
BB562_01515
Glyceraldehyde-3P
Glycerone-P
BB562_13090
BB562_13100
1.2.1.59
1.2.1.9
1.2.7.6
Glycerate-1,3P2
5.4.2.4
1.2.1.90
BB562_13095
Glycerate-2,3P2
Carbon fixation in photosynthetic organisms
Glycerate-3P
5.4.2.11
gpmA
5.4.2.12
3.1.3.80
Glycerate-2P
BB562_13085
4.1.1.32
Oxaloacetate
BB562_15510
Phosphoenol-pyruvate
Citrate cycle
Pyruvate metabolism
BB562_08085
1.2.7.1
1.2.7.11
ThPP
Acetyl-CoA
2-Hydroxy-ethyl-ThPP
BB562_06790
BB562_06780
BB562_06780
S-Acetyl-dihydrolipoamide-E
Pyruvate
BB562_01615
L-Lactate
4.1.1.1
6.2.1.1
6.2.1.13
Dihydro-lipoamide-E
BB562_06795
Lipoamide-E
4.1.1.1
Propanoate metabolism
1.1.5.5
BB562_01510
1.2.1.3
1.1.1.2
Acetate
1.2.1.5
1.2.1.-
Acetaldehyde
1.1.2.7
EutG
Ethanol
1.1.2.8
Data on KEGG graph
Rendered by Pathview

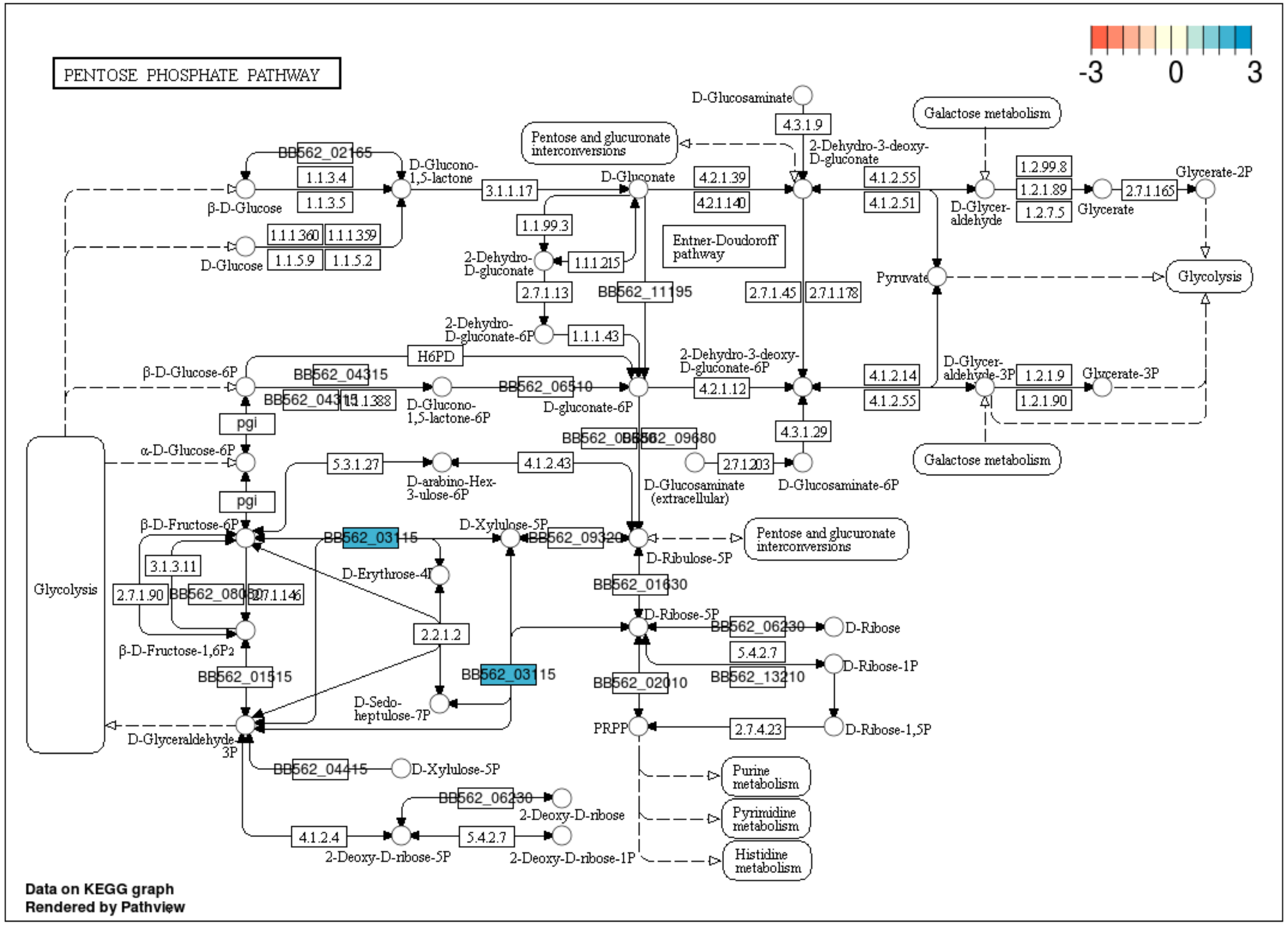
PENTOSE PHOSPHATE PATHWAY
-3 0 3
D-Glucosaminate
4.3.1.9
Galactose metabolism
Pentose and glucuronate interconversions
2-Dehydro-3-deoxy-D-gluconate
BB562_02165
D-Glucono-1,5-lactone
1.1.3.4
3.1.1.17
D-Gluconate
4.2.1.39
4.1.2.55
1.2.99.8
Glycerate-2P
β-D-Glucose
1.1.3.5
4.2.1.140
4.1.2.51
1.2.1.89
2.7.1.165
D-Glyceraldehyde
1.2.7.5
Glycerate
1.1.99.3
Entner-Doudoroff pathway
1.1.1.360
1.1.1.359
2-Dehydro-D-gluconate
1.1.1.215
Pyruvate
Glycolysis
D-Glucose
1.1.5.9
1.1.5.2
2.7.1.13
BB562_11195
2.7.1.45
2.7.1.178
2-Dehydro-D-gluconate-6P
1.1.1.43
H6PD
2-Dehydro-3-deoxy-D-gluconate-6P
β-D-Glucose-6P
BB562_04315
BB562_06510
4.2.1.12
4.1.2.14
D-Glyceraldehyde-3P
1.2.1.9
Glycerate-3P
BB562_04315
1.1.1.388
D-Glucono-1,5-lactone-6P
D-gluconate-6P
4.1.2.55
1.2.1.90
pgi
BB562_09680
4.3.1.29
Glycolysis
α-D-Glucose-6P
5.3.1.27
4.1.2.43
2.7.1.203
Galactose metabolism
D-arabino-Hex-3-ulose-6P
D-Glucosamine (extracellular)
D-Glucosamine-6P
pgi
β-D-Fructose-6P
BB562_03115
D-Xylulose-5P
BB562_09320
Pentose and glucuronate interconversions
D-Ribulose-5P
3.1.3.11
D-Erythrose-4P
2.7.1.90
BB562_08060
2.7.1.146
BB562_01630
D-Ribose-5P
BB562_06230
D-Ribose
β-D-Fructose-1,6P2
2.2.1.2
5.4.2.7
BB562_01515
BB562_03115
BB562_02010
BB562_13210
D-Ribose-1P
D-Sedo-heptulose-7P
PRPP
2.7.4.23
D-Ribose-1,5P
D-Glyceraldehyde-3P
BB562_04415
D-Xylulose-5P
Purine metabolism
BB562_06230
2-Deoxy-D-ribose
Pyrimidine metabolism
4.1.2.4
5.4.2.7
2-Deoxy-D-ribose-5P
2-Deoxy-D-ribose-1P
Histidine metabolism
Data on KEGG graph
Rendered by Pathview

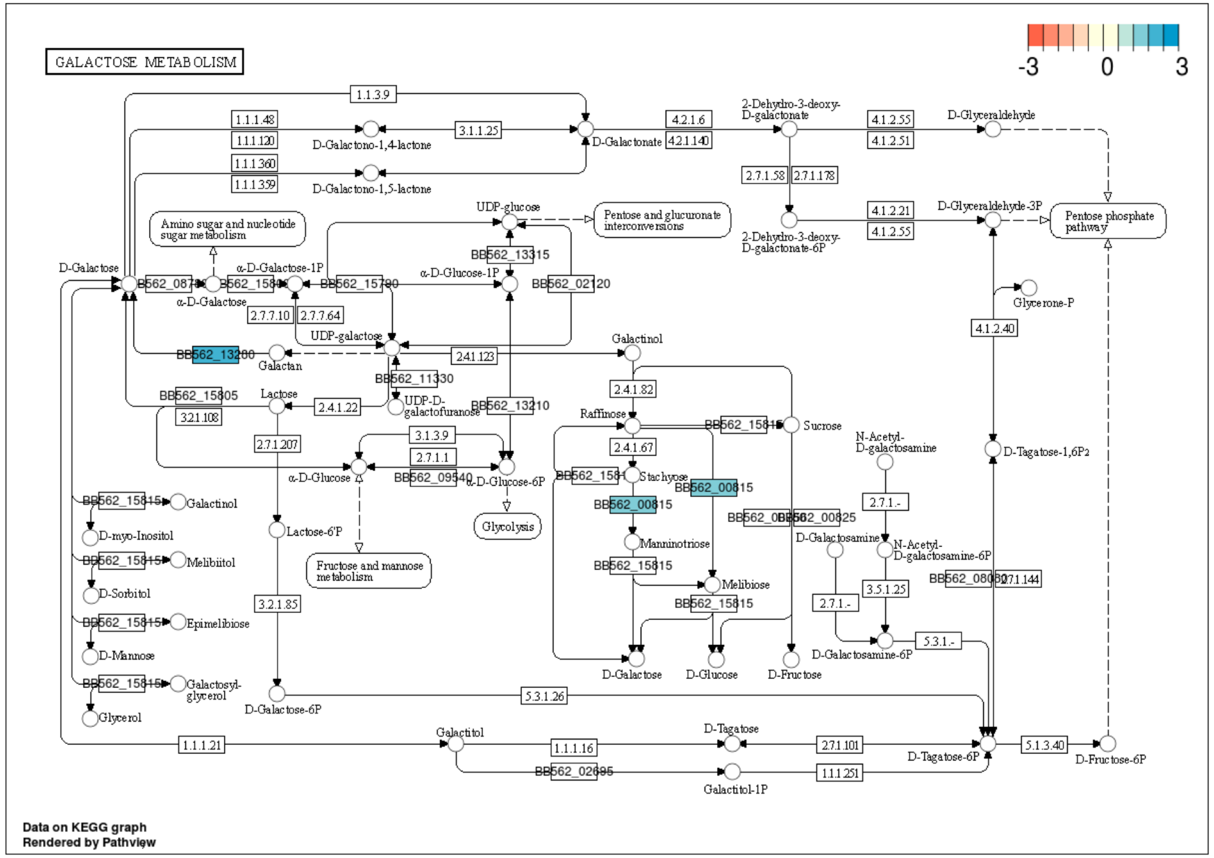
GALACTOSE METABOLISM
-3
0
3
D-Galactose
α-D-Galactose
α-D-Galactose-1P
UDP-glucose
α-D-Glucose-1P
UDP-galactose
Galactan
Lactose
Galactinol
Raffinose
Sucrose
Stachyose
Manninotriose
Melibiose
D-Galactose
D-Glucose
D-Fructose
α-D-Glucose
α-D-Glucose-6P
Glycolysis
Lactose-6P
D-Galactose-6P
D-Galactono-1,4-lactone
D-Galactono-1,5-lactone
D-Galactonate
2-Dehydro-3-deoxy-D-galactonate
2-Dehydro-3-deoxy-D-galactonate-6P
D-Glyceraldehyde
D-Glyceraldehyde-3P
Glycerone-P
D-Tagatose-1,6P2
D-Tagatose-6P
D-Tagatose
D-Fructose-6P
Galactitol
Galactitol-1P
N-Acetyl-D-galactosamine
N-Acetyl-D-galactosamine-6P
D-Galactosamine
D-Galactosamine-6P
D-myo-Inositol
D-Sorbitol
D-Mannose
Glycerol
Melibiitol
Epimelibiose
Galactosyl-glycerol
Amino sugar and nucleotide sugar metabolism
Pentose and glucuronate interconversions
Pentose phosphate pathway
Fructose and mannose metabolism
UDP-D-galactofuranose
Data on KEGG graph
Rendered by Pathview

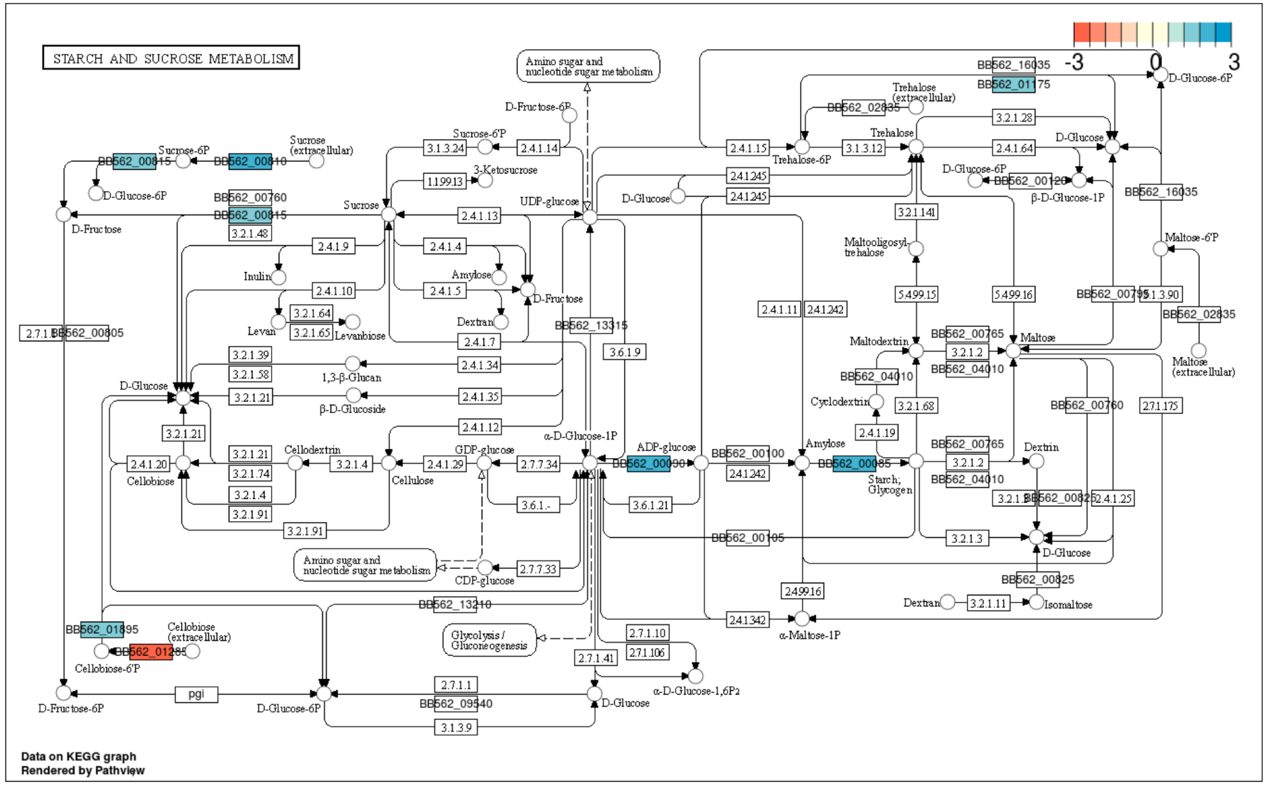
STARCH AND SUCROSE METABOLISM
-3
0
3
Sucrose-6P
Sucrose (extracellular)
D-Glucose-6P
D-Fructose
Sucrose
3-Ketosucrose
UDP-glucose
Inulin
Amylose
D-Fructose
Levan
Levanbiose
Dextran
1,3-β-Glucan
β-D-Glucoside
D-Glucose
Cellobiose
Cellodextrin
Cellulose
GDP-glucose
ADP-glucose
α-D-Glucose-1P
CDP-glucose
Trehalose-6P
Trehalose
Trehalose (extracellular)
Maltooligosyl-trehalose
Maltodextrin
Cyclodextrin
Maltose
Maltose-6'P
Maltose (extracellular)
Starch; Glycogen
Dextrin
Isomaltose
α-Maltose-1P
β-D-Glucose-1P
Cellobiose (extracellular)
Cellobiose-6'P
D-Fructose-6P
D-Glucose-6P
α-D-Glucose-1,6P2
Glycolysis / Gluconeogenesis
Amino sugar and nucleotide sugar metabolism
pgi
Data on KEGG graph
Rendered by Pathview

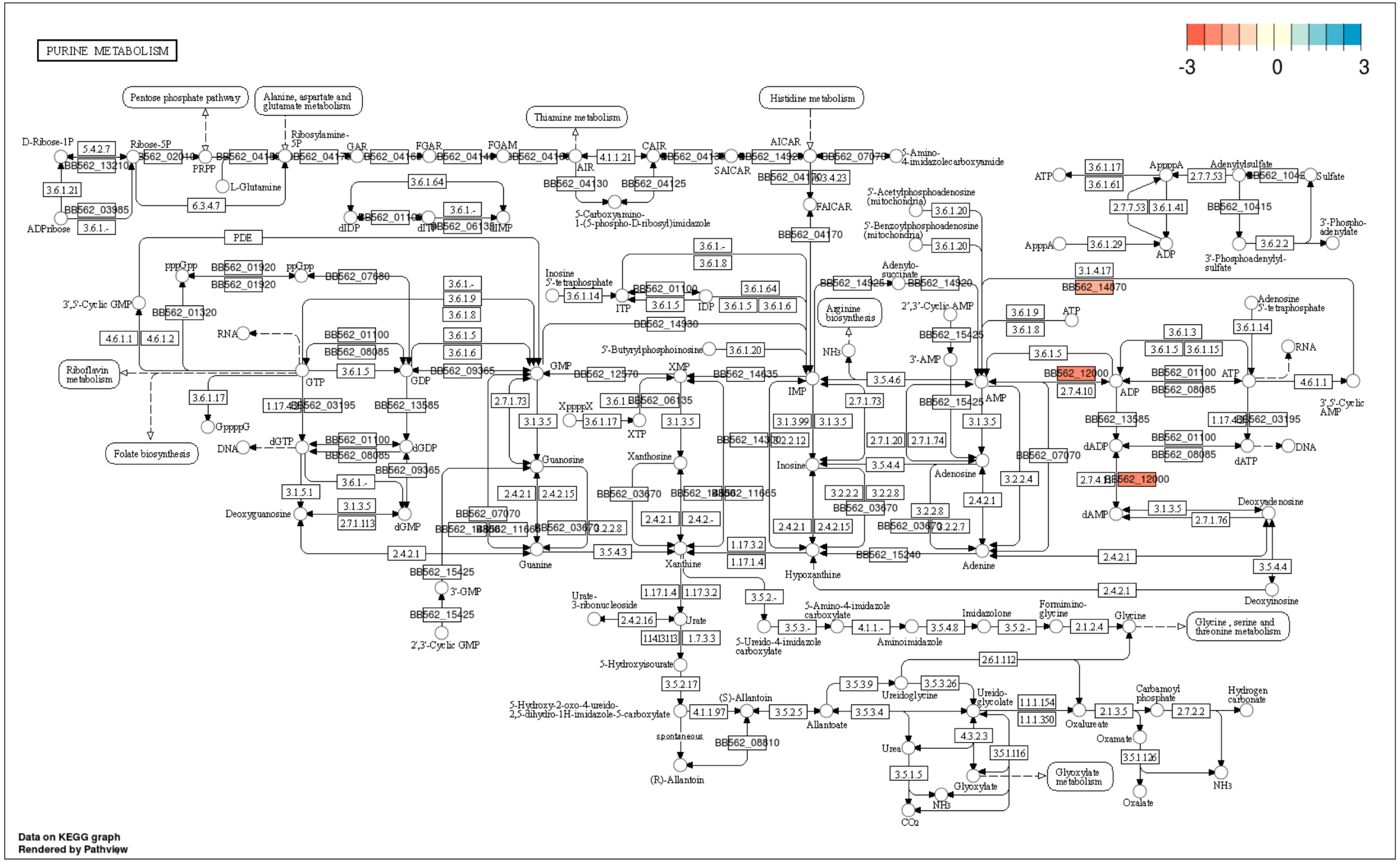
PURINE METABOLISM
-3
0
3
Pentose phosphate pathway
Alanine, aspartate and glutamate metabolism
Thiamine metabolism
Histidine metabolism
D-Ribose-1P
Ribose-5P
PRPP
L-Glutamine
Ribosylamine-5P
GAR
FGAR
FGAM
AIR
CAIR
SAICAR
AICAR
FAICAR
5-Amino-4-imidazolecarboxyamide
5-Carboxyamino-1-(5-phospho-D-ribosyl)imidazole
ADPribose
dIDP
dITP
dIMP
PDE
ppGpp
pppGpp
3',5'-Cyclic GMP
RNA
Riboflavin metabolism
Folate biosynthesis
GTP
GDP
GMP
GppppG
DNA
dGTP
dGDP
dGMP
Deoxyguanosine
Guanosine
Guanine
Inosine 5'-tetraphosphate
ITP
IDP
IMP
XMP
XTP
XppppX
Xanthosine
Xanthine
5'-Butyrylphosphoinosine
Arginine biosynthesis
NH3
Inosine
Hypoxanthine
Adenosine
Adenine
AMP
ADP
ATP
dADP
dATP
dAMP
Deoxyadenosine
Deoxyinosine
3'-AMP
2',3'-Cyclic AMP
Adenylo-succinate
5'-Acetylphosphoadenosine (mitochondria)
5'-Benzoylphosphoadenosine (mitochondria)
ApppA
AppppA
Adenylylsulfate
Sulfate
3'-Phosphoadenylylsulfate
3'-Phospho-adenylate
Adenosine 5'-tetraphosphate
3',5'-Cyclic AMP
3'-GMP
2',3'-Cyclic GMP
Urate-3-ribonucleoside
Urate
5-Hydroxyisourate
5-Hydroxy-2-oxo-4-ureido-2,5-dihydro-1H-imidazole-5-carboxylate
(S)-Allantoin
(R)-Allantoin
spontaneous
Allantoate
Ureidoglycine
Ureidoglycolate
Urea
Glyoxylate
Glyoxylate metabolism
NH3
CO2
Oxalureate
Oxamate
Oxalate
Carbamoyl phosphate
Hydrogen carbonate
5-Ureido-4-imidazole carboxylate
5-Amino-4-imidazole carboxylate
Aminoimidazole
Imidazolone
Formimino-glycine
Glycine
Glycine, serine and threonine metabolism
BB562_14870
BB562_12000
Data on KEGG graph
Rendered by Pathview

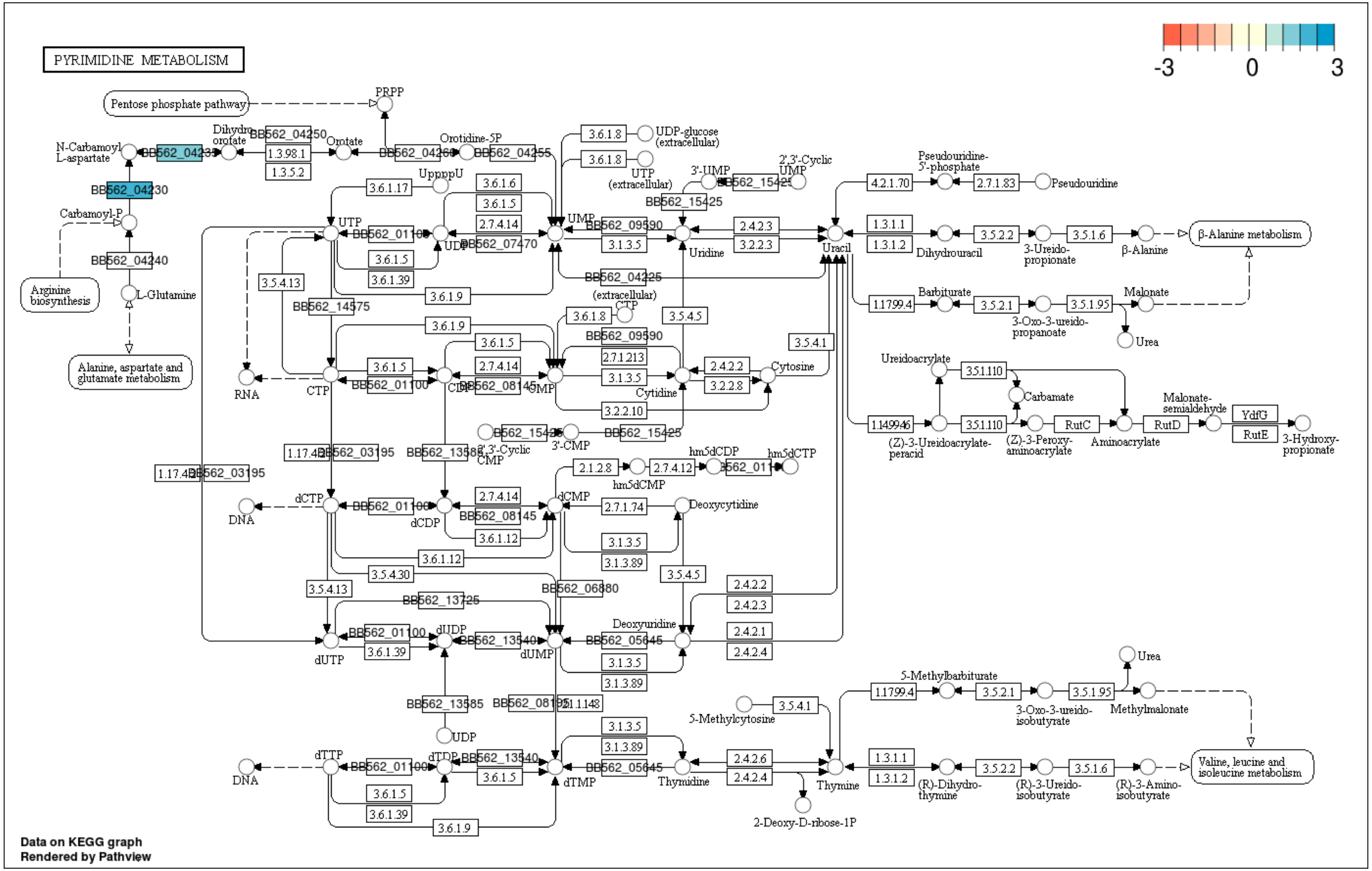
PYRIMIDINE METABOLISM
-3
0
3
Pentose phosphate pathway
PRPP
N-Carbamoyl-L-aspartate
Dihydro-orotate
Orotate
Orotidine-5P
UMP
UTP
UDP
CTP
CDP
CMP
RNA
DNA
dCTP
dCDP
dCMP
dUTP
dUDP
dUMP
dTTP
dTDP
dTMP
Uridine
Uracil
Cytidine
Cytosine
Deoxycytidine
Deoxyuridine
Thymidine
Thymine
Carbamoyl-P
L-Glutamine
Arginine biosynthesis
Alanine, aspartate and glutamate metabolism
β-Alanine metabolism
Valine, leucine and isoleucine metabolism
Data on KEGG graph
Rendered by Pathview

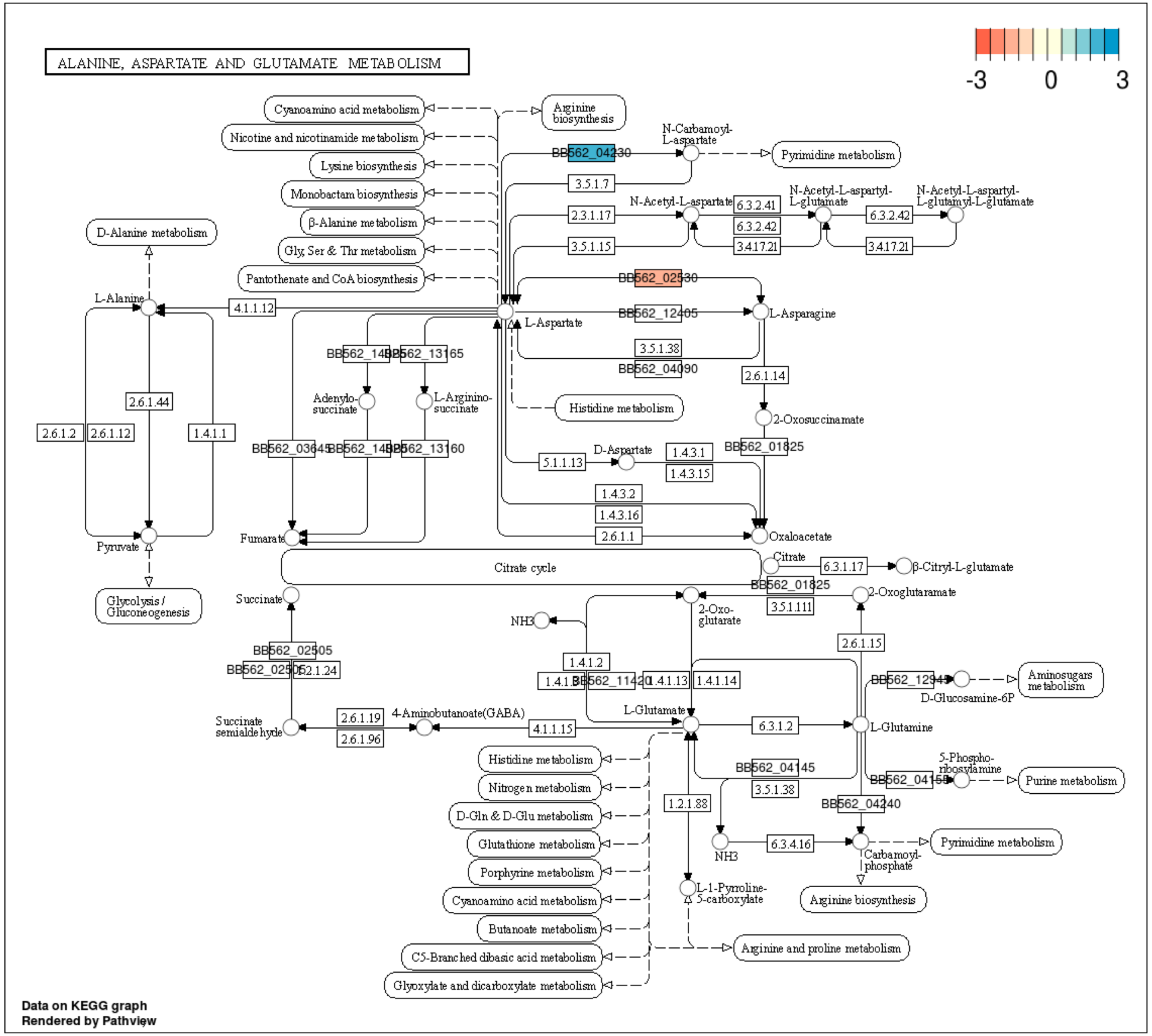
ALANINE, ASPARTATE AND GLUTAMATE METABOLISM
-3
0
3
Cyanoamino acid metabolism
Nicotine and nicotinamide metabolism
Lysine biosynthesis
Monobactam biosynthesis
β-Alanine metabolism
Gly, Ser & Thr metabolism
Pantothenate and CoA biosynthesis
Arginine biosynthesis
N-Carbamoyl-L-aspartate
Pyrimidine metabolism
BB562_04230
3.5.1.7
2.3.1.17
3.5.1.15
N-Acetyl-L-aspartate
6.3.2.41
6.3.2.42
3.4.17.21
N-Acetyl-L-aspartyl-L-glutamate
6.3.2.42
3.4.17.21
N-Acetyl-L-aspartyl-L-glutamyl-L-glutamate
D-Alanine metabolism
L-Alanine
4.1.1.12
L-Aspartate
BB562_02530
BB562_12405
L-Asparagine
3.5.1.38
BB562_04090
BB562_13165
Adenylo-succinate
L-Arginino-succinate
Histidine metabolism
2.6.1.44
2.6.1.2
2.6.1.12
1.4.1.1
2.6.1.14
2-Oxosuccinamate
BB562_03645
BB562_13160
BB562_01825
D-Aspartate
5.1.1.13
1.4.3.1
1.4.3.15
1.4.3.2
1.4.3.16
2.6.1.1
Oxaloacetate
Pyruvate
Fumarate
Citrate cycle
Citrate
6.3.1.17
β-Citryl-L-glutamate
Glycolysis / Gluconeogenesis
Succinate
2-Oxoglutaramate
3.5.1.111
2-Oxo-glutarate
NH3
2.6.1.15
BB562_02505
1.4.1.2
1.4.1.13
1.4.1.14
BB562_11420
BB562_12945
Aminosugars metabolism
D-Glucosamine-6P
Succinate semialdehyde
2.6.1.19
2.6.1.96
4-Aminobutanoate(GABA)
4.1.1.15
L-Glutamate
6.3.1.2
L-Glutamine
5-Phospho-ribosylamine
Purine metabolism
Histidine metabolism
Nitrogen metabolism
D-Gln & D-Glu metabolism
Glutathione metabolism
Porphyrine metabolism
Cyanoamino acid metabolism
Butanoate metabolism
C5-Branched dibasic acid metabolism
Glyoxylate and dicarboxylate metabolism
BB562_04145
3.5.1.38
1.2.1.88
BB562_04240
6.3.4.16
Carbamoyl-phosphate
Pyrimidine metabolism
Arginine biosynthesis
L-1-Pyrroline-5-carboxylate
Arginine and proline metabolism
Data on KEGG graph
Rendered by Pathview

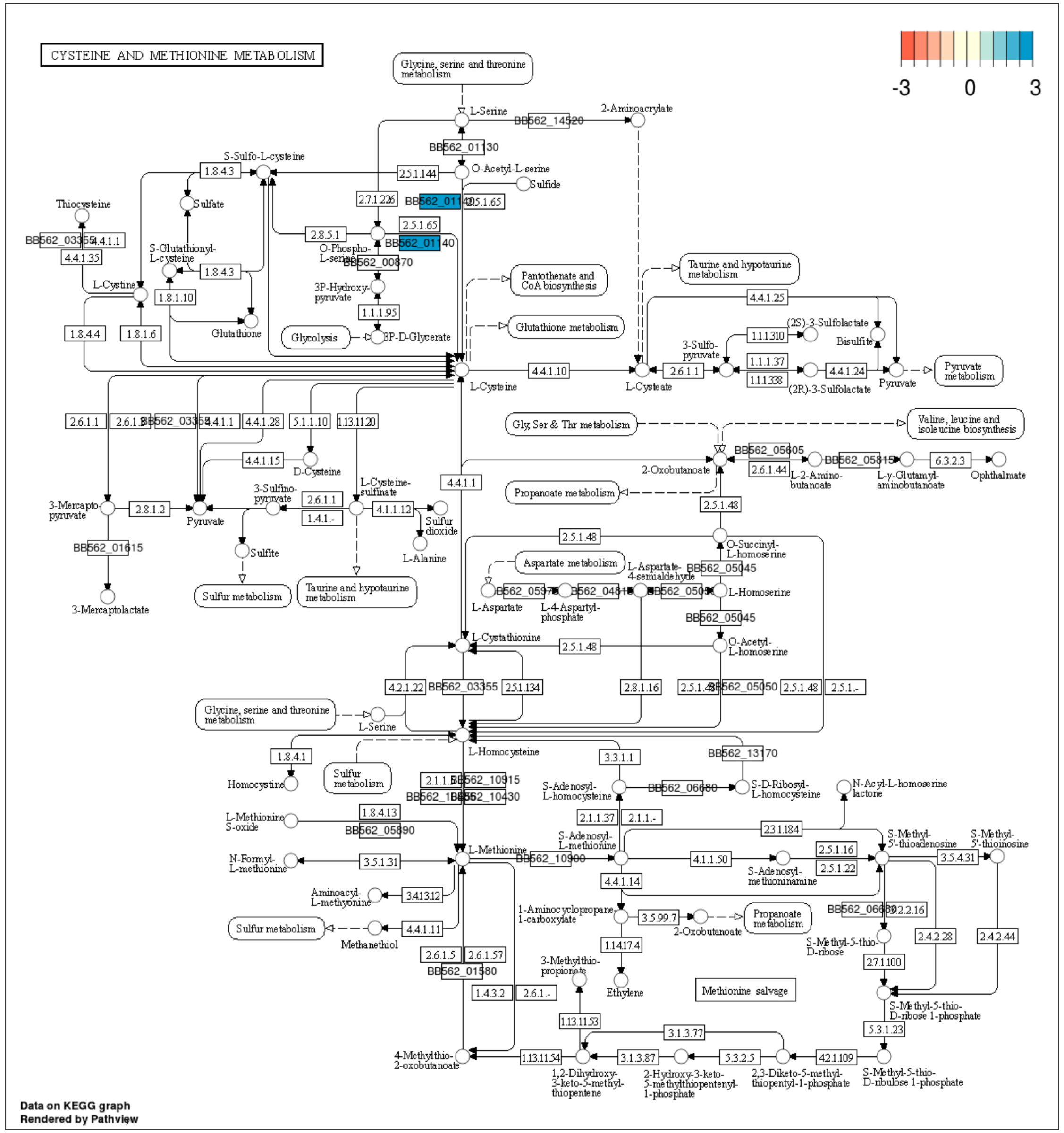
CYSTEINE AND METHIONINE METABOLISM
-3
0
3
Glycine, serine and threonine metabolism
L-Serine
BB562_14520
2-Aminoacrylate
BB562_01130
O-Acetyl-L-serine
Sulfide
S-Sulfo-L-cysteine
1.8.4.3
2.5.1.144
2.7.1.226
BB562_01140
2.5.1.65
Thiocysteine
Sulfate
4.4.1.35
S-Glutathionyl-L-cysteine
2.8.5.1
O-Phospho-L-serine
BB562_00870
L-Cystine
1.8.1.10
3P-Hydroxy-pyruvate
1.1.1.95
Glutathione
Glycolysis
3P-D-Glycerate
Pantothenate and CoA biosynthesis
Glutathione metabolism
Taurine and hypotaurine metabolism
4.4.1.25
(2S)-3-Sulfolactate
Bisulfite
3-Sulfo-pyruvate
1.1.1.310
1.1.1.37
1.1.1.338
4.4.1.24
(2R)-3-Sulfolactate
Pyruvate
Pyruvate metabolism
1.8.4.4
1.8.1.6
L-Cysteine
4.4.1.10
L-Cysteate
2.6.1.1
Gly, Ser & Thr metabolism
Valine, leucine and isoleucine biosynthesis
BB562_05605
2-Oxobutanoate
BB562_05815
6.3.2.3
L-2-Amino-butanoate
L-γ-Glutamyl-aminobutanoate
Ophthalmate
2.6.1.44
Propanoate metabolism
2.5.1.48
4.4.1.15
D-Cysteine
5.1.1.10
1.13.11.20
3-Mercapto-pyruvate
2.8.1.2
Pyruvate
3-Sulfino-pyruvate
L-Cysteine-sulfinate
4.1.1.12
Sulfur dioxide
L-Alanine
Sulfite
BB562_01615
3-Mercaptolactate
Sulfur metabolism
Taurine and hypotaurine metabolism
Aspartate metabolism
O-Succinyl-L-homoserine
L-Aspartate-4-semialdehyde
BB562_05045
L-Homoserine
L-Aspartate
L-4-Aspartyl-phosphate
L-Cystathionine
O-Acetyl-L-homoserine
4.2.1.22
BB562_03355
2.5.1.134
2.8.1.16
BB562_05050
Glycine, serine and threonine metabolism
L-Serine
L-Homocysteine
1.8.4.1
Homocysteine
Sulfur metabolism
3.3.1.1
BB562_13170
S-Adenosyl-L-homocysteine
BB562_06680
S-D-Ribosyl-L-homocysteine
N-Acyl-L-homoserine lactone
BB562_10915
BB562_10430
L-Methionine S-oxide
1.8.4.13
BB562_05890
2.1.1.37
2.1.1.-
2.3.1.184
S-Adenosyl-L-methionine
S-Methyl-5'-thioadenosine
S-Methyl-5'-thioinosine
N-Formyl-L-methionine
3.5.1.31
L-Methionine
BB562_10900
4.1.1.50
S-Adenosyl-methioninamine
2.5.1.16
2.5.1.22
3.5.4.31
4.4.1.14
Aminoacyl-L-methionine
3.4.13.12
1-Aminocyclopropane-1-carboxylate
3.5.99.7
2-Oxobutanoate
Propanoate metabolism
BB562_06685
Sulfur metabolism
4.4.1.11
Methanethiol
1.14.17.4
Ethylene
S-Methyl-5-thio-D-ribose
2.4.2.28
2.4.2.44
2.7.1.100
2.6.1.5
2.6.1.57
BB562_01580
3-Methylthio-propionate
1.4.3.2
2.6.1.-
Methionine salvage
S-Methyl-5-thio-D-ribose 1-phosphate
1.13.11.53
3.1.3.77
5.3.1.23
4-Methylthio-2-oxobutanoate
1.13.11.54
3.1.3.87
5.3.2.5
4.2.1.109
1,2-Dihydroxy-3-keto-5-methyl-thiopentene
2-Hydroxy-3-keto-5-methylthiopentenyl-1-phosphate
2,3-Diketo-5-methyl-thiopentyl-1-phosphate
S-Methyl-5-thio-D-ribulose 1-phosphate
Data on KEGG graph
Rendered by Pathview

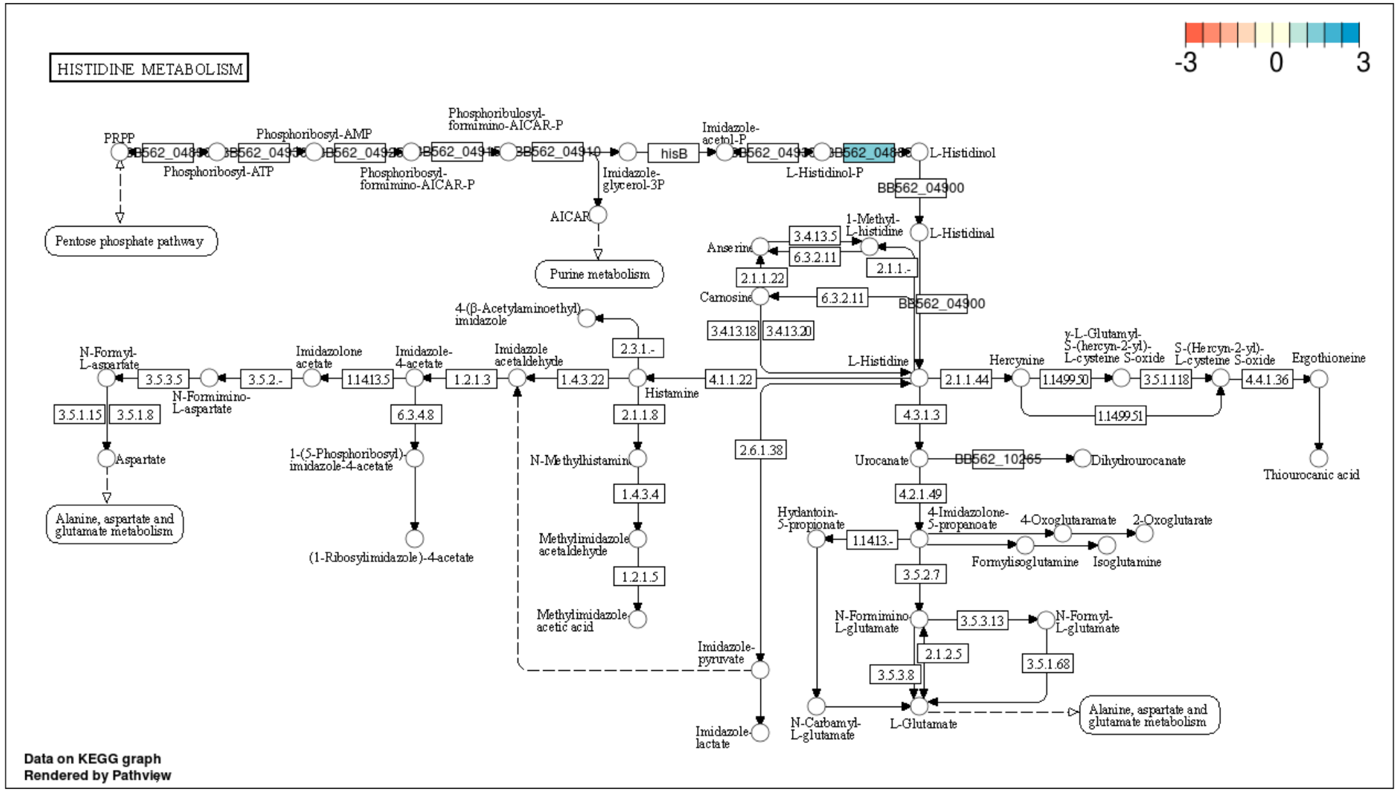
HISTIDINE METABOLISM
-3
0
3
PRPP
Phosphoribosyl-AMP
Phosphoribosyl-ATP
Phosphoribulosyl-formimino-AICAR-P
Phosphoribosyl-formimino-AICAR-P
Imidazole-acetol-P
hisB
Imidazole-glycerol-3P
L-Histidinol-P
L-Histidinol
BB562_04900
L-Histidinal
AICAR
Purine metabolism
Pentose phosphate pathway
1-Methyl-L-histidine
Anserine
3.4.13.5
6.3.2.11
2.1.1.-
2.1.1.22
Carnosine
6.3.2.11
BB562_04900
3.4.13.18
3.4.13.20
4-(β-Acetylaminoethyl)imidazole
2.3.1.-
N-Formyl-L-aspartate
3.5.3.5
3.5.2.-
N-Formimino-L-aspartate
Imidazolone acetate
1.14.13.5
Imidazole-4-acetate
1.2.1.3
Imidazole acetaldehyde
1.4.3.22
Histamine
4.1.1.22
L-Histidine
2.1.1.44
Hercynine
1.14.99.50
γ-L-Glutamyl-S-(hercyn-2-yl)-L-cysteine S-oxide
3.5.1.118
S-(Hercyn-2-yl)-L-cysteine S-oxide
4.4.1.36
Ergothioneine
1.14.99.51
Thiourocanic acid
3.5.1.15
3.5.1.8
Aspartate
Alanine, aspartate and glutamate metabolism
6.3.4.8
1-(5-Phosphoribosyl)-imidazole-4-acetate
(1-Ribosylimidazole)-4-acetate
2.1.1.8
N-Methylhistamine
1.4.3.4
Methylimidazole acetaldehyde
1.2.1.5
Methylimidazole-acetic acid
2.6.1.38
4.3.1.3
Urocanate
BB562_10265
Dihydrourocanate
4.2.1.49
Hydantoin-5-propionate
1.14.13.-
4-Imidazolone-5-propanoate
4-Oxoglutaramate
2-Oxoglutarate
Formylisoglutamine
Isoglutamine
3.5.2.7
N-Formimino-L-glutamate
3.5.3.13
N-Formyl-L-glutamate
2.1.2.5
3.5.3.8
3.5.1.68
Imidazole-pyruvate
N-Carbamyl-L-glutamate
L-Glutamate
Alanine, aspartate and glutamate metabolism
Imidazole-lactate
Data on KEGG graph
Rendered by Pathview

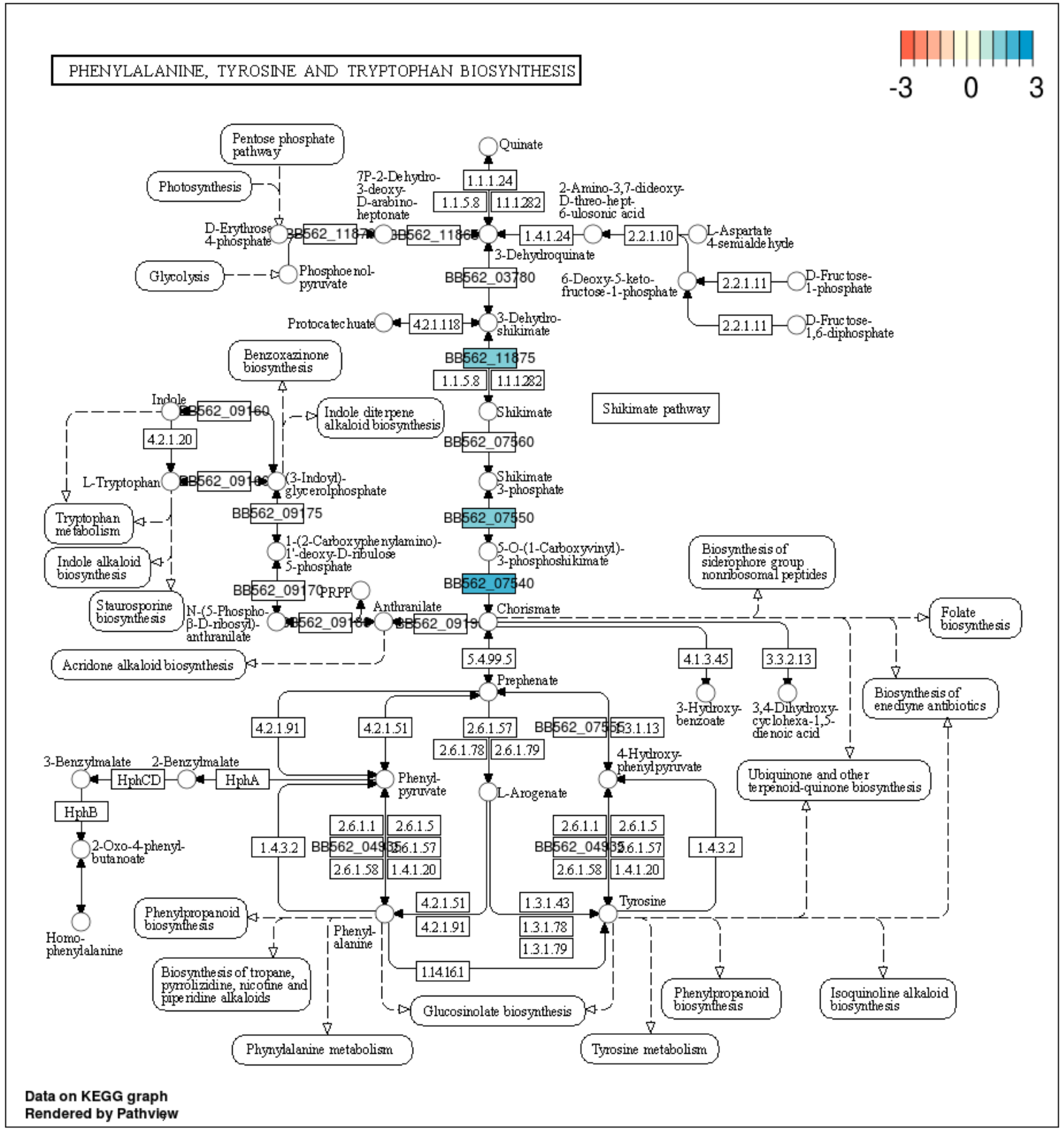
PHENYLALANINE, TYROSINE AND TRYPTOPHAN BIOSYNTHESIS
-3
0
3
Pentose phosphate pathway
Photosynthesis
Quinate
1.1.1.24
7P-2-Dehydro-3-deoxy-D-arabino-heptonate
1.1.5.8
1.1.1.282
2-Amino-3,7-dideoxy-D-threo-hept-6-ulosonic acid
D-Erythrose 4-phosphate
1.4.1.24
2.2.1.10
L-Aspartate 4-semialdehyde
3-Dehydroquinate
Glycolysis
Phosphoenol-pyruvate
BB562_03780
6-Deoxy-5-keto-fructose-1-phosphate
2.2.1.11
D-Fructose-1-phosphate
2.2.1.11
D-Fructose-1,6-diphosphate
Protocatechuate
4.2.1.118
3-Dehydro-shikimate
Benzoxazinone biosynthesis
BB562_11875
1.1.5.8
1.1.1.282
Indole
BB562_09160
Indole diterpene alkaloid biosynthesis
Shikimate
Shikimate pathway
4.2.1.20
BB562_07560
L-Tryptophan
(3-Indolyl)-glycerolphosphate
Shikimate 3-phosphate
Tryptophan metabolism
BB562_09175
BB562_07550
1-(2-Carboxyphenylamino)-1'-deoxy-D-ribulose 5-phosphate
5-O-(1-Carboxyvinyl)-3-phosphoshikimate
Indole alkaloid biosynthesis
Biosynthesis of siderophore group nonribosomal peptides
BB562_09170
PRPP
BB562_07540
Staurosporine biosynthesis
N-(5-Phospho-β-D-ribosyl)-anthranilate
Anthranilate
Chorismate
Folate biosynthesis
Acridone alkaloid biosynthesis
5.4.99.5
4.1.3.45
3.3.2.13
Prephenate
3-Hydroxy-benzoate
3,4-Dihydroxy-cyclohexa-1,5-diene-1-carboxylic acid
Biosynthesis of enediyne antibiotics
4.2.1.91
4.2.1.51
2.6.1.57
5.3.1.13
2.6.1.78
2.6.1.79
4-Hydroxy-phenylpyruvate
3-Benzylmalate
2-Benzylmalate
HphCD
HphA
Phenyl-pyruvate
L-Arogenate
Ubiquinone and other terpenoid-quinone biosynthesis
HphB
2.6.1.1
2.6.1.5
2.6.1.1
2.6.1.5
1.4.3.2
2.6.1.57
2.6.1.57
1.4.3.2
2-Oxo-4-phenyl-butanoate
2.6.1.58
1.4.1.20
2.6.1.58
1.4.1.20
4.2.1.51
1.3.1.43
Tyrosine
4.2.1.91
1.3.1.78
Phenylpropanoid biosynthesis
Phenyl-alanine
1.3.1.79
Homo-phenylalanine
1.14.16.1
Biosynthesis of tropane, pyrrolizidine, nicotine and piperidine alkaloids
Glucosinolate biosynthesis
Phenylpropanoid biosynthesis
Isoquinoline alkaloid biosynthesis
Phynylalanine metabolism
Tyrosine metabolism
Data on KEGG graph
Rendered by Pathview

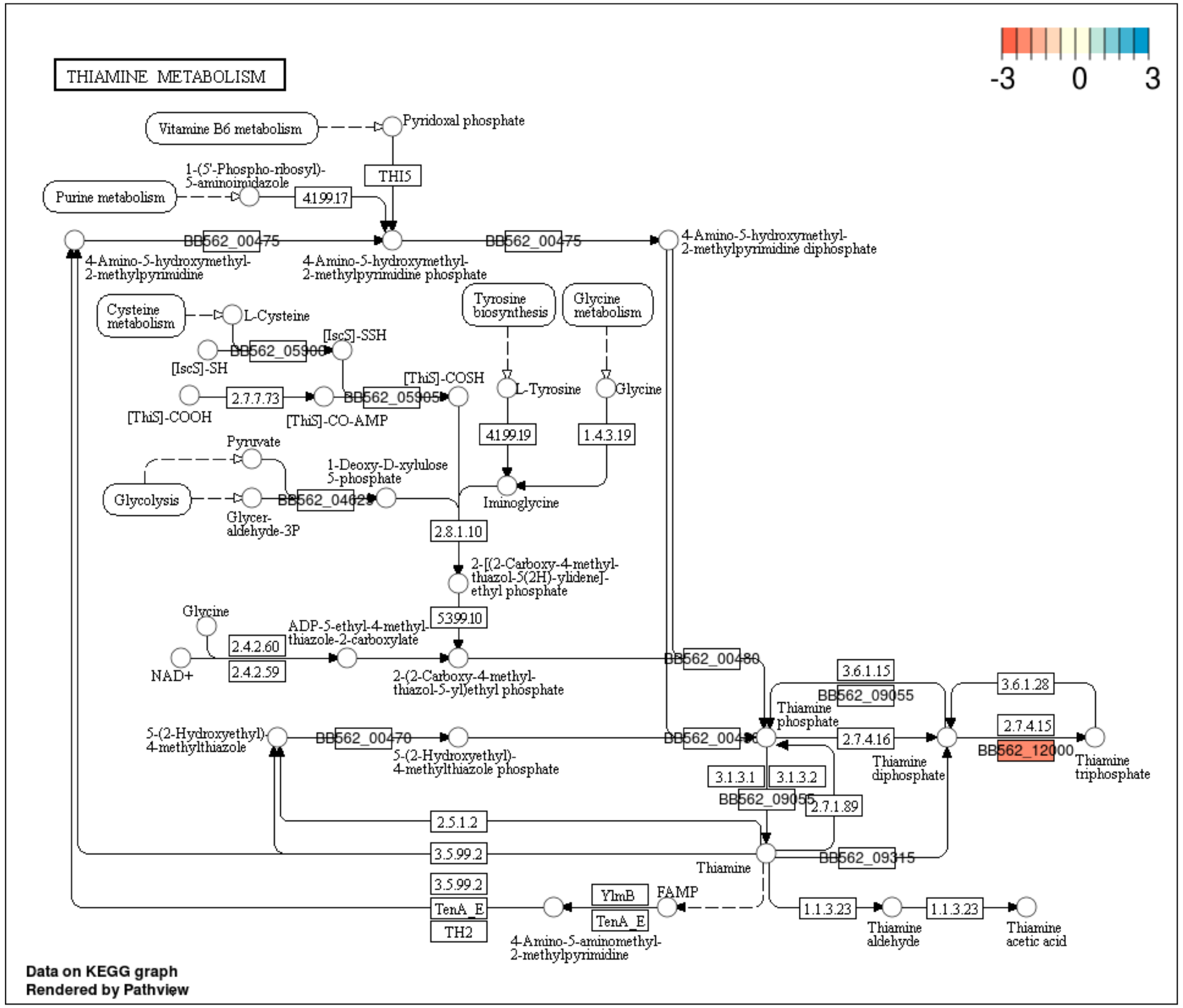
THIAMINE METABOLISM
-3
0
3
Vitamine B6 metabolism
Pyridoxal phosphate
THI5
Purine metabolism
1-(5'-Phospho-ribosyl)-5-aminoimidazole
4.1.99.17
BB562_00475
4-Amino-5-hydroxymethyl-2-methylpyrimidine
4-Amino-5-hydroxymethyl-2-methylpyrimidine phosphate
4-Amino-5-hydroxymethyl-2-methylpyrimidine diphosphate
Cysteine metabolism
L-Cysteine
[IscS]-SSH
[IscS]-SH
Tyrosine biosynthesis
Glycine metabolism
[ThiS]-COSH
L-Tyrosine
Glycine
2.7.7.73
[ThiS]-COOH
[ThiS]-CO-AMP
4.1.99.19
1.4.3.19
Pyruvate
1-Deoxy-D-xylulose 5-phosphate
Iminoglycine
Glycolysis
Glycer-aldehyde-3P
2.8.1.10
2-[(2-Carboxy-4-methyl-thiazol-5(2H)-ylidene]-ethyl phosphate
5.3.99.10
ADP-5-ethyl-4-methyl-thiazole-2-carboxylate
2.4.2.60
2.4.2.59
NAD+
2-(2-Carboxy-4-methyl-thiazol-5-yl)ethyl phosphate
3.6.1.15
BB562_09055
3.6.1.28
Thiamine phosphate
2.7.4.15
5-(2-Hydroxyethyl)-4-methylthiazole
BB562_00470
5-(2-Hydroxyethyl)-4-methylthiazole phosphate
2.7.4.16
Thiamine diphosphate
BB562_12000
Thiamine triphosphate
3.1.3.1
3.1.3.2
2.7.1.89
2.5.1.2
3.5.99.2
Thiamine
BB562_09315
YlmB
FAMP
TenA_E
TH2
1.1.3.23
Thiamine aldehyde
Thiamine acetic acid
4-Amino-5-aminomethyl-2-methylpyrimidine
Data on KEGG graph
Rendered by Pathview

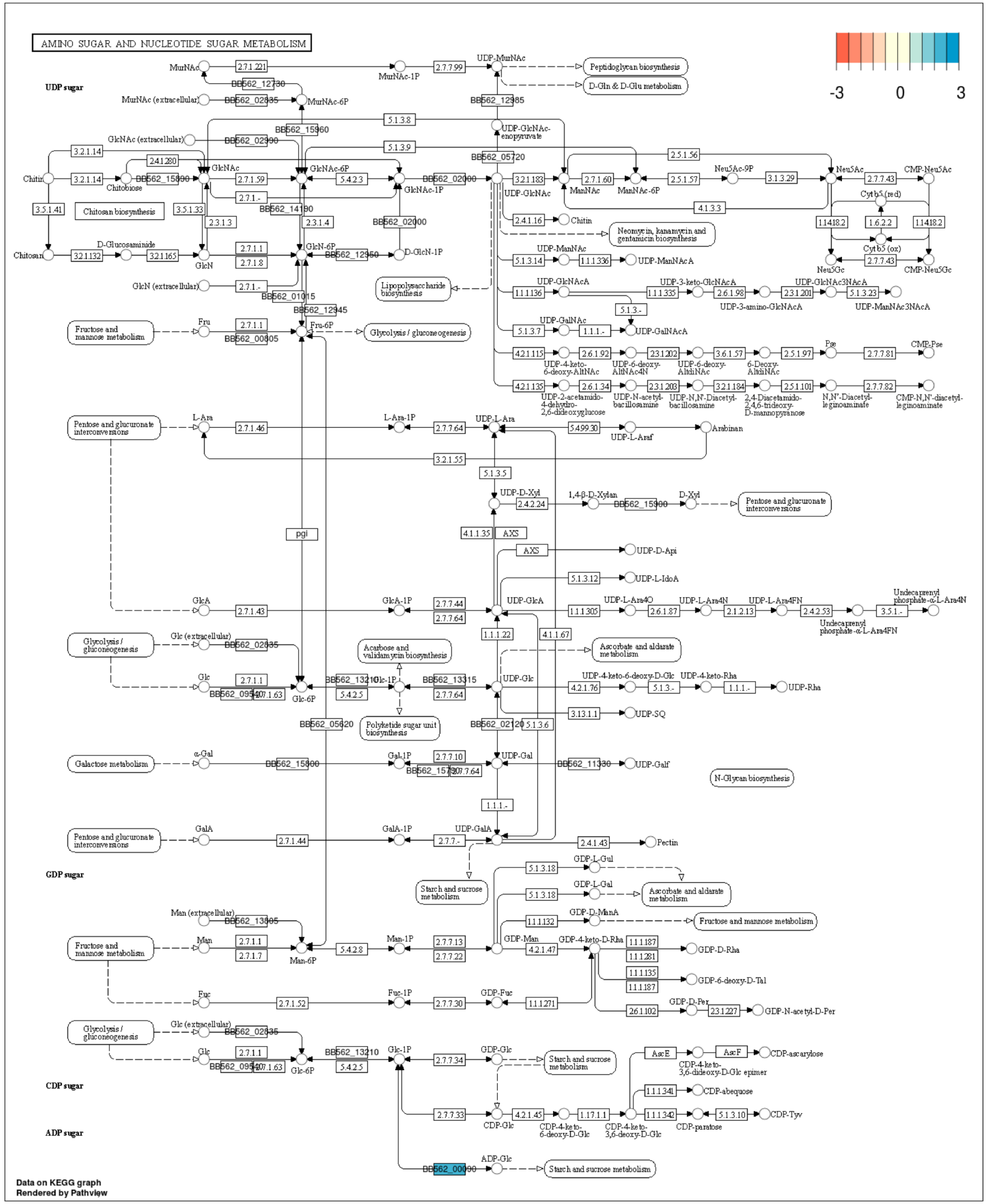
AMINO SUGAR AND NUCLEOTIDE SUGAR METABOLISM
-3
0
3
UDP sugar
GDP sugar
CDP sugar
ADP sugar
MurNAc
MurNAc-1P
UDP-MurNAc
MurNAc (extracellular)
MurNAc-6P
Peptidoglycan biosynthesis
D-Gln & D-Glu metabolism
GlcNAc (extracellular)
UDP-GlcNAc-enopyruvate
Chitin
Chitobiose
GlcNAc
GlcNAc-6P
GlcNAc-1P
UDP-GlcNAc
ManNAc
ManNAc-6P
Neu5Ac-9P
Neu5Ac
CMP-Neu5Ac
Cytb5 (red)
Cytb5 (ox)
Neu5Gc
CMP-Neu5Gc
Chitosan biosynthesis
Chitosan
D-Glucosaminide
GlcN
GlcN-6P
D-GlcN-1P
GlcN (extracellular)
Neomycin, kanamycin and gentamicin biosynthesis
UDP-ManNAc
UDP-ManNAcA
Lipopolysaccharide biosynthesis
UDP-GlcNAcA
UDP-3-keto-GlcNAcA
UDP-3-amino-GlcNAcA
UDP-GlcNAc3NAcA
UDP-ManNAc3NAcA
UDP-GalNAc
UDP-GalNAcA
Fructose and mannose metabolism
Fru
Fru-6P
Glycolysis / gluconeogenesis
UDP-4-keto-6-deoxy-AltNAc
UDP-6-deoxy-AltdiNAc4N
UDP-6-deoxy-AltdiNAc
6-Deoxy-AltdiNAc
Pse
CMP-Pse
UDP-2-acetamido-4-dehydro-2,6-dideoxyglucose
UDP-N-acetyl-bacillosamine
UDP-N,N'-Diacetyl-bacillosamine
2,4-Diacetamido-2,4,6-trideoxy-D-mannopyranose
N,N'-Diacetyl-legionaminate
CMP-N,N'-diacetyl-legionaminate
Pentose and glucuronate interconversions
L-Ara
L-Ara-1P
UDP-L-Ara
UDP-L-Araf
Arabinan
UDP-D-Xyl
1,4-β-D-Xylan
D-Xyl
pgl
AXS
UDP-D-Api
UDP-L-IdoA
GlcA
GlcA-1P
UDP-GlcA
UDP-L-Ara4O
UDP-L-Ara4N
UDP-L-Ara4FN
Undecaprenyl phosphate-α-L-Ara4FN
Undecaprenyl phosphate-α-L-Ara4N
Glc (extracellular)
Acarbose and validamycin biosynthesis
Ascorbate and aldarate metabolism
Glc
Glc-6P
Glc-1P
UDP-Glc
UDP-4-keto-6-deoxy-D-Glc
UDP-4-keto-Rha
UDP-Rha
UDP-SQ
Polyketide sugar unit biosynthesis
Galactose metabolism
α-Gal
Gal-1P
UDP-Gal
UDP-Galf
N-Glycan biosynthesis
GalA
GalA-1P
UDP-GalA
Pectin
GDP-L-Gul
GDP-L-Gal
Starch and sucrose metabolism
GDP-D-ManA
Man (extracellular)
Man
Man-6P
Man-1P
GDP-Man
GDP-4-keto-D-Rha
GDP-D-Rha
GDP-6-deoxy-D-Tal
Fuc
Fuc-1P
GDP-Fuc
GDP-D-Per
GDP-N-acetyl-D-Per
GDP-Glc
AscE
AscF
CDP-4-keto-3,6-dideoxy-D-Glc epimer
CDP-ascarylose
CDP-abequose
CDP-Glc
CDP-4-keto-6-deoxy-D-Glc
CDP-4-keto-3,6-deoxy-D-Glc
CDP-paratose
CDP-Tyv
ADP-Glc
BB562_00090
Data on KEGG graph
Rendered by Pathview

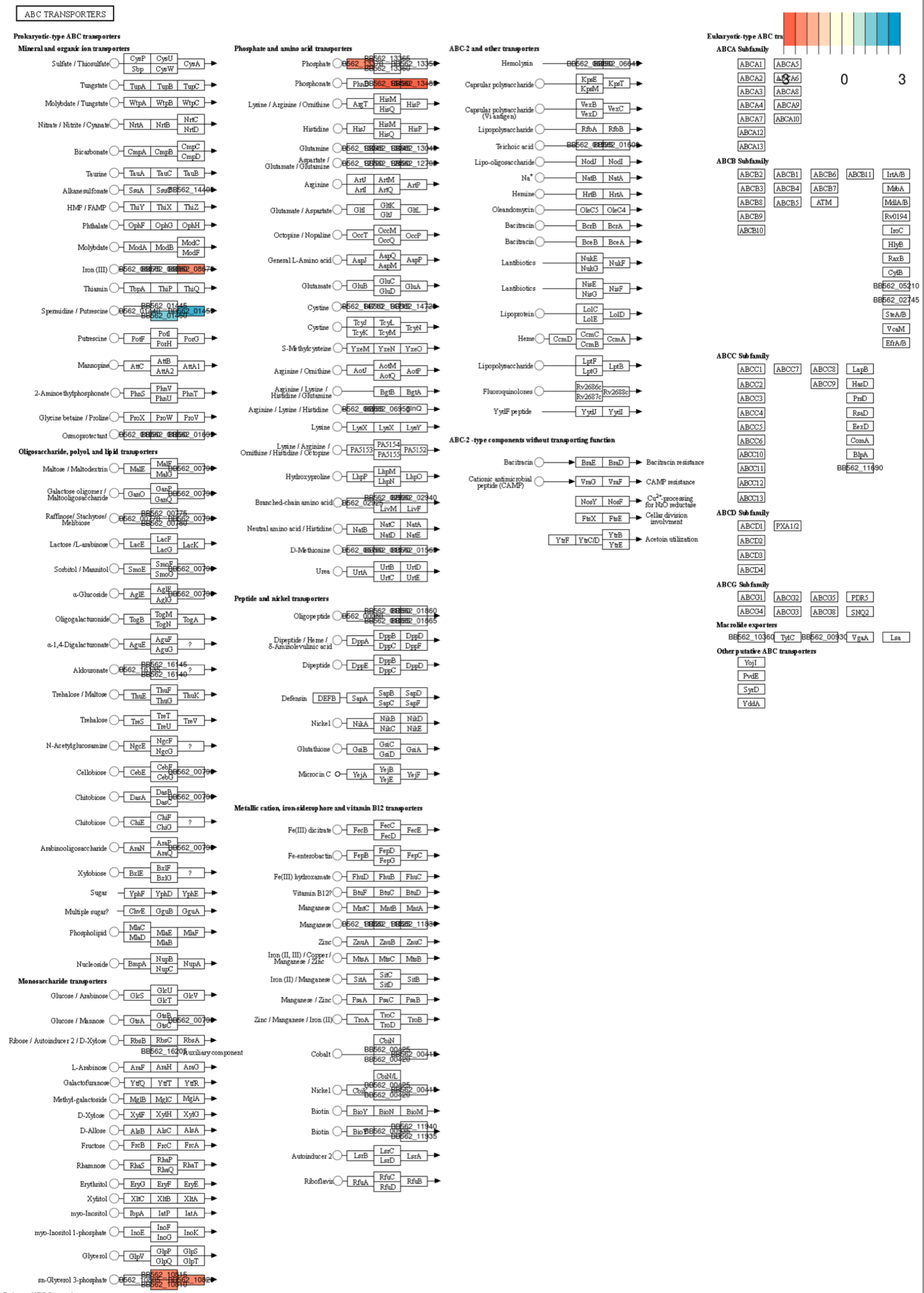

Data on KEGG graph
Rendered by Pathview

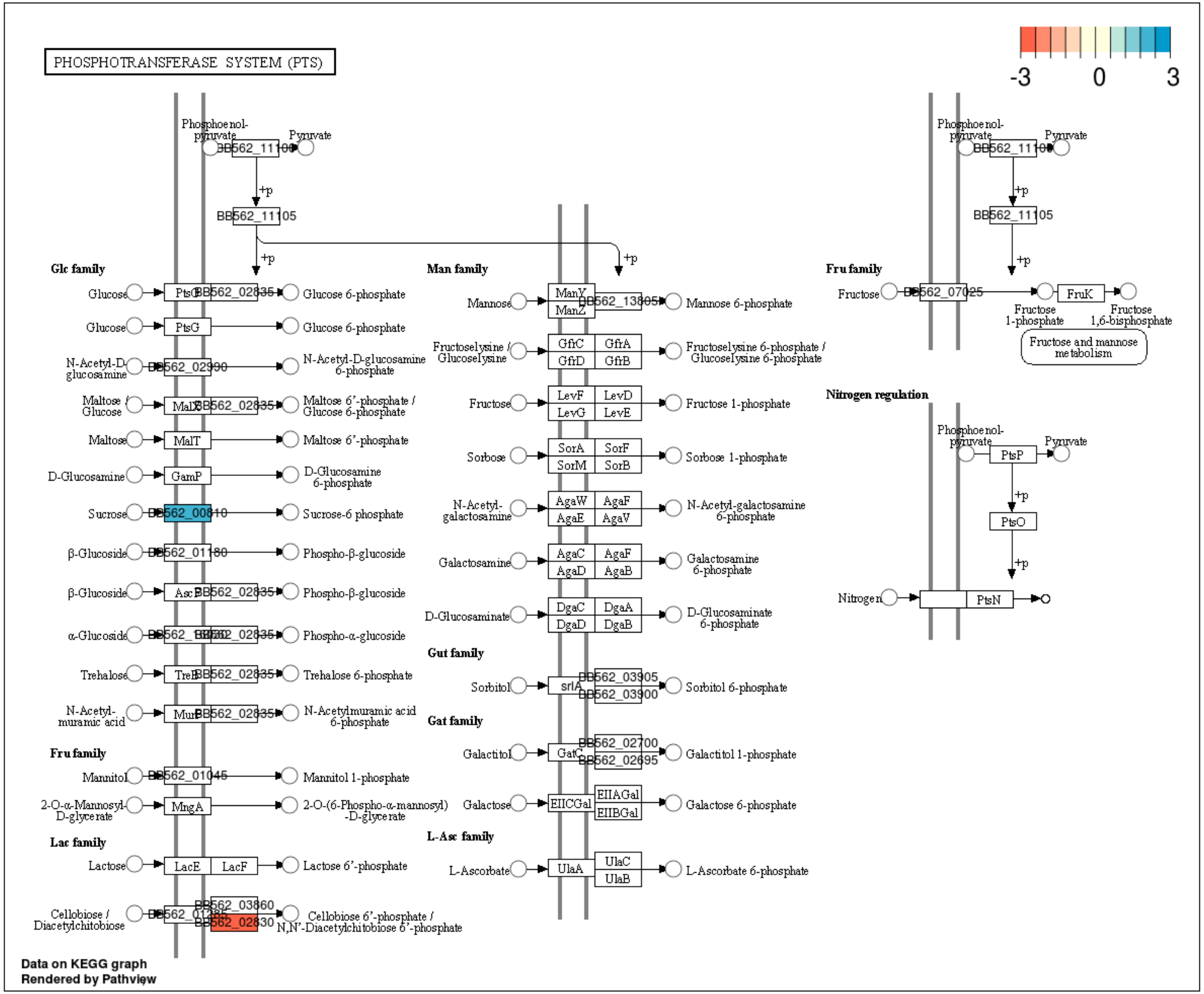
PHOSPHOTRANSFERASE SYSTEM (PTS)
-3
0
3
Phosphoenol-pyruvate
Pyruvate
BB562_11100
+p
BB562_11105
Glc family
Glucose
PtsG
BB562_02835
Glucose 6-phosphate
N-Acetyl-D-glucosamine
BB562_02990
N-Acetyl-D-glucosamine 6-phosphate
Maltose / Glucose
MalX
Maltose 6'-phosphate / Glucose 6-phosphate
Maltose
MalT
Maltose 6'-phosphate
D-Glucosamine
GamP
D-Glucosamine 6-phosphate
Sucrose
BB562_00810
Sucrose-6 phosphate
β-Glucoside
BB562_01180
Phospho-β-glucoside
AscF
α-Glucoside
Phospho-α-glucoside
Trehalose
TreB
Trehalose 6-phosphate
N-Acetyl-muramic acid
MurP
N-Acetylmuramic acid 6-phosphate
Fru family
Mannitol
BB562_01045
Mannitol 1-phosphate
2-O-α-Mannosyl-D-glycerate
MngA
2-O-(6-Phospho-α-mannosyl)-D-glycerate
Lac family
Lactose
LacE
LacF
Lactose 6'-phosphate
Cellobiose / Diacetylchitobiose
BB562_03860
BB562_02830
Cellobiose 6'-phosphate / N,N'-Diacetylchitobiose 6'-phosphate
Man family
Mannose
ManY
ManZ
BB562_13805
Mannose 6-phosphate
Fructoselysine / Glucoselysine
GfrC
GfrD
GfrA
GfrB
Fructoselysine 6-phosphate / Glucoselysine 6-phosphate
Fructose
LevF
LevG
LevD
LevE
Fructose 1-phosphate
Sorbose
SorA
SorM
SorF
SorB
Sorbose 1-phosphate
N-Acetyl-galactosamine
AgaW
AgaE
AgaF
AgaV
N-Acetyl-galactosamine 6-phosphate
Galactosamine
AgaC
AgaD
AgaF
AgaB
Galactosamine 6-phosphate
D-Glucosaminate
DgaC
DgaD
DgaA
DgaB
D-Glucosaminate 6-phosphate
Gut family
Sorbitol
srlA
BB562_03905
BB562_03900
Sorbitol 6-phosphate
Gat family
Galactitol
GatC
BB562_02700
BB562_02695
Galactitol 1-phosphate
Galactose
EIICGal
EIIAGal
EIIBGal
Galactose 6-phosphate
L-Asc family
L-Ascorbate
UlaA
UlaC
UlaB
L-Ascorbate 6-phosphate
Fru family
Fructose
BB562_07025
FruK
Fructose 1-phosphate
Fructose 1,6-bisphosphate
Fructose and mannose metabolism
Nitrogen regulation
PtsP
PtsO
Nitrogen
PtsN
Data on KEGG graph
Rendered by Pathview

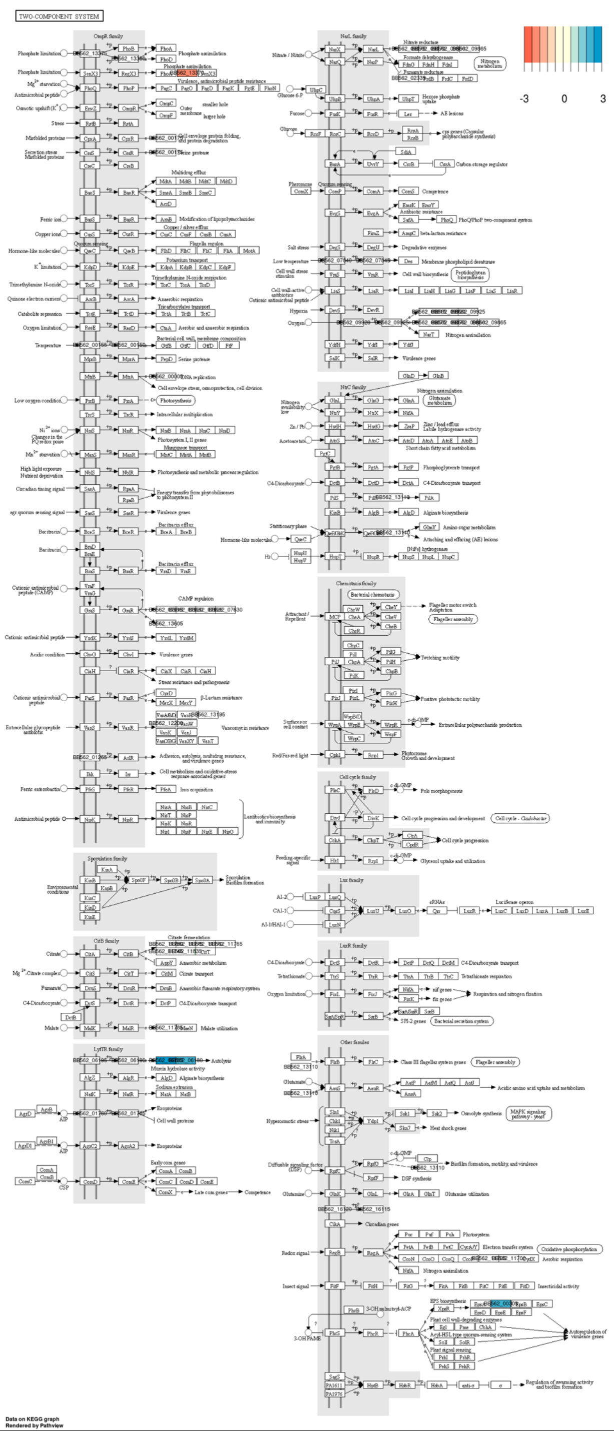
TWO-COMPONENT SYSTEM
-3
0
3
Data on KEGG graph
Rendered by Pathview

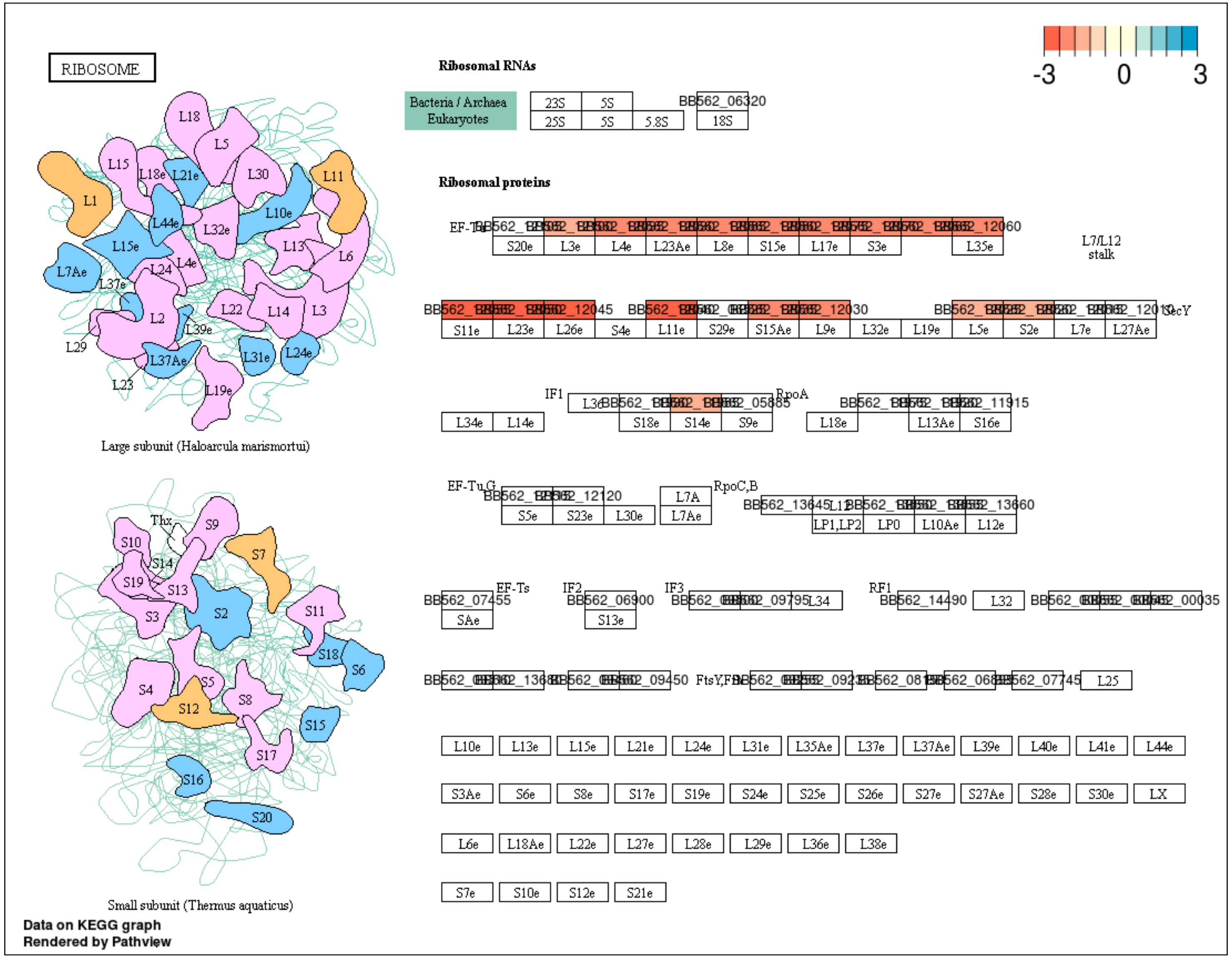

RIBOSOME
Ribosomal RNAs
Bacteria / Archaea
Eukaryotes
23S
5S
25S
5S
5.8S
BB562_06320
18S
Ribosomal proteins
-3
0
3
Large subunit (Haloarcula marismortui)
Small subunit (Thermus aquaticus)
L7/L12
stalk
BB562_12060
BB562_12045
BB562_12030
BB562_05885
BB562_11915
BB562_12120
BB562_13660
BB562_07455
BB562_06900
BB562_14490
BB562_00035
BB562_09450
BB562_07745
Data on KEGG graph
Rendered by Pathview

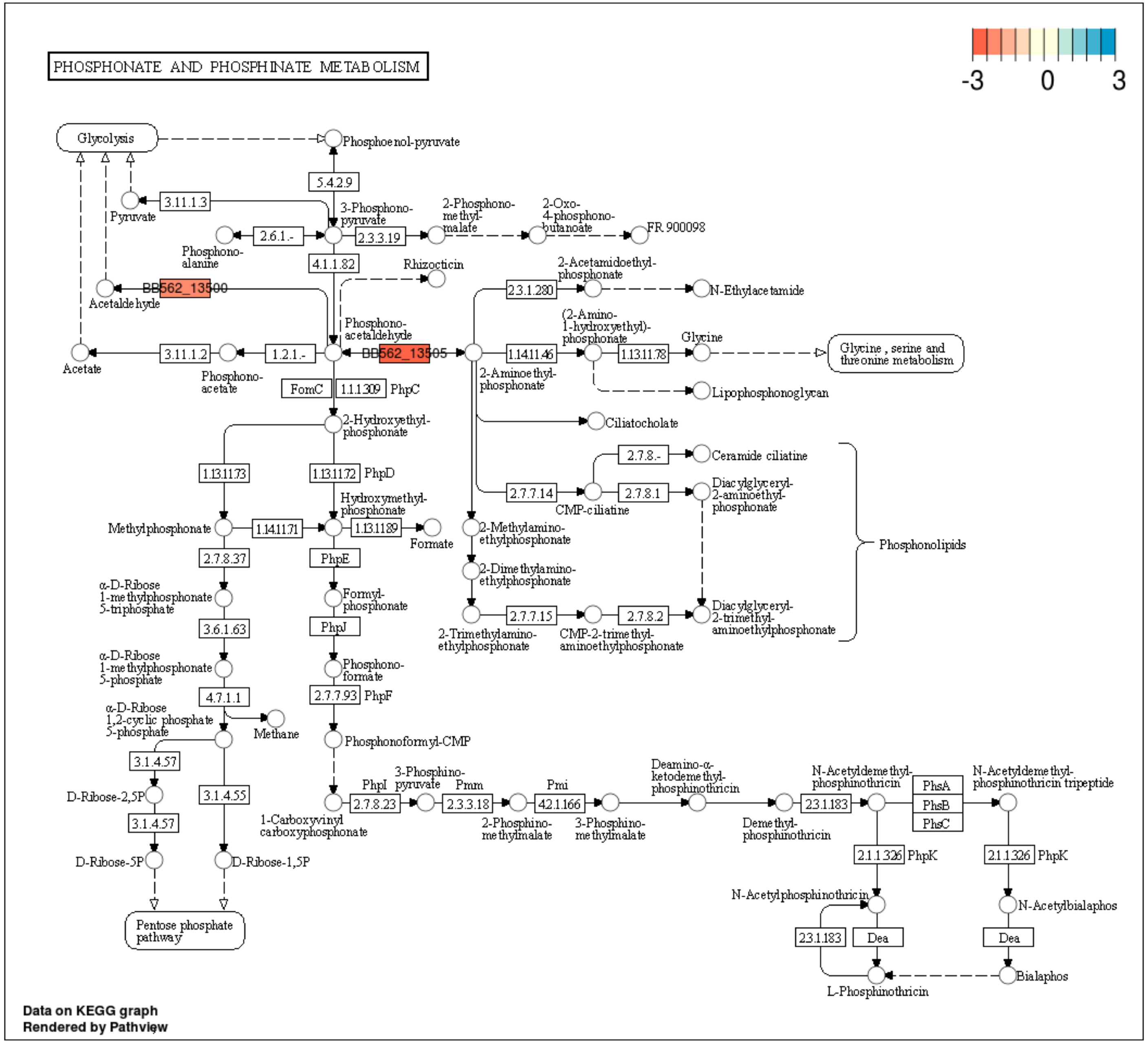

PHOSPHONATE AND PHOSPHINATE METABOLISM
-3 0 3
Glycolysis
Phosphoenol-pyruvate
5.4.2.9
3.11.1.3
Pyruvate
3-Phosphono-pyruvate
2.6.1.-
2.3.3.19
2-Phosphono-methyl-malate
2-Oxo-4-phosphono-butanoate
FR 900098
Phosphono-alanine
4.1.1.82
Rhizocticin
BB562_13500
Acetaldehyde
2-Acetamidoethyl-phosphonate
2.3.1.280
N-Ethylacetamide
Phosphono-acetaldehyde
(2-Amino-1-hydroxyethyl)-phosphonate
Glycine
Glycine, serine and threonine metabolism
Acetate
3.11.1.2
Phosphono-acetate
1.2.1.-
BB562_13505
1.14.11.46
1.13.11.78
2-Aminoethyl-phosphonate
Lipophosphonoglycan
FomC
1.1.1.309
PhpC
2-Hydroxyethyl-phosphonate
Ciliatocholate
2.7.8.-
Ceramide ciliatine
1.13.11.73
1.13.11.72
PhpD
2.7.7.14
2.7.8.1
Diacylglyceryl-2-aminoethyl-phosphonate
CMP-ciliatine
Hydroxymethyl-phosphonate
Methylphosphonate
1.14.11.71
1.13.11.89
Formate
2-Methylamino-ethylphosphonate
Phosphonolipids
2.7.8.37
PhpE
2-Dimethylamino-ethylphosphonate
α-D-Ribose 1-methylphosphonate 5-triphosphate
Formyl-phosphonate
2.7.7.15
2.7.8.2
Diacylglyceryl-2-trimethyl-aminoethylphosphonate
3.6.1.63
PhpJ
2-Trimethylamino-ethylphosphonate
CMP-2-trimethyl-aminoethylphosphonate
α-D-Ribose 1-methylphosphonate 5-phosphate
Phosphono-formate
4.7.1.1
2.7.7.93
PhpF
α-D-Ribose 1,2-cyclic phosphate 5-phosphate
Methane
Phosphonoformyl-CMP
3.1.4.57
Deamino-α-ketodemethyl-phosphinothricin
N-Acetyldemethyl-phosphinothricin
N-Acetyldemethyl-phosphinothricin tripeptide
3-Phosphino-pyruvate
D-Ribose-2,5P
3.1.4.55
PhpI
Pmm
Pmi
PhsA
PhsB
PhsC
2.7.8.23
2.3.3.18
4.2.1.166
2.3.1.183
3.1.4.57
1-Carboxyvinyl carboxyphosphonate
2-Phosphino-methylmalate
3-Phosphino-methylmalate
Demethyl-phosphinothricin
2.1.1.326
PhpK
2.1.1.326
PhpK
D-Ribose-5P
D-Ribose-1,5P
N-Acetylphosphinothricin
N-Acetylbialaphos
2.3.1.183
Dea
Dea
Pentose phosphate pathway
L-Phosphinothricin
Bialaphos
Data on KEGG graph
Rendered by Pathview

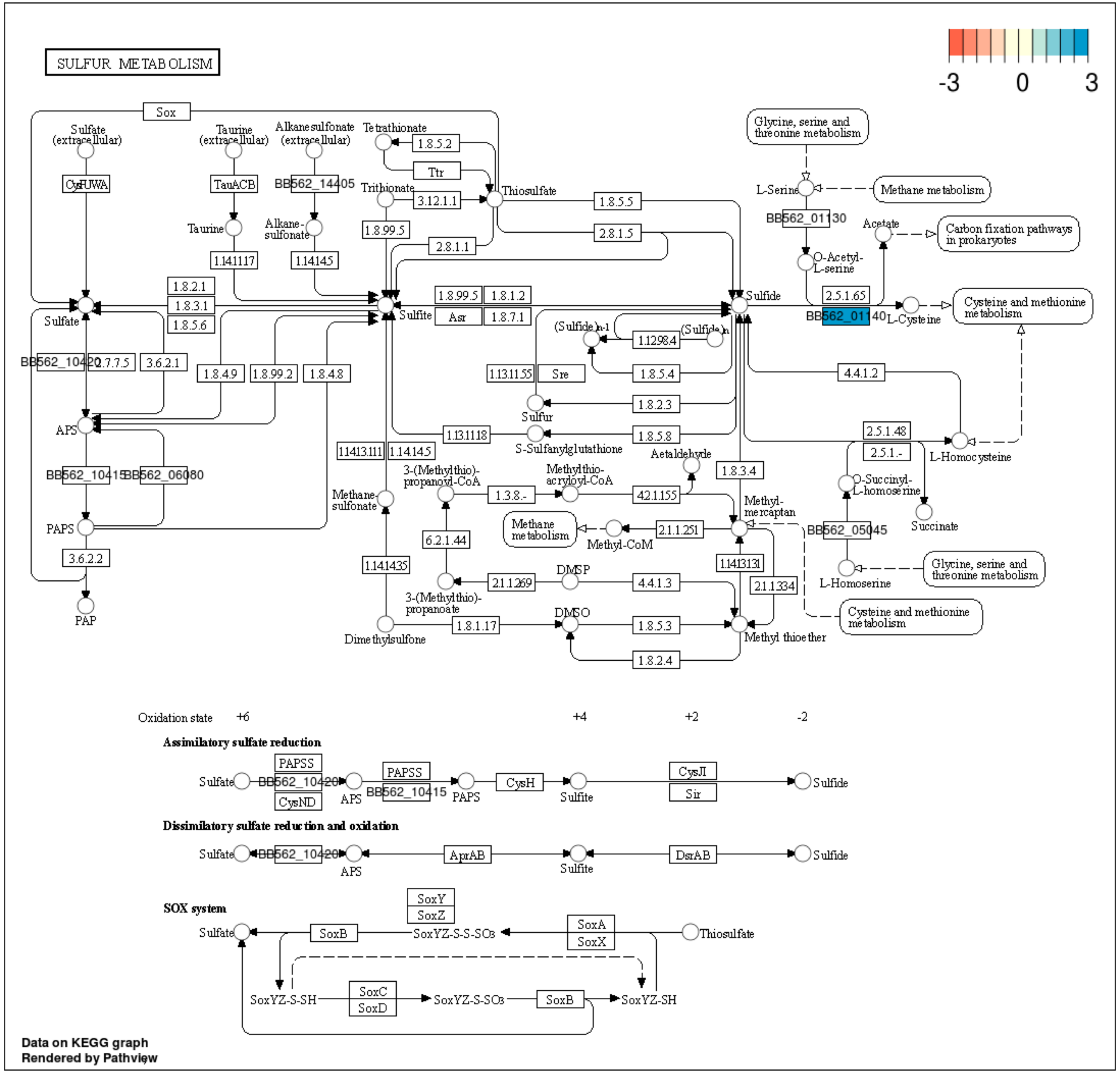
SULFUR METABOLISM
-3
0
3
Sox
Sulfate (extracellular)
CysFUWA
Taurine (extracellular)
TauACB
Alkanesulfonate (extracellular)
BB562_14405
Tetrathionate
1.8.5.2
Ttr
Trithionate
3.12.1.1
Thiosulfate
1.8.5.5
2.8.1.5
Taurine
Alkane-sulfonate
1.8.99.5
2.8.1.1
1.14.11.17
1.14.14.5
1.8.2.1
1.8.3.1
1.8.5.6
Sulfate
Sulfite
1.8.99.5
1.8.1.2
Asr
1.8.7.1
Sulfide
BB562_10420
2.7.7.5
3.6.2.1
1.8.4.9
1.8.99.2
1.8.4.8
APS
BB562_10415
BB562_06080
PAPS
3.6.2.2
PAP
Glycine, serine and threonine metabolism
L-Serine
Methane metabolism
BB562_01130
Acetate
Carbon fixation pathways in prokaryotes
O-Acetyl-L-serine
2.5.1.65
BB562_01140
L-Cysteine
Cysteine and methionine metabolism
(Sulfide)n-1
1.12.98.4
(Sulfide)n
1.13.11.55
Sre
1.8.5.4
4.4.1.2
1.8.2.3
Sulfur
1.13.11.18
1.8.5.8
S-Sulfanylglutathione
2.5.1.48
2.5.1.-
L-Homocysteine
1.14.13.111
1.14.14.5
Aetaldehyde
1.8.3.4
3-(Methylthio)-propanoyl-CoA
Methylthio-acryloyl-CoA
Methane-sulfonate
1.3.8.-
4.2.1.155
Methyl-mercaptan
O-Succinyl-L-homoserine
Succinate
Methane metabolism
Methyl-CoM
2.1.1.251
BB562_05045
6.2.1.44
1.14.14.35
1.14.13.131
DMSP
2.1.1.269
4.4.1.3
2.1.1.334
L-Homoserine
Glycine, serine and threonine metabolism
3-(Methylthio)-propanoate
DMSO
Cysteine and methionine metabolism
1.8.1.17
1.8.5.3
Dimethylsulfone
Methyl thioether
1.8.2.4
Oxidation state
+6
+4
+2
-2
Assimilatory sulfate reduction
Sulfate
PAPSS
BB562_10420
CysND
APS
PAPSS
BB562_10415
PAPS
CysH
Sulfite
CysJI
Sir
Sulfide
Dissimilatory sulfate reduction and oxidation
Sulfate
BB562_10420
APS
AprAB
Sulfite
DsrAB
Sulfide
SOX system
Sulfate
SoxB
SoxY
SoxZ
SoxYZ-S-S-SO3
SoxA
SoxX
Thiosulfate
SoxYZ-S-SH
SoxC
SoxD
SoxYZ-S-SO3
SoxB
SoxYZ-SH
Data on KEGG graph
Rendered by Pathview

Figure S2

Legend:

Figure S2. Overview of the KEGG metabolic pathways regulated in olive-adapted (TO) versus non-adapted (C) *L. pentosus* AP2-16. Metabolic pathways images were generated with the pathview v1.24.0 (Weijun L., 2013) R package and nodes were colored by adding log2FC of its genes for each comparison. The color of DEGs refers to the significance level of differential expressed genes.
