# Supplementary material for: Transcriptomic Profile and Probiotic Properties of Lactiplantibacillus pentosus Pre-adapted to Edible Oils
Source: Front Microbiol. 2021 Oct 14;12:747043. doi: 10.3389/fmicb.2021.747043 (PMC8553220; doi:10.3389/fmicb.2021.747043)
Supplement: Supplementary file 3 [file Data_Sheet_3.PDF]

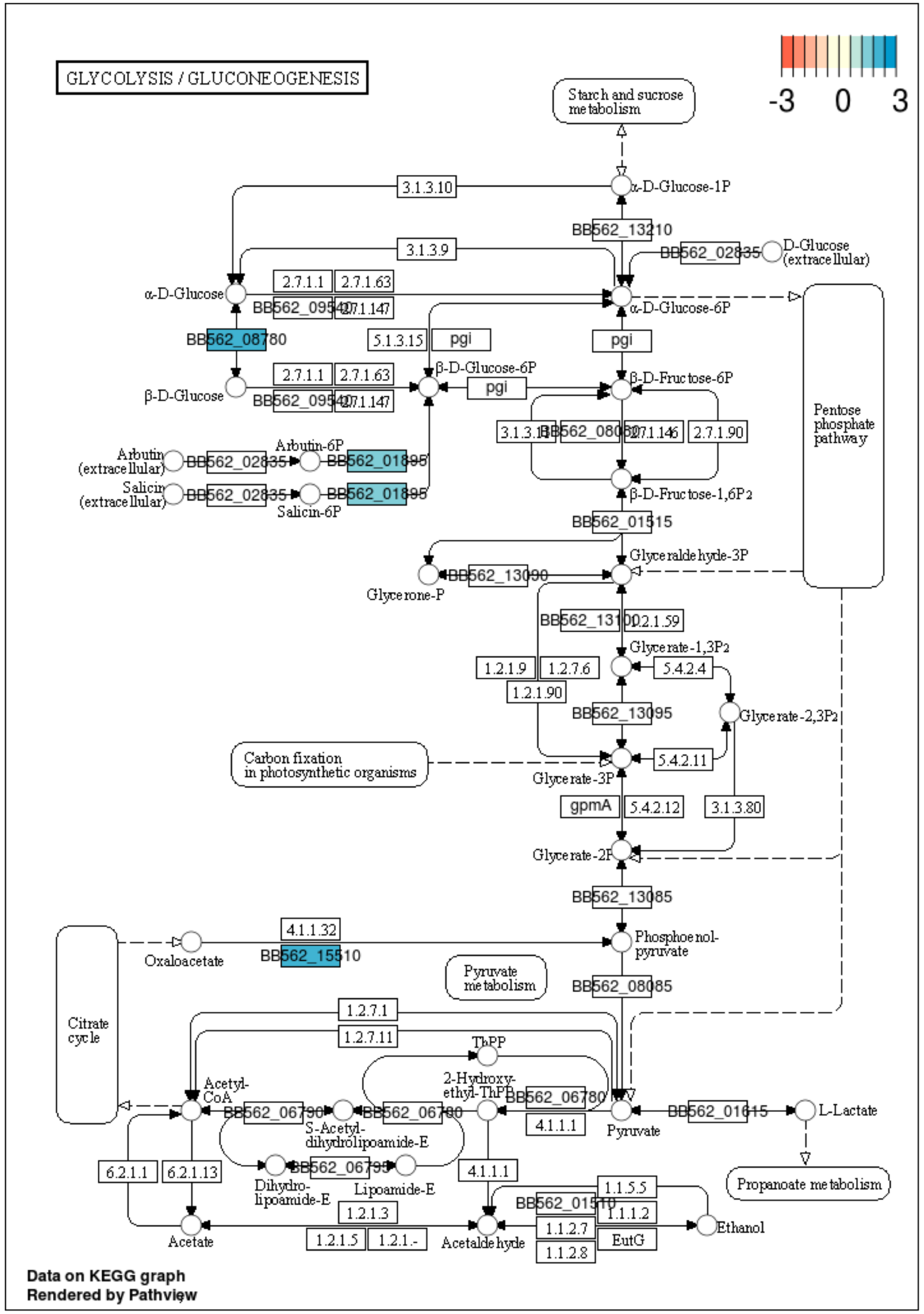
GLYCOLYSIS / GLUCONEOGENESIS
-3 0 3
Starch and sucrose metabolism
α-D-Glucose-1P
3.1.3.10
BB562_13210
3.1.3.9
BB562_02835
D-Glucose (extracellular)
α-D-Glucose
2.7.1.1
2.7.1.63
BB562_09540
2.7.1.147
α-D-Glucose-6P
BB562_08780
5.1.3.15
pgi
β-D-Glucose-6P
β-D-Fructose-6P
β-D-Glucose
Pentose phosphate pathway
3.1.3.11
BB562_08085
2.7.1.146
2.7.1.90
Arbutin (extracellular)
Arbutin-6P
BB562_01895
Salicin (extracellular)
Salicin-6P
β-D-Fructose-1,6P2
BB562_01515
Glyceraldehyde-3P
Glycerone-P
BB562_13090
BB562_13100
1.2.1.59
Glycerate-1,3P2
1.2.1.9
1.2.7.6
5.4.2.4
1.2.1.90
BB562_13095
Glycerate-2,3P2
Carbon fixation in photosynthetic organisms
5.4.2.11
Glycerate-3P
gpmA
5.4.2.12
3.1.3.80
Glycerate-2P
BB562_13085
4.1.1.32
BB562_15510
Oxaloacetate
Phosphoenol-pyruvate
Citrate cycle
Pyruvate metabolism
BB562_08085
1.2.7.1
1.2.7.11
ThPP
Acetyl-CoA
2-Hydroxy-ethyl-ThPP
BB562_06790
BB562_06780
S-Acetyl-dihydrolipoamide-E
4.1.1.1
Pyruvate
BB562_01615
L-Lactate
BB562_06795
Dihydro-lipoamide-E
Lipoamide-E
6.2.1.1
6.2.1.13
4.1.1.1
Propanoate metabolism
1.1.5.5
BB562_01510
1.2.1.3
1.1.1.2
Ethanol
1.2.1.5
1.2.1.-
1.1.2.7
EutG
Acetate
Acetaldehyde
1.1.2.8
Data on KEGG graph
Rendered by Pathview

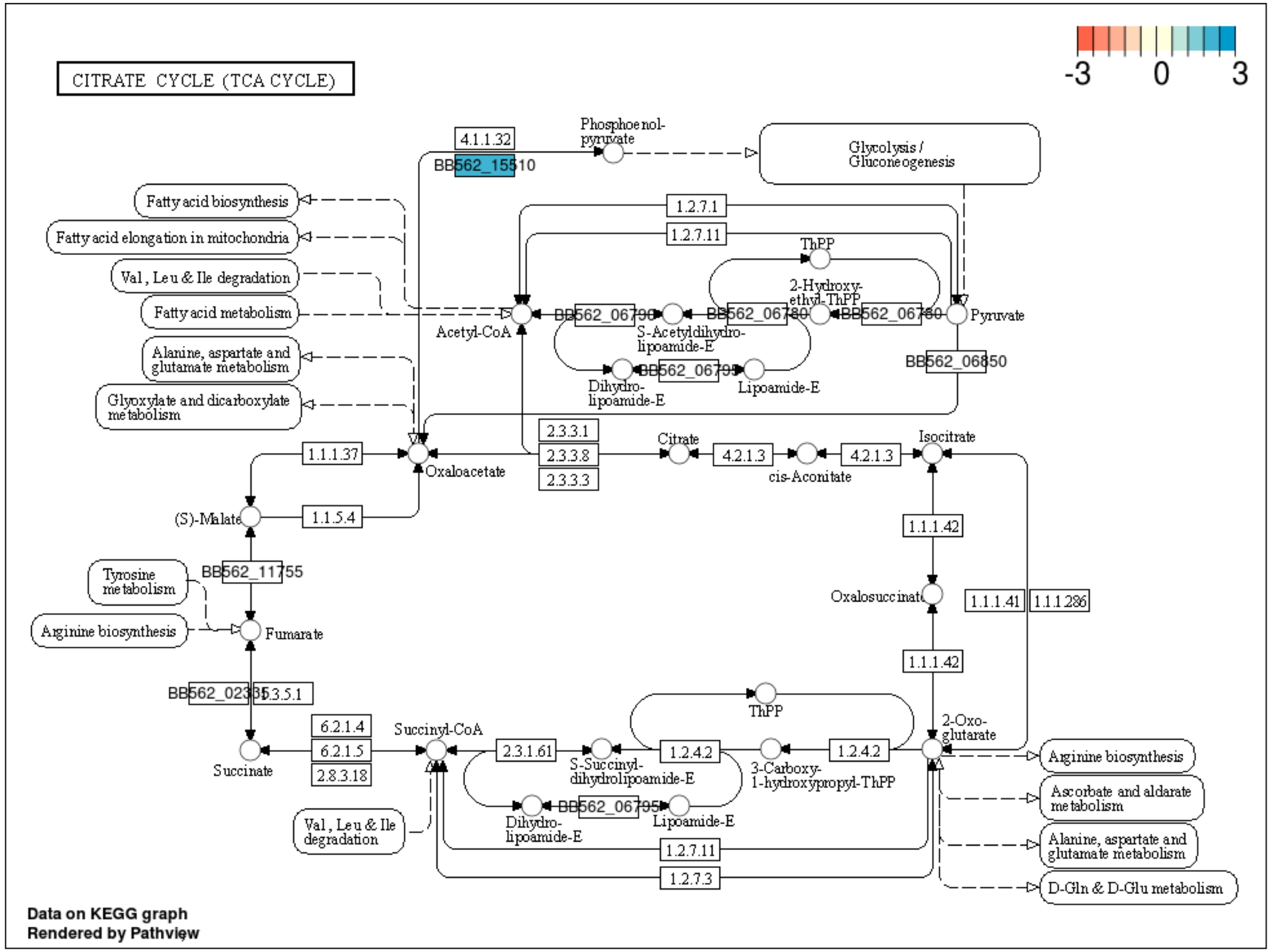
CITRATE CYCLE (TCA CYCLE)
-3 0 3
4.1.1.32
BB562_15510
Phosphoenol-pyruvate
Glycolysis / Gluconeogenesis
Fatty acid biosynthesis
Fatty acid elongation in mitochondria
Val, Leu & Ile degradation
Fatty acid metabolism
1.2.7.1
1.2.7.11
ThPP
2-Hydroxy-ethyl-ThPP
BB562_06790
BB562_06780
BB562_06780
Pyruvate
Acetyl-CoA
S-Acetyldihydro-lipoamide-E
BB562_06850
Alanine, aspartate and glutamate metabolism
BB562_06795
Dihydro-lipoamide-E
Lipoamide-E
Glyoxylate and dicarboxylate metabolism
2.3.3.1
2.3.3.8
2.3.3.3
Citrate
4.2.1.3
cis-Aconitate
4.2.1.3
Isocitrate
1.1.1.37
Oxaloacetate
(S)-Malate
1.1.5.4
1.1.1.42
BB562_11755
Tyrosine metabolism
Oxalosuccinate
1.1.1.41
1.1.1.286
Arginine biosynthesis
Fumarate
1.1.1.42
BB562_02335
1.3.5.1
ThPP
6.2.1.4
6.2.1.5
2.8.3.18
Succinyl-CoA
2.3.1.61
S-Succinyl-dihydrolipoamide-E
1.2.4.2
3-Carboxy-1-hydroxypropyl-ThPP
1.2.4.2
2-Oxo-glutarate
Succinate
Arginine biosynthesis
Val, Leu & Ile degradation
BB562_06795
Dihydro-lipoamide-E
Lipoamide-E
Ascorbate and aldarate metabolism
Alanine, aspartate and glutamate metabolism
1.2.7.11
1.2.7.3
D-Gln & D-Glu metabolism
Data on KEGG graph
Rendered by Pathview

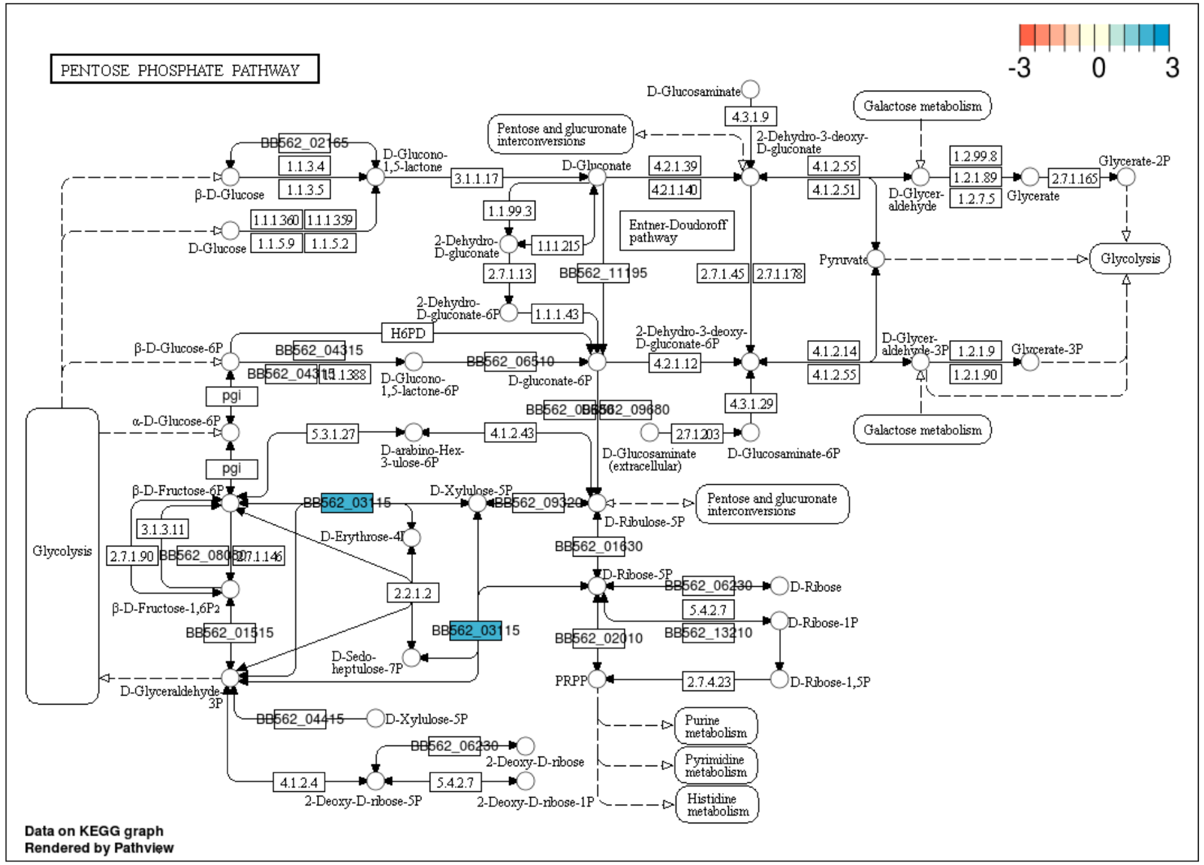
PENTOSE PHOSPHATE PATHWAY
-3
0
3
D-Glucosaminate
Pentose and glucuronate interconversions
Galactose metabolism
β-D-Glucose
D-Glucono-1,5-lactone
D-Gluconate
2-Dehydro-3-deoxy-D-gluconate
D-Glyceraldehyde
Glycerate
Glycerate-2P
D-Glucose
2-Dehydro-D-gluconate
Entner-Doudoroff pathway
Pyruvate
Glycolysis
2-Dehydro-D-gluconate-6P
H6PD
β-D-Glucose-6P
D-Glucono-1,5-lactone-6P
D-gluconate-6P
2-Dehydro-3-deoxy-D-gluconate-6P
D-Glyceraldehyde-3P
Glycerate-3P
pgi
α-D-Glucose-6P
D-arabino-Hex-3-ulose-6P
D-Glucosaminate (extracellular)
D-Glucosaminate-6P
β-D-Fructose-6P
D-Xylulose-5P
D-Ribulose-5P
D-Erythrose-4P
β-D-Fructose-1,6P2
D-Ribose-5P
D-Ribose
D-Ribose-1P
D-Sedo-heptulose-7P
PRPP
D-Ribose-1,5P
D-Glyceraldehyde-3P
D-Xylulose-5P
2-Deoxy-D-ribose
2-Deoxy-D-ribose-5P
2-Deoxy-D-ribose-1P
Purine metabolism
Pyrimidine metabolism
Histidine metabolism
Data on KEGG graph
Rendered by Pathview

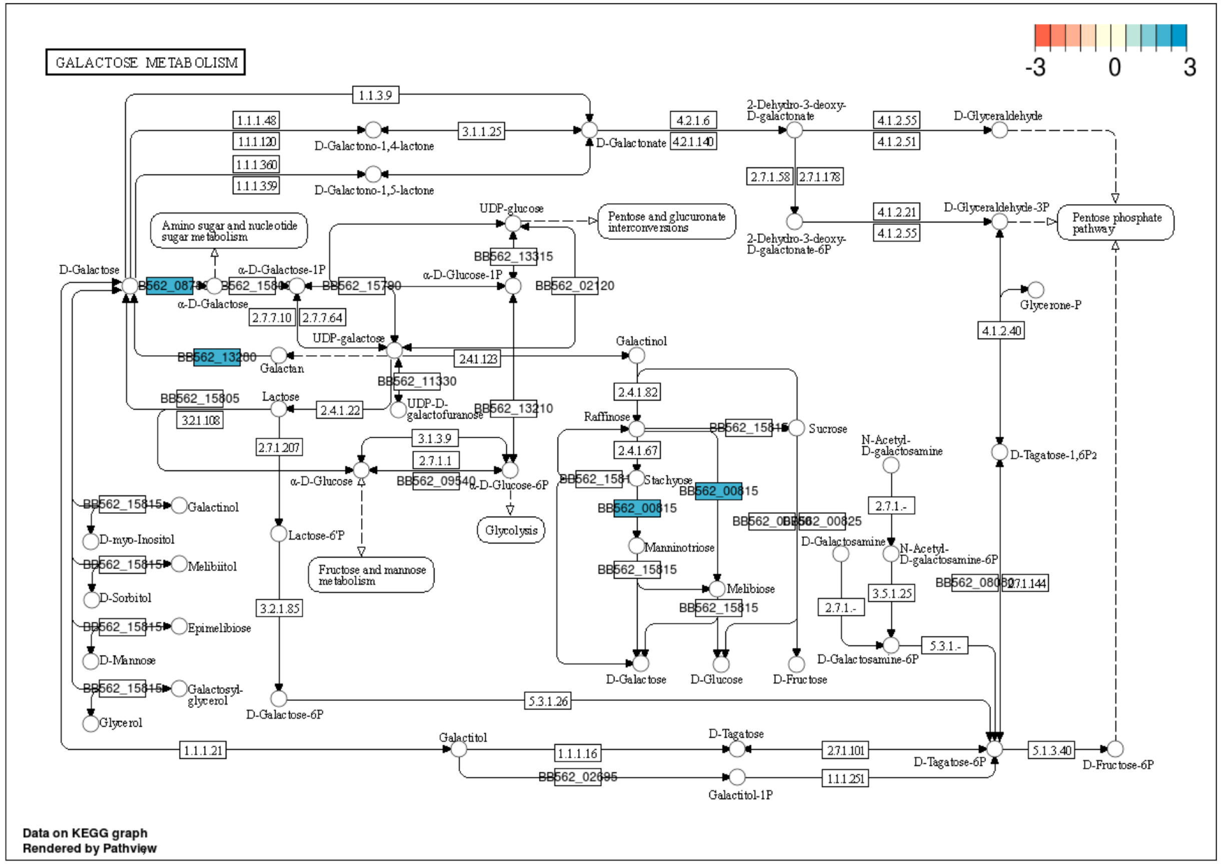
GALACTOSE METABOLISM
-3
0
3
D-Galactono-1,4-lactone
D-Galactonate
2-Dehydro-3-deoxy-D-galactonate
D-Glyceraldehyde
D-Galactono-1,5-lactone
Amino sugar and nucleotide sugar metabolism
UDP-glucose
Pentose and glucuronate interconversions
2-Dehydro-3-deoxy-D-galactonate-6P
D-Glyceraldehyde-3P
Pentose phosphate pathway
D-Galactose
α-D-Galactose-1P
α-D-Glucose-1P
α-D-Galactose
UDP-galactose
Glycerone-P
Galactan
Galactinol
Lactose
UDP-D-galactofuranose
Raffinose
Sucrose
N-Acetyl-D-galactosamine
α-D-Glucose
α-D-Glucose-6P
Stachyose
D-Tagatose-1,6P2
Galactinol
Lactose-6P
Glycolysis
D-myo-Inositol
Mannintriose
D-Galactosamine
N-Acetyl-D-galactosamine-6P
Melibiitol
Fructose and mannose metabolism
Melibiose
D-Sorbitol
Epimelibiose
D-Mannose
D-Galactose
D-Glucose
D-Fructose
D-Galactosamine-6P
Galactosylglycerol
D-Galactose-6P
Glycerol
Galactitol
D-Tagatose
D-Tagatose-6P
D-Fructose-6P
Galactitol-1P
Data on KEGG graph
Rendered by Pathview

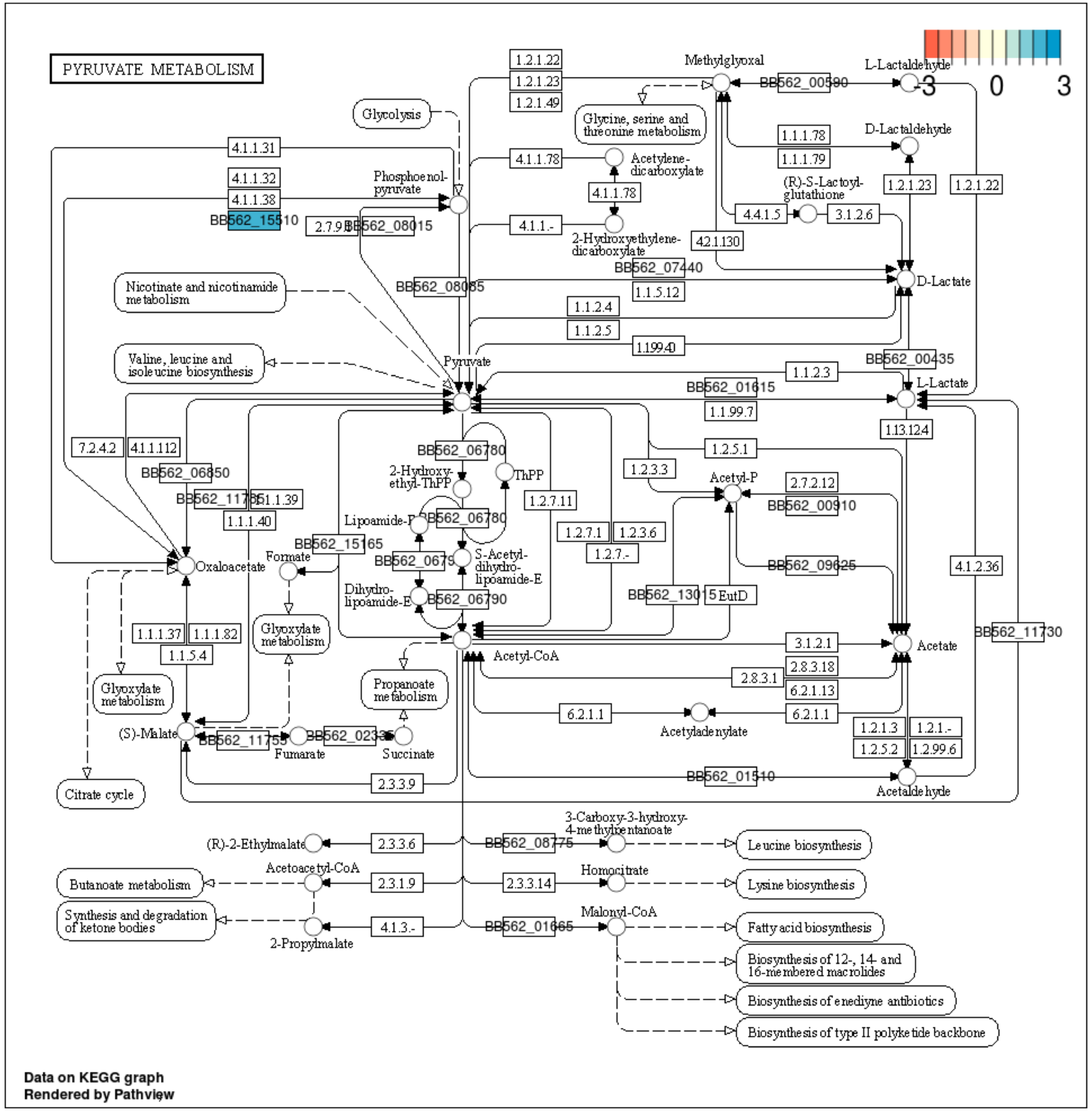
PYRUVATE METABOLISM
-3
0
3
Methylglyoxal
L-Lactaldehyde
D-Lactaldehyde
Glycolysis
Glycine, serine and threonine metabolism
Acetylene-dicarboxylate
2-Hydroxyethylene-dicarboxylate
(R)-S-Lactoyl-glutathione
Phosphoenol-pyruvate
Nicotinate and nicotinamide metabolism
Valine, leucine and isoleucine biosynthesis
Pyruvate
D-Lactate
L-Lactate
2-Hydroxy-ethyl-ThPP
ThPP
Lipoamide-E
S-Acetyl-dihydro-lipoamide-E
Dihydro-lipoamide-E
Acetyl-P
Oxaloacetate
Formate
Glyoxylate metabolism
Propanoate metabolism
Acetyl-CoA
Acetate
Acetyladenylate
Acetaldehyde
(S)-Malate
Fumarate
Succinate
Citrate cycle
(R)-2-Ethylmalate
3-Carboxy-3-hydroxy-4-methylpentanoate
Leucine biosynthesis
Acetoacetyl-CoA
Homocitrate
Lysine biosynthesis
Butanoate metabolism
Synthesis and degradation of ketone bodies
2-Propylmalate
Malonyl-CoA
Fatty acid biosynthesis
Biosynthesis of 12-, 14- and 16-membered macrolides
Biosynthesis of enediyne antibiotics
Biosynthesis of type II polyketide backbone
BB562_00590
BB562_15510
BB562_08015
BB562_07440
BB562_08065
BB562_00435
BB562_01615
BB562_06780
BB562_06850
BB562_15165
BB562_06790
BB562_00910
BB562_09625
BB562_13015
BB562_11730
BB562_01510
BB562_08775
BB562_01665
Data on KEGG graph
Rendered by Pathview

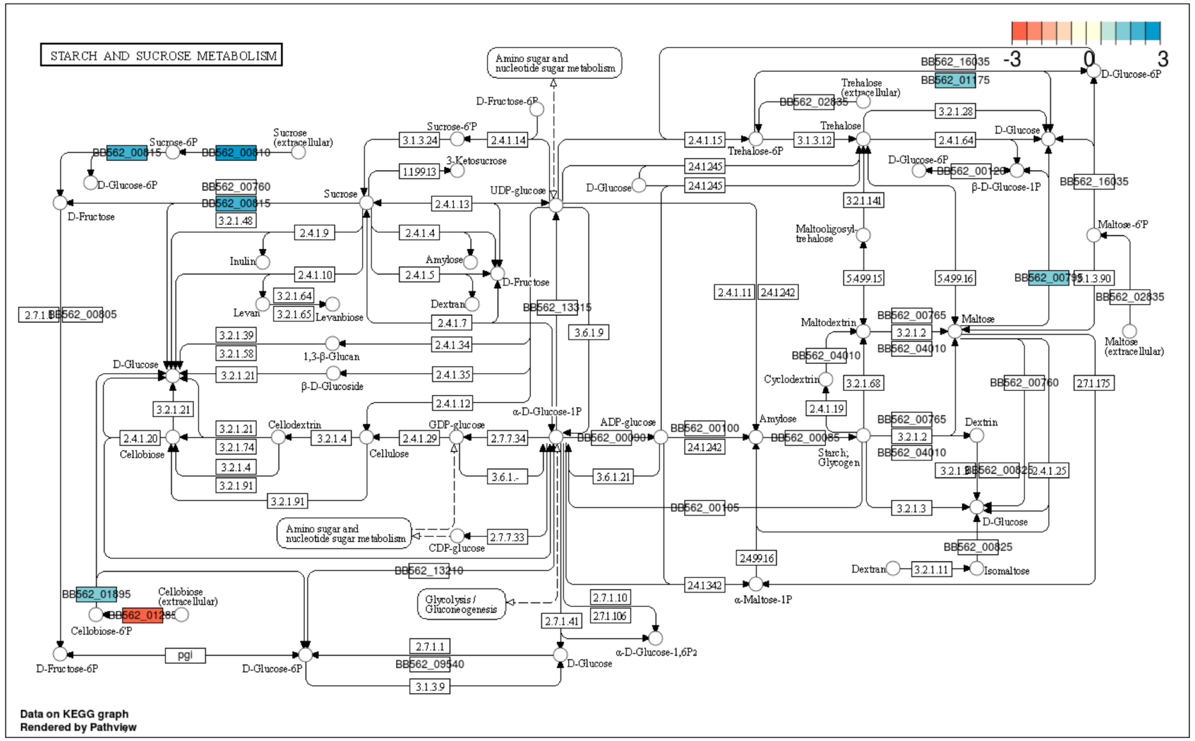
STARCH AND SUCROSE METABOLISM
-3
0
3
Amino sugar and nucleotide sugar metabolism
D-Fructose-6P
Sucrose-6P
Sucrose (extracellular)
D-Glucose-6P
D-Fructose
Sucrose
3-Ketosucrose
UDP-glucose
Inulin
Levan
Levanbiose
Amylose
Dextran
1,3-β-Glucan
β-D-Glucoside
D-Glucose
Cellodextrin
Cellobiose
Cellulose
GDP-glucose
α-D-Glucose-1P
ADP-glucose
CDP-glucose
Glycolysis / Gluconeogenesis
Cellobiose (extracellular)
Cellobiose-6P
D-Fructose-6P
D-Glucose-6P
D-Glucose
α-D-Glucose-1,6P2
Trehalose (extracellular)
Trehalose
Trehalose-6P
D-Glucose-6P
β-D-Glucose-1P
Maltooligosyl-trehalose
Maltose-6P
Maltodextrin
Maltose
Maltose (extracellular)
Cyclodextrin
Amylose
Starch; Glycogen
Dextrin
Isomaltose
α-Maltose-1P
BB562_00815
BB562_00810
BB562_00760
BB562_16035
BB562_01175
BB562_02835
BB562_13315
BB562_00765
BB562_04010
BB562_00100
BB562_00085
BB562_00105
BB562_00825
BB562_13210
BB562_01895
BB562_09540
Data on KEGG graph
Rendered by Pathview

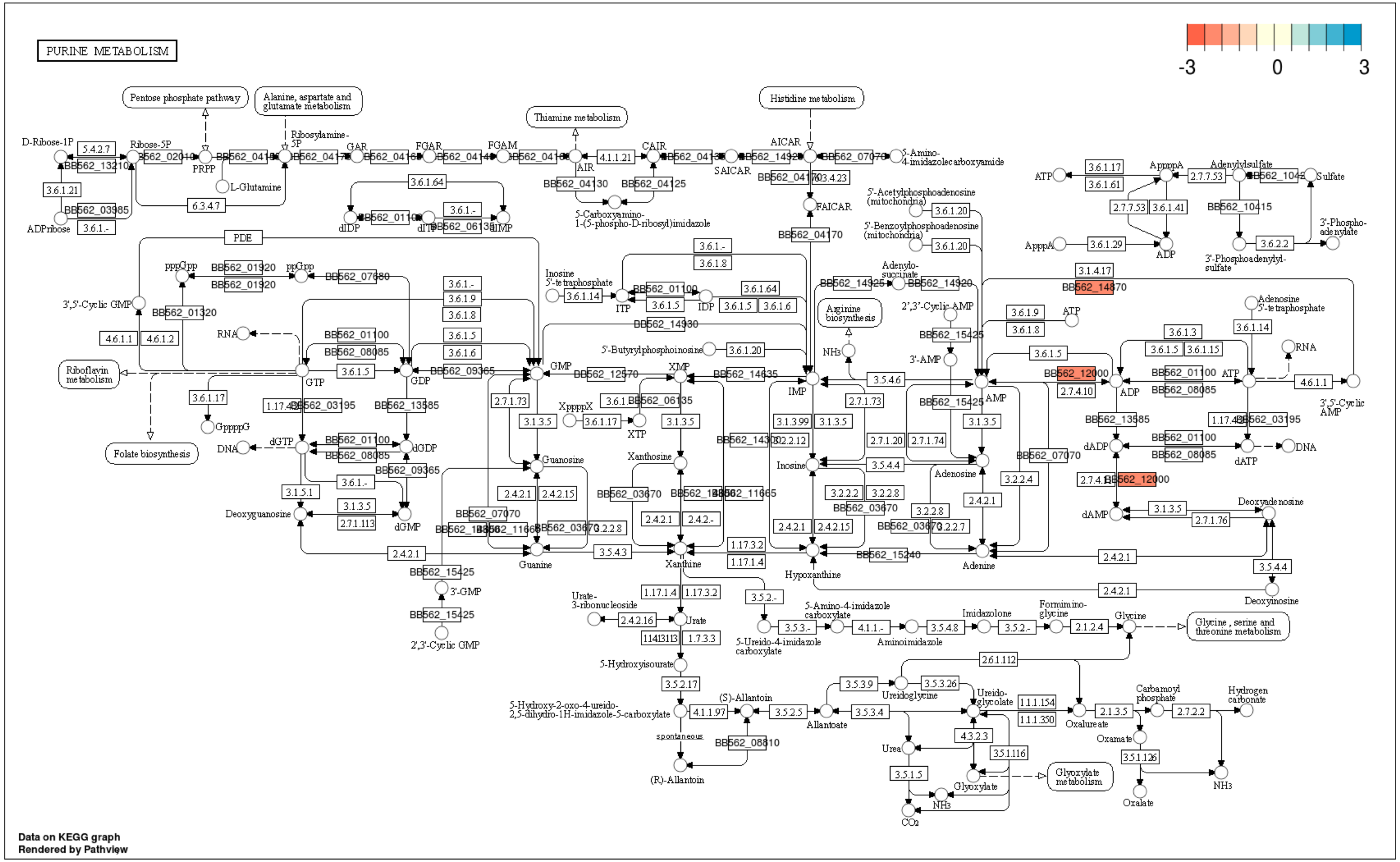
PURINE METABOLISM
-3
0
3
Pentose phosphate pathway
Alanine, aspartate and glutamate metabolism
Thiamine metabolism
Histidine metabolism
D-Ribose-1P
Ribose-5P
PRPP
L-Glutamine
Ribosylamine-5P
GAR
FGAR
FGAM
AIR
CAIR
SAICAR
AICAR
FAICAR
5-Amino-4-imidazolecarboxyamide
5-Carboxyamino-1-(5-phospho-D-ribosyl)imidazole
ADPribose
dIDP
dITP
dIMP
PDE
pppGpp
ppGpp
3',5'-Cyclic GMP
RNA
Riboflavin metabolism
Folate biosynthesis
GTP
GDP
GMP
GppppG
DNA
dGTP
dGDP
dGMP
Deoxyguanosine
Guanosine
Guanine
Inosine 5'-tetraphosphate
ITP
IDP
IMP
XTP
XMP
XppppX
Xanthosine
Xanthine
5'-Butyrylphosphoinosine
3'-GMP
2',3'-Cyclic GMP
Urate-3-ribonucleoside
Urate
5-Hydroxyisourate
5-Hydroxy-2-oxo-4-ureido-2,5-dihydro-1H-imidazole-5-carboxylate
(S)-Allantoin
(R)-Allantoin
spontaneous
Allantoate
Ureidoglycine
Ureidoglycolate
Urea
Glyoxylate
Glyoxylate metabolism
NH3
CO2
Oxalureate
Oxamate
Oxalate
Carbamoyl phosphate
Hydrogen carbonate
Inosine
Hypoxanthine
Arginine biosynthesis
5'-Acetylphosphoadenosine (mitochondria)
5'-Benzoylphosphoadenosine (mitochondria)
Adenylo-succinate
2',3'-Cyclic AMP
3'-AMP
AMP
ADP
ATP
Adenosine
Adenine
dADP
dAMP
dATP
Deoxyadenosine
Deoxyinosine
3',5'-Cyclic AMP
Adenosine 5'-tetraphosphate
ApppA
ApppA
Adenylylsulfate
Sulfate
3'-Phosphoadenylylsulfate
3'-Phosphoadenylate
5-Ureido-4-imidazole carboxylate
5-Amino-4-imidazole carboxylate
Aminoimidazole
Imidazolone
Formimino-glycine
Glycine
Glycine, serine and threonine metabolism
BB562_14870
BB562_12000
Data on KEGG graph
Rendered by Pathview

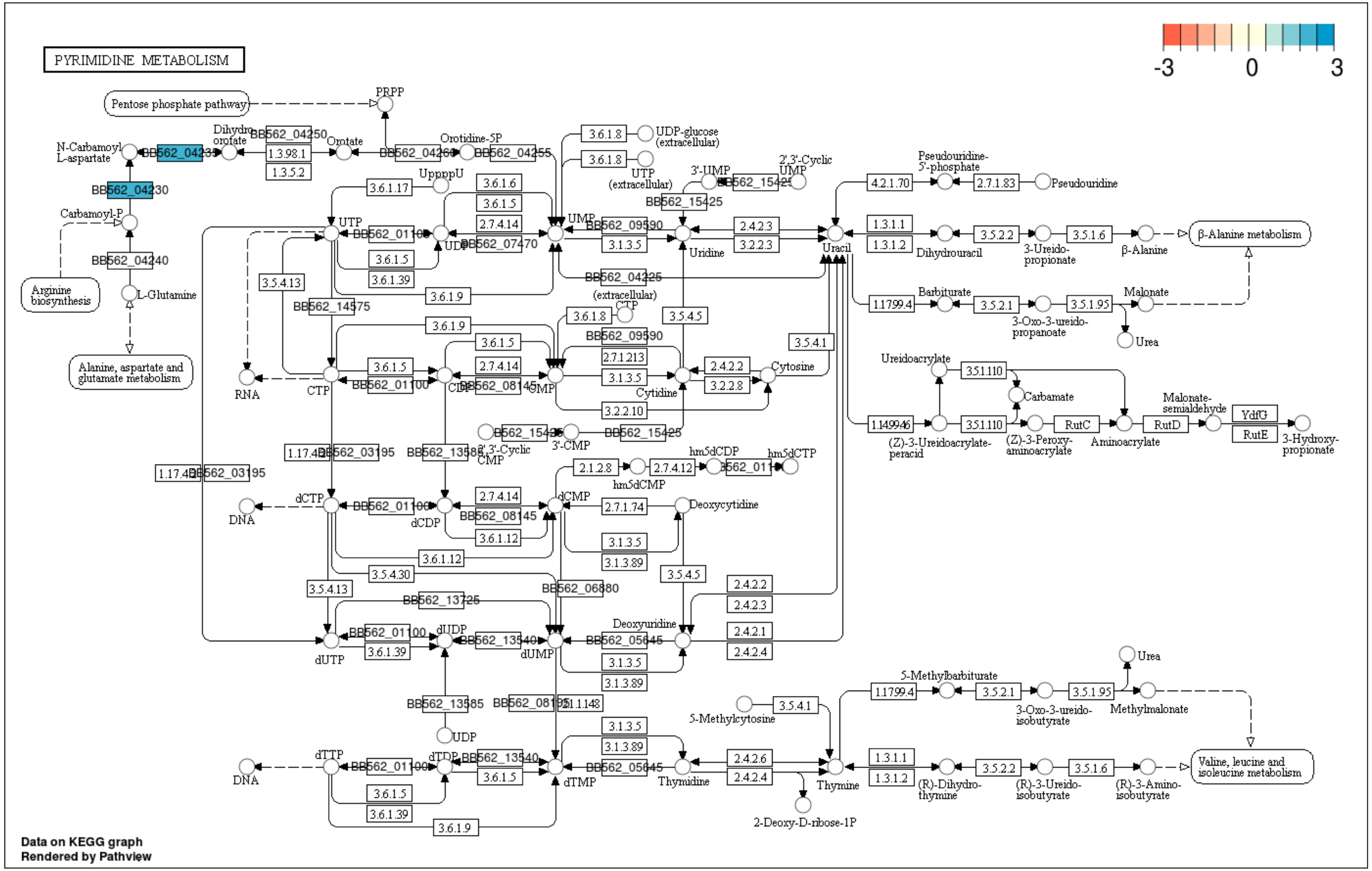
PYRIMIDINE METABOLISM
-3
0
3
Pentose phosphate pathway
PRPP
N-Carbamoyl-L-aspartate
Dihydro-orotate
Orotate
Orotidine-5P
BB562_04235
BB562_04250
1.3.98.1
1.3.5.2
BB562_04260
BB562_04255
BB562_04230
Carbamoyl-P
BB562_04240
L-Glutamine
Arginine biosynthesis
Alanine, aspartate and glutamate metabolism
UDP-glucose (extracellular)
UTP (extracellular)
3.6.1.8
UppppU
3.6.1.17
3.6.1.6
3.6.1.5
2.7.4.14
UTP
UDP
UMP
BB562_01100
BB562_07470
3.6.1.5
3.6.1.39
3.6.1.9
3.5.4.13
BB562_14575
3'-UMP
2',3'-Cyclic UMP
BB562_15425
BB562_09590
3.1.3.5
Uridine
2.4.2.3
3.2.2.3
Uracil
BB562_04225
CTP (extracellular)
3.5.4.5
3.5.4.1
BB562_09590
2.7.1.213
3.1.3.5
2.4.2.2
3.2.2.8
Cytosine
Cytidine
3.2.2.10
RNA
CTP
CDP
CMP
BB562_08145
3.6.1.9
BB562_15425
2',3'-Cyclic CMP
3'-CMP
1.17.4.1
BB562_03195
BB562_13585
2.1.2.8
hm5dCMP
2.7.4.12
hm5dCDP
hm5dCTP
DNA
dCTP
dCDP
dCMP
2.7.4.14
2.7.1.74
Deoxycytidine
3.6.1.12
3.1.3.5
3.1.3.89
3.5.4.30
3.5.4.13
BB562_13725
BB562_06880
3.5.4.5
2.4.2.2
2.4.2.3
2.4.2.1
2.4.2.4
Deoxyuridine
dUTP
dUDP
dUMP
BB562_13540
BB562_05645
3.6.1.39
3.1.3.5
3.1.3.89
BB562_13585
BB562_08195
2.1.1.148
UDP
5-Methylcytosine
3.5.4.1
DNA
dTTP
dTDP
dTMP
Thymidine
2.4.2.6
2.4.2.4
Thymine
2-Deoxy-D-ribose-1P
3.6.1.5
3.6.1.39
3.6.1.9
Pseudouridine-5'-phosphate
4.2.1.70
2.7.1.83
Pseudouridine
1.3.1.1
1.3.1.2
Dihydrouracil
3.5.2.2
3-Ureido-propionate
3.5.1.6
β-Alanine
β-Alanine metabolism
Barbiturate
1.17.99.4
3.5.2.1
3-Oxo-3-ureido-propanoate
3.5.1.95
Malonate
Urea
Ureidoacrylate
3.5.1.110
Carbamate
1.14.99.46
(Z)-3-Ureidoacrylate-peracid
(Z)-3-Peroxy-aminoacrylate
RutC
Aminoacrylate
RutD
Malonate-semialdehyde
YdfG
RutE
3-Hydroxy-propionate
5-Methylbarbiturate
1.17.99.4
3.5.2.1
3-Oxo-3-ureido-isobutyrate
3.5.1.95
Methylmalonate
Urea
Valine, leucine and isoleucine metabolism
1.3.1.1
1.3.1.2
(R)-Dihydro-thymine
3.5.2.2
(R)-3-Ureido-isobutyrate
3.5.1.6
(R)-3-Amino-isobutyrate
Data on KEGG graph
Rendered by Pathview

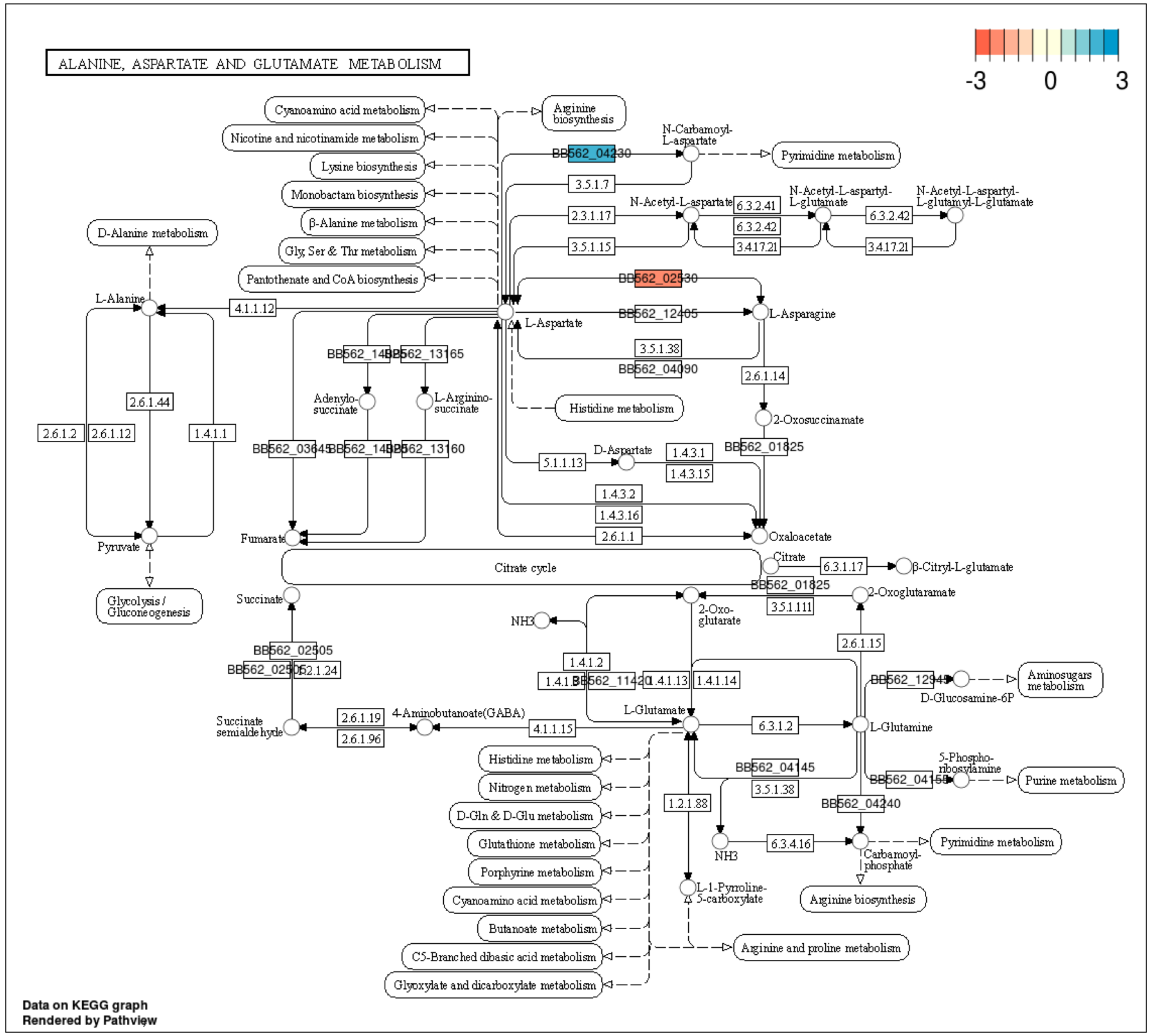
ALANINE, ASPARTATE AND GLUTAMATE METABOLISM
-3
0
3
Cyanoamino acid metabolism
Nicotine and nicotinamide metabolism
Lysine biosynthesis
Monobactam biosynthesis
β-Alanine metabolism
Gly, Ser & Thr metabolism
Pantothenate and CoA biosynthesis
Arginine biosynthesis
N-Carbamoyl-L-aspartate
Pyrimidine metabolism
BB562_04230
N-Acetyl-L-aspartate
N-Acetyl-L-aspartyl-L-glutamate
N-Acetyl-L-aspartyl-L-glutamyl-L-glutamate
D-Alanine metabolism
L-Alanine
L-Aspartate
L-Asparagine
BB562_02530
BB562_12405
BB562_04090
Adenylo-succinate
L-Arginino-succinate
Histidine metabolism
2-Oxosuccinamate
D-Aspartate
BB562_01825
Pyruvate
Fumarate
Oxaloacetate
Citrate
Citrate cycle
β-Citryl-L-glutamate
Glycolysis / Gluconeogenesis
Succinate
2-Oxoglutaramate
2-Oxo-glutarate
NH3
BB562_02505
4-Aminobutanoate(GABA)
Succinate semialdehyde
L-Glutamate
L-Glutamine
Aminosugars metabolism
D-Glucosamine-6P
5-Phospho-ribosylamine
Purine metabolism
Histidine metabolism
Nitrogen metabolism
D-Gln & D-Glu metabolism
Glutathione metabolism
Porphyrine metabolism
Cyanoamino acid metabolism
Butanoate metabolism
C5-Branched dibasic acid metabolism
Glyoxylate and dicarboxylate metabolism
Carbamoyl-phosphate
Pyrimidine metabolism
Arginine biosynthesis
L-1-Pyrroline-5-carboxylate
Arginine and proline metabolism
Data on KEGG graph
Rendered by Pathview

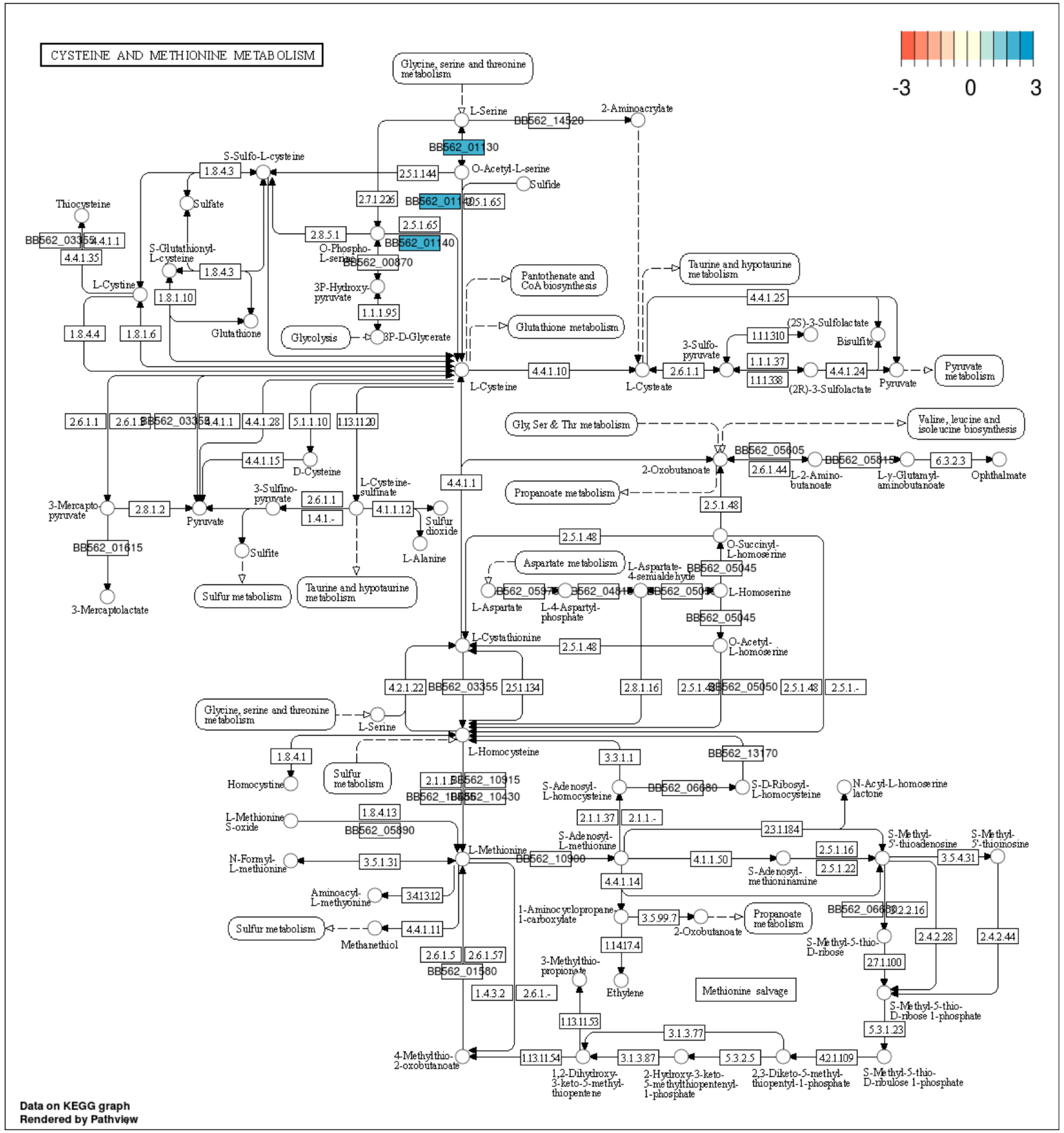
CYSTEINE AND METHIONINE METABOLISM
-3
0
3
Glycine, serine and threonine metabolism
L-Serine
2-Aminoacrylate
BB562_01130
O-Acetyl-L-serine
Sulfide
S-Sulfo-L-cysteine
Sulfate
Thiocysteine
S-Glutathionyl-L-cysteine
L-Cystine
Glutathione
O-Phospho-L-serine
BB562_01140
BB562_00870
3P-Hydroxy-pyruvate
Glycolysis
3P-D-Glycerate
Pantothenate and CoA biosynthesis
Glutathione metabolism
Taurine and hypotaurine metabolism
(2S)-3-Sulfolactate
Bisulfite
3-Sulfo-pyruvate
L-Cysteine
L-Cysteate
(2R)-3-Sulfolactate
Pyruvate
Pyruvate metabolism
Gly, Ser & Thr metabolism
Valine, leucine and isoleucine biosynthesis
2-Oxobutanoate
L-2-Amino-butanoate
L-γ-Glutamyl-aminobutanoate
Ophthalmate
Propanoate metabolism
3-Mercapto-pyruvate
3-Sulfino-pyruvate
L-Cysteine-sulfinate
Sulfur dioxide
Sulfite
L-Alanine
3-Mercaptolactate
Sulfur metabolism
Taurine and hypotaurine metabolism
Aspartate metabolism
L-Aspartate
L-4-Aspartyl-phosphate
L-Aspartate-4-semialdehyde
O-Succinyl-L-homoserine
L-Homoserine
O-Acetyl-L-homoserine
L-Cystathionine
Glycine, serine and threonine metabolism
L-Serine
L-Homocysteine
Homocysteine
Sulfur metabolism
S-Adenosyl-L-homocysteine
S-D-Ribosyl-L-homocysteine
N-Acyl-L-homoserine lactone
L-Methionine S-oxide
L-Methionine
S-Adenosyl-L-methionine
S-Methyl-5'-thioadenosine
S-Methyl-5'-thioinosine
N-Formyl-L-methionine
S-Adenosyl-methioninamine
Aminoacyl-L-methionine
1-Aminocyclopropane-1-carboxylate
2-Oxobutanoate
Propanoate metabolism
Sulfur metabolism
Methanethiol
S-Methyl-5-thio-D-ribose
3-Methylthio-propanoate
Ethylene
Methionine salvage
S-Methyl-5-thio-D-ribose 1-phosphate
4-Methylthio-2-oxobutanoate
1,2-Dihydroxy-3-keto-5-methylthiopentene
2-Hydroxy-3-keto-5-methylthiopentenyl-1-phosphate
2,3-Diketo-5-methylthiopentyl-1-phosphate
S-Methyl-5-thio-D-ribulose 1-phosphate
Data on KEGG graph
Rendered by Pathview

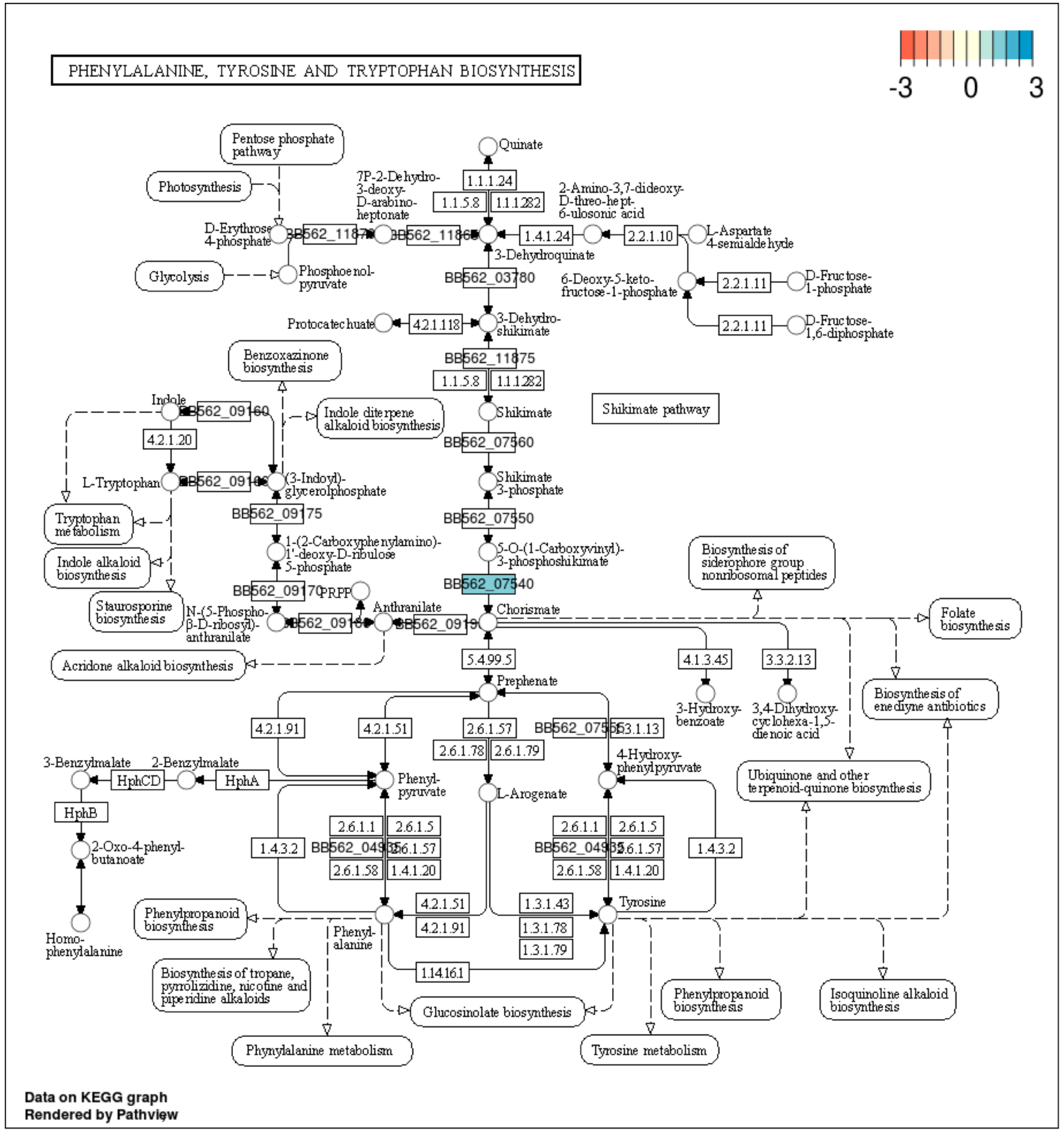
PHENYLALANINE, TYROSINE AND TRYPTOPHAN BIOSYNTHESIS
-3
0
3
Pentose phosphate pathway
Photosynthesis
Glycolysis
D-Erythrose 4-phosphate
Phosphoenol-pyruvate
7P-2-Dehydro-3-deoxy-D-arabino-heptonate
Quinate
1.1.1.24
1.1.5.8
1.1.1.282
2-Amino-3,7-dideoxy-D-threo-hept-6-ulosonic acid
1.4.1.24
2.2.1.10
L-Aspartate 4-semialdehyde
3-Dehydroquinate
BB562_03780
6-Deoxy-5-keto-fructose-1-phosphate
2.2.1.11
D-Fructose-1-phosphate
D-Fructose-1,6-diphosphate
Protocatechuate
4.2.1.118
3-Dehydro-shikimate
BB562_11875
Benzoxazinone biosynthesis
Indole
Indole diterpene alkaloid biosynthesis
Shikimate
Shikimate pathway
BB562_07560
4.2.1.20
L-Tryptophan
(3-Indoyl)-glycerolphosphate
Shikimate 3-phosphate
BB562_07550
Tryptophan metabolism
BB562_09175
1-(2-Carboxyphenylamino)-1'-deoxy-D-ribulose 5-phosphate
5-O-(1-Carboxyvinyl)-3-phosphoshikimate
Biosynthesis of siderophore group nonribosomal peptides
Indole alkaloid biosynthesis
BB562_09170
PRPP
BB562_07540
Staurosporine biosynthesis
N-(5-Phospho-β-D-ribosyl)-anthranilate
Anthranilate
Chorismate
Folate biosynthesis
Acridone alkaloid biosynthesis
5.4.99.5
4.1.3.45
3.3.2.13
Biosynthesis of enediyne antibiotics
Prephenate
3-Hydroxy-benzoate
3,4-Dihydroxy-cyclohexa-1,5-dienoic acid
4.2.1.91
4.2.1.51
2.6.1.57
2.6.1.78
2.6.1.79
4-Hydroxy-phenylpyruvate
3-Benzylmalate
2-Benzylmalate
HphCD
HphA
Phenyl-pyruvate
L-Arogenate
Ubiquinone and other terpenoid-quinone biosynthesis
HphB
2-Oxo-4-phenyl-butanoate
2.6.1.1
2.6.1.5
2.6.1.58
1.4.1.20
1.4.3.2
Homo-phenylalanine
Phenylpropanoid biosynthesis
1.3.1.43
1.3.1.78
1.3.1.79
Tyrosine
Phenyl-alanine
1.14.16.1
Biosynthesis of tropane, pyrrolizidine, nicotine and piperidine alkaloids
Glucosinolate biosynthesis
Isoquinoline alkaloid biosynthesis
Phynylalanine metabolism
Tyrosine metabolism
Data on KEGG graph
Rendered by Pathview

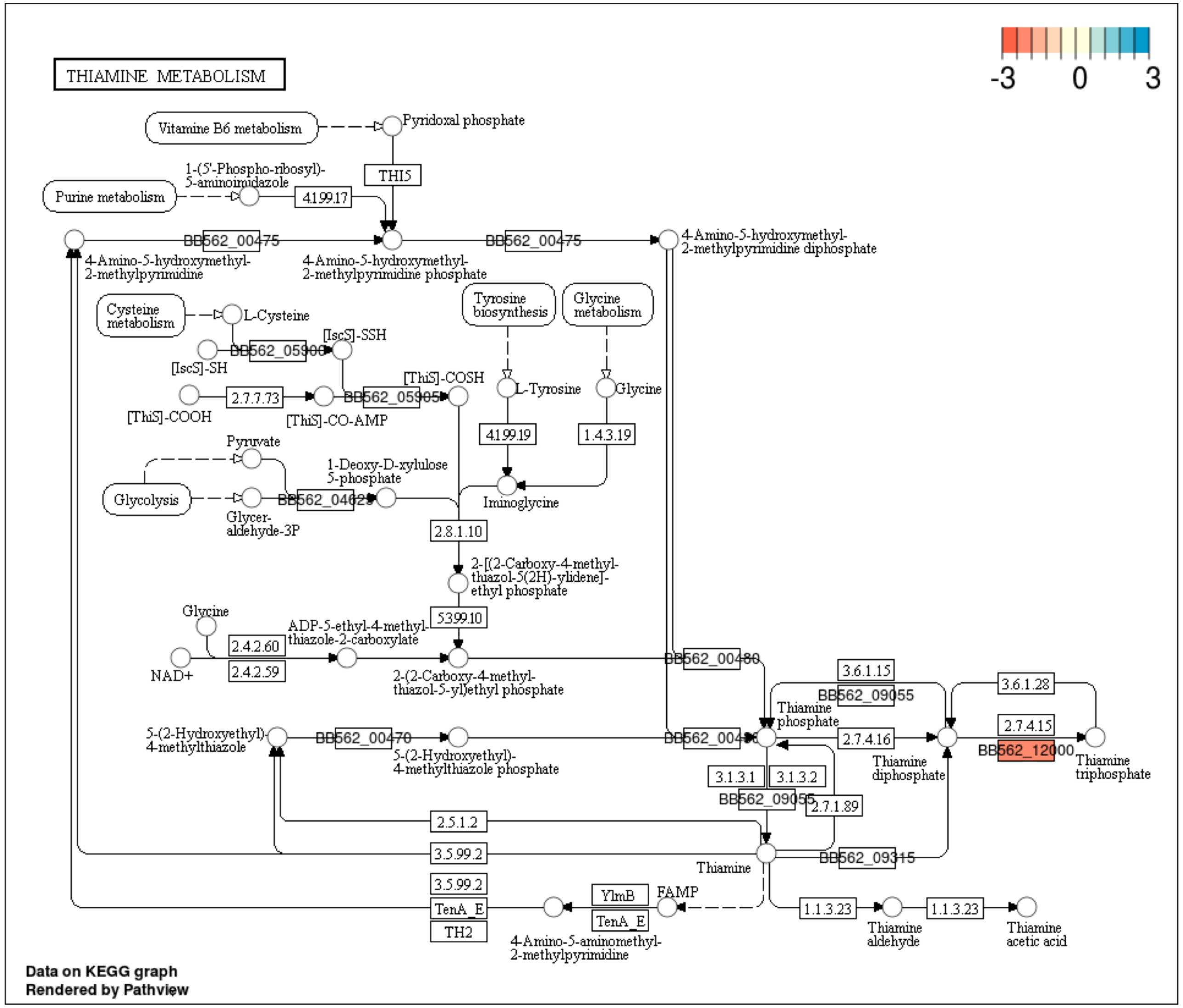
THIAMINE METABOLISM
-3
0
3
Vitamine B6 metabolism
Pyridoxal phosphate
THI5
Purine metabolism
1-(5'-Phospho-ribosyl)-5-aminoimidazole
4.1.99.17
BB562_00475
4-Amino-5-hydroxymethyl-2-methylpyrimidine
4-Amino-5-hydroxymethyl-2-methylpyrimidine phosphate
4-Amino-5-hydroxymethyl-2-methylpyrimidine diphosphate
Cysteine metabolism
L-Cysteine
[IscS]-SSH
[IscS]-SH
Tyrosine biosynthesis
Glycine metabolism
[ThiS]-COSH
[ThiS]-COOH
2.7.7.73
[ThiS]-CO-AMP
L-Tyrosine
Glycine
4.1.99.19
1.4.3.19
Pyruvate
1-Deoxy-D-xylulose 5-phosphate
Glycolysis
Glycer-aldehyde-3P
Iminoglycine
2.8.1.10
2-[(2-Carboxy-4-methyl-thiazol-5(2H)-ylidene]-ethyl phosphate
5.3.99.10
Glycine
ADP-5-ethyl-4-methyl-thiazole-2-carboxylate
2.4.2.60
2.4.2.59
NAD+
2-(2-Carboxy-4-methyl-thiazol-5-yl)ethyl phosphate
3.6.1.15
3.6.1.28
BB562_09055
Thiamine phosphate
2.7.4.16
2.7.4.15
BB562_12000
5-(2-Hydroxyethyl)-4-methylthiazole
5-(2-Hydroxyethyl)-4-methylthiazole phosphate
Thiamine diphosphate
Thiamine triphosphate
3.1.3.1
3.1.3.2
2.7.1.89
2.5.1.2
3.5.99.2
Thiamine
BB562_09315
YlmB
TenA_E
TH2
FAMP
1.1.3.23
4-Amino-5-aminomethyl-2-methylpyrimidine
Thiamine aldehyde
Thiamine acetic acid
Data on KEGG graph
Rendered by Pathview

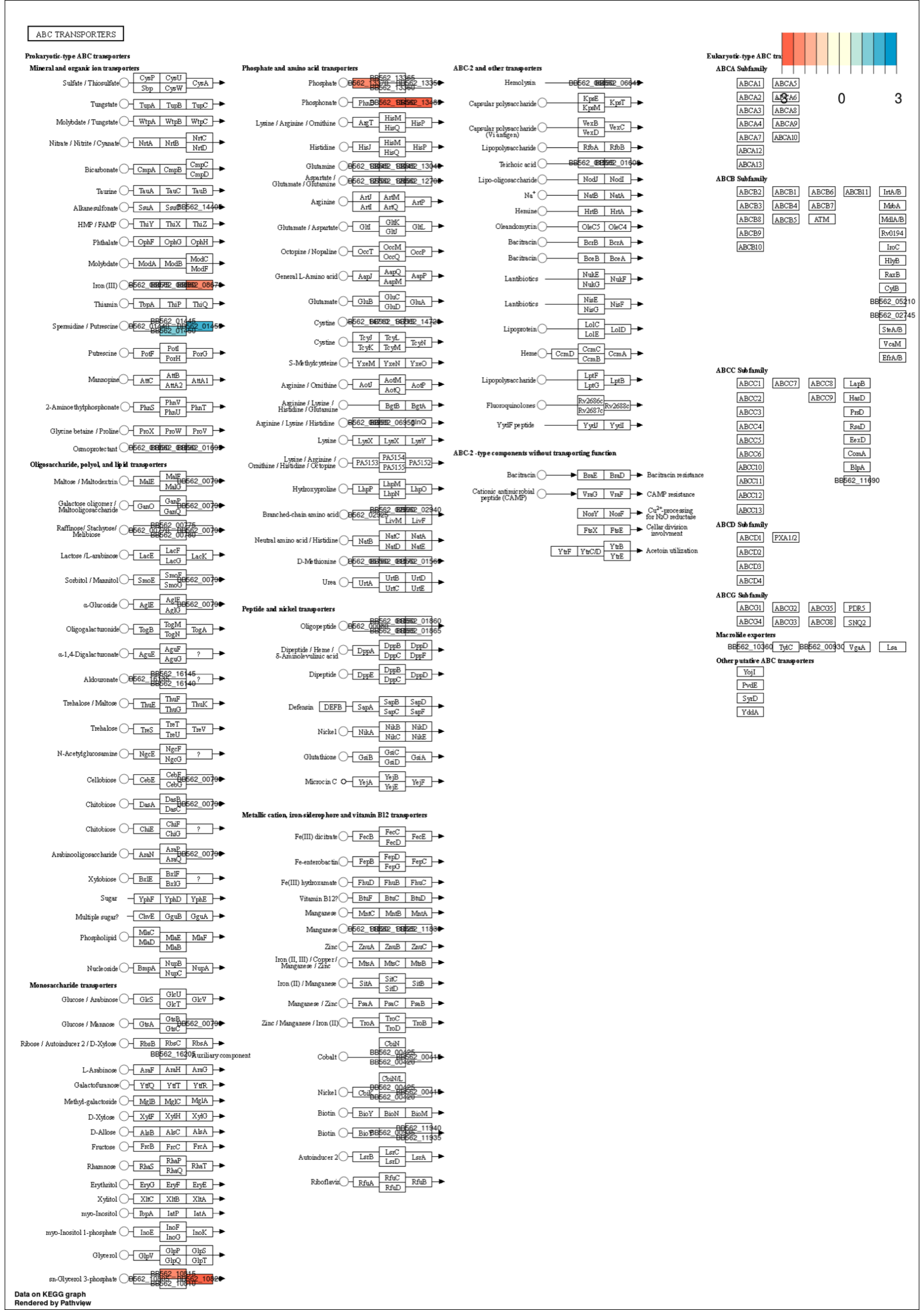
ABC TRANSPORTERS
Prokaryotic-type ABC transporters
Mineral and organic ion transporters
Phosphate and amino acid transporters
ABC-2 and other transporters
Eukaryotic-type ABC tr
ABCA Subfamily
ABCB Subfamily
ABCC Subfamily
ABCD Subfamily
ABCG Subfamily
Macrolide exporters
Other putative ABC transporters
Oligosaccharide, polyol, and lipid transporters
Monosaccharide transporters
Peptide and nickel transporters
Metallic cation, iron-siderophore and vitamin B12 transporters
ABC-2 -type components without transporting function
0
3
Data on KEGG graph
Rendered by Pathview

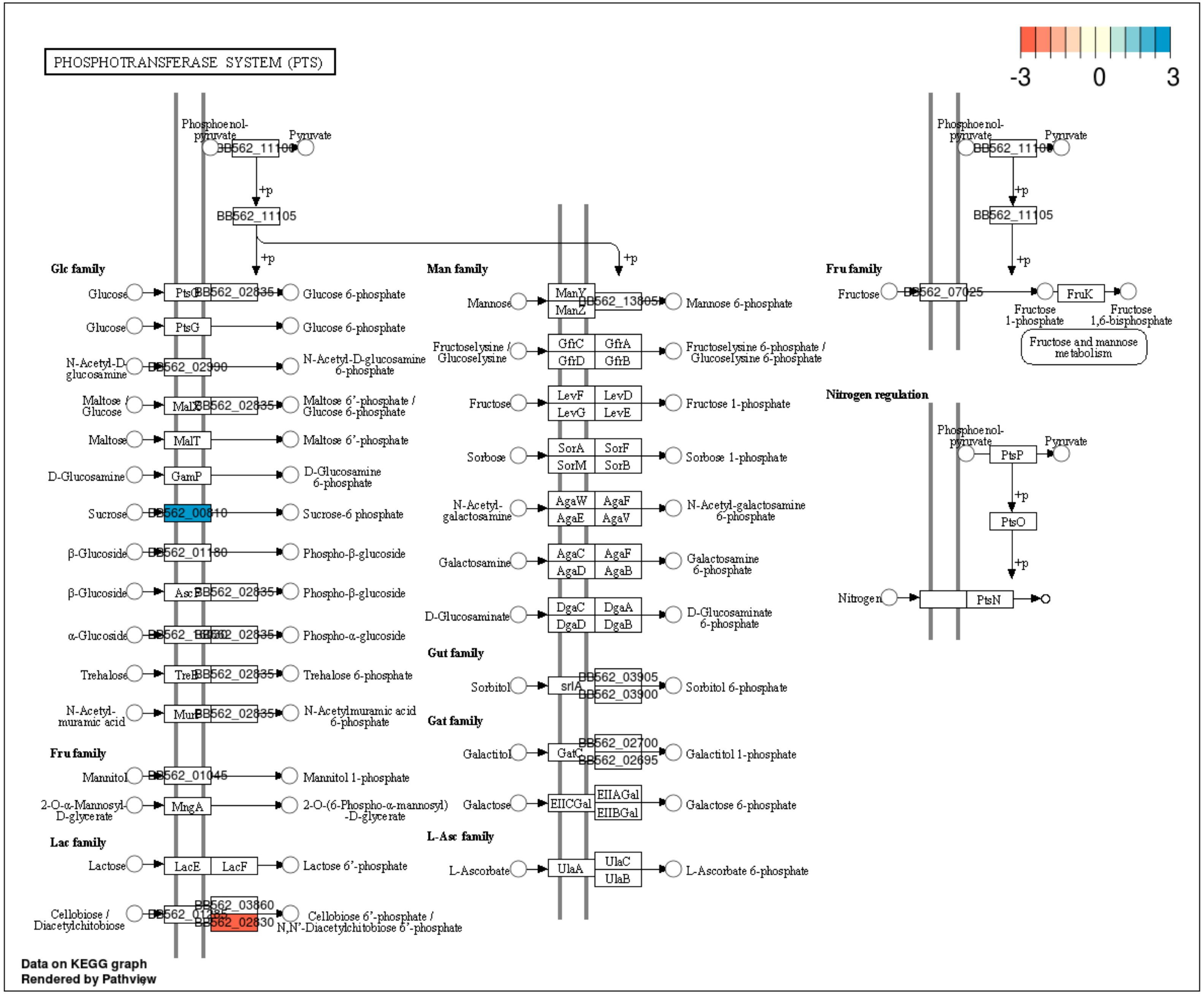

PHOSPHOTRANSFERASE SYSTEM (PTS)
-3
0
3
Glc family
Man family
Fru family
Nitrogen regulation
Gut family
Gat family
Fru family
Lac family
L-Asc family
Phosphoenol-pyruvate
Pyruvate
BB562_11100
BB562_11105
Glucose
Glucose 6-phosphate
PtsG
BB562_02835
N-Acetyl-D-glucosamine
BB562_02990
N-Acetyl-D-glucosamine 6-phosphate
Maltose / Glucose
MalX
Maltose 6'-phosphate / Glucose 6-phosphate
Maltose
MalT
Maltose 6'-phosphate
D-Glucosamine
GamP
D-Glucosamine 6-phosphate
Sucrose
BB562_00810
Sucrose-6 phosphate
β-Glucoside
BB562_01180
Phospho-β-glucoside
AscF
α-Glucoside
Phospho-α-glucoside
Trehalose
TreB
Trehalose 6-phosphate
N-Acetyl-muramic acid
MurP
N-Acetylmuramic acid 6-phosphate
Mannitol
BB562_01045
Mannitol 1-phosphate
2-O-α-Mannosyl-D-glycerate
MngA
2-O-(6-Phospho-α-mannosyl)-D-glycerate
Lactose
LacE
LacF
Lactose 6'-phosphate
Cellobiose / Diacetylchitobiose
BB562_03860
BB562_02830
Cellobiose 6'-phosphate / N,N'-Diacetylchitobiose 6'-phosphate
Mannose
ManY
ManZ
BB562_13805
Mannose 6-phosphate
Fructoselysine / Glucoselysine
GfrC
GfrD
GfrA
GfrB
Fructoselysine 6-phosphate / Glucoselysine 6-phosphate
Fructose
LevF
LevG
LevD
LevE
Fructose 1-phosphate
Sorbose
SorA
SorM
SorF
SorB
Sorbose 1-phosphate
N-Acetyl-galactosamine
AgaW
AgaE
AgaF
AgaV
N-Acetyl-galactosamine 6-phosphate
Galactosamine
AgaC
AgaD
AgaB
Galactosamine 6-phosphate
D-Glucosaminate
DgaC
DgaD
DgaA
DgaB
D-Glucosaminate 6-phosphate
Sorbitol
srlA
BB562_03905
BB562_03900
Sorbitol 6-phosphate
Galactitol
GatC
BB562_02700
BB562_02695
Galactitol 1-phosphate
Galactose
EIICGal
EIIAGal
EIIBGal
Galactose 6-phosphate
L-Ascorbate
UlaA
UlaC
UlaB
L-Ascorbate 6-phosphate
BB562_07025
FruK
Fructose 1-phosphate
Fructose 1,6-bisphosphate
Fructose and mannose metabolism
PtsP
PtsO
Nitrogen
PtsN
+p
Data on KEGG graph
Rendered by Pathview

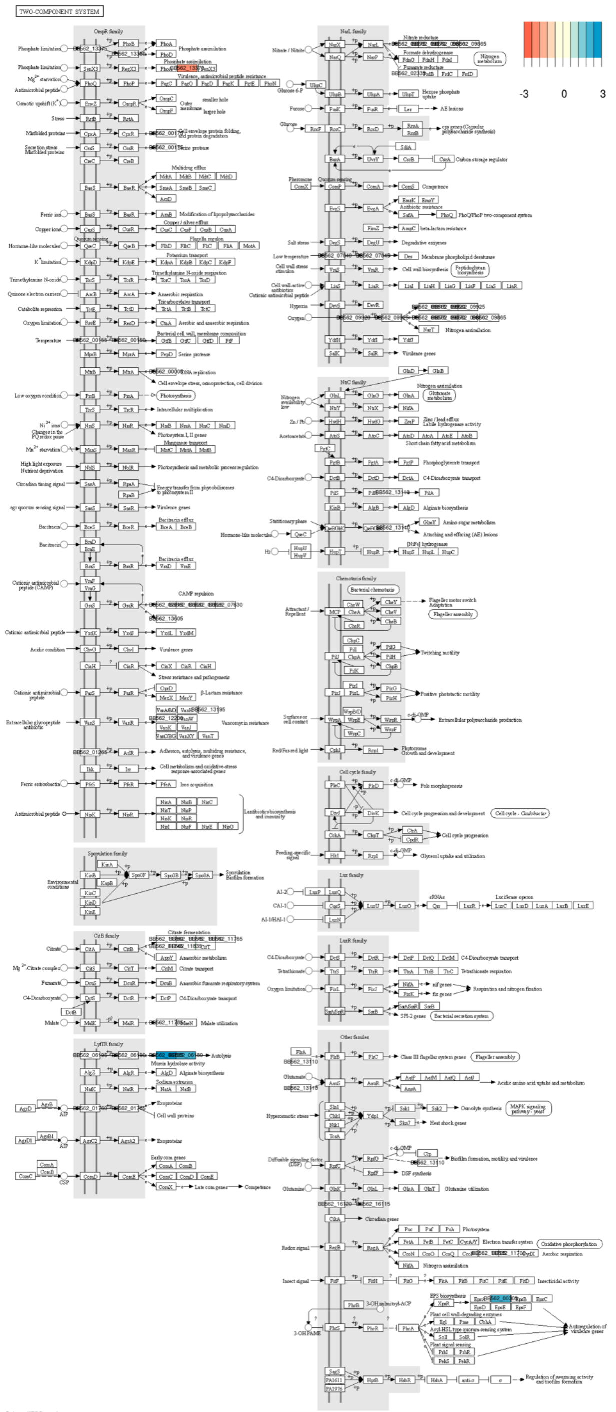

Data on KEGG graph
Rendered by Pathview

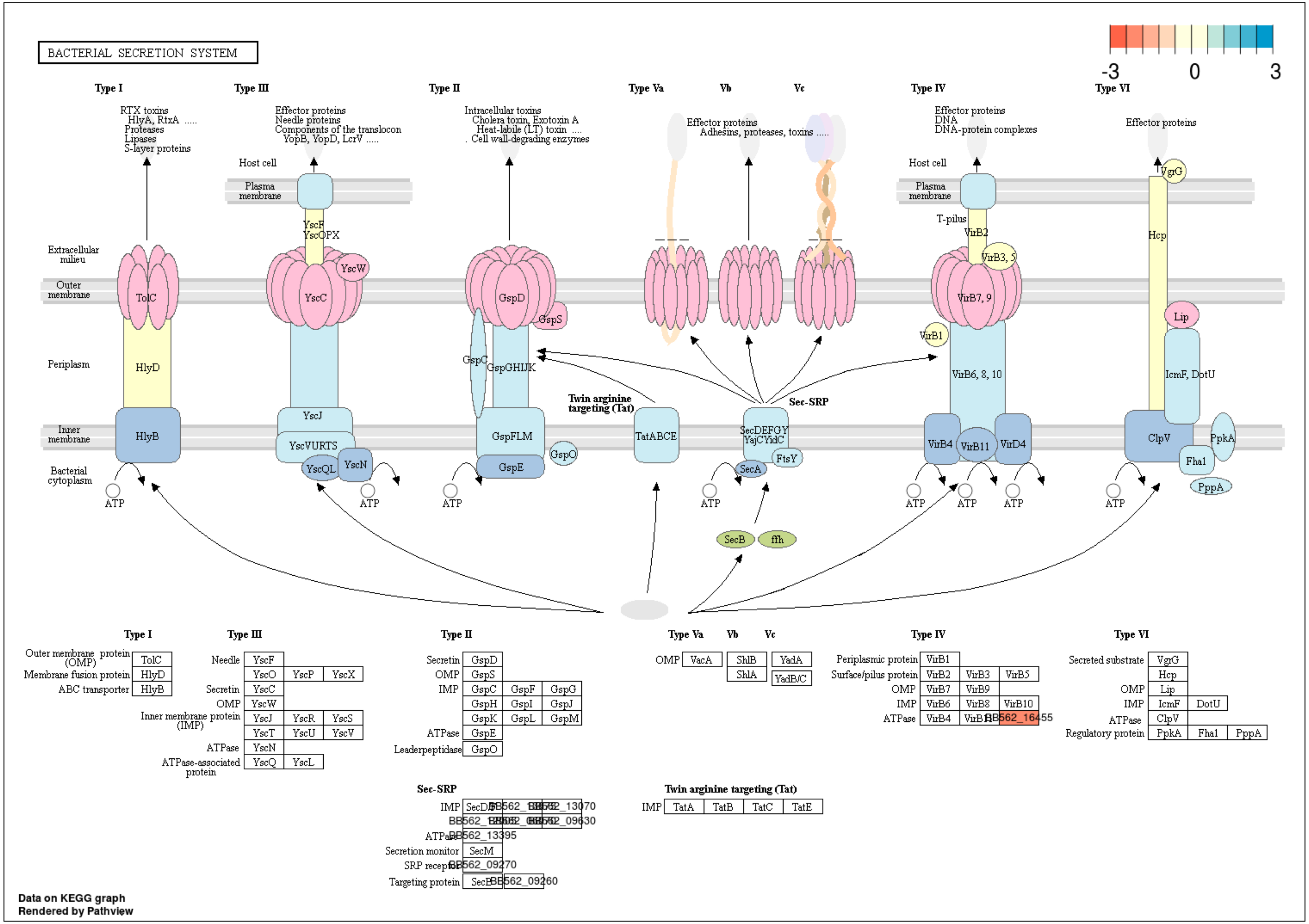
BACTERIAL SECRETION SYSTEM
-3
0
3
Type I
Type III
Type II
Type Va
Vb
Vc
Type IV
Type VI
RTX toxins
HlyA, RtxA .....
Proteases
Lipases
S-layer proteins
Effector proteins
Needle proteins
Components of the translocon
YopB, YopD, LcrV .....
Intracellular toxins
Cholera toxin, Exotoxin A
Heat-labile (LT) toxin .....
Cell wall-degrading enzymes
Effector proteins
Adhesins, proteases, toxins .....
Effector proteins
DNA
DNA-protein complexes
Effector proteins
Host cell
Plasma membrane
Extracellular milieu
Outer membrane
Periplasm
Inner membrane
Bacterial cytoplasm
TolC
HlyD
HlyB
YscF
YscOPX
YscW
YscC
YscJ
YscVURTS
YscQL
YscN
GspD
GspS
GspC
GspGHIJK
GspFLM
GspE
GspO
Twin arginine targeting (Tat)
TatABCE
Sec-SRP
SecDEFGY
YajC YidC
FtsY
SecA
SecB
ffh
T-pilus
VirB2
VirB3, 5
VirB7, 9
VirB1
VirB6, 8, 10
VirB4
VirB11
VirD4
VgrG
Hcp
Lip
IcmF, DotU
ClpV
PpkA
Fha1
PppA
ATP
Type I
Outer membrane protein (OMP)
TolC
Membrane fusion protein
HlyD
ABC transporter
HlyB
Type III
Needle
YscF
YscO
YscP
YscX
Secretin
YscC
OMP
YscW
Inner membrane protein (IMP)
YscJ
YscR
YscS
YscT
YscU
YscV
ATPase
YscN
ATPase-associated protein
YscQ
YscL
Type II
Secretin
GspD
OMP
GspS
IMP
GspC
GspF
GspG
GspH
GspI
GspJ
GspK
GspL
GspM
ATPase
GspE
Leaderpeptidase
GspO
Type Va
Vb
Vc
OMP
VacA
ShlB
ShlA
YadA
YadB/C
Type IV
Periplasmic protein
VirB1
Surface/pilus protein
VirB2
VirB3
VirB5
OMP
VirB7
VirB9
IMP
VirB6
VirB8
VirB10
ATPase
VirB4
Type VI
Secreted substrate
VgrG
Hcp
OMP
Lip
IMP
IcmF
DotU
ATPase
ClpV
Regulatory protein
PpkA
Fha1
PppA
Sec-SRP
IMP
Secretion monitor
SecM
SRP receptor
Targeting protein
Twin arginine targeting (Tat)
IMP
TatA
TatB
TatC
TatE
Data on KEGG graph
Rendered by Pathview

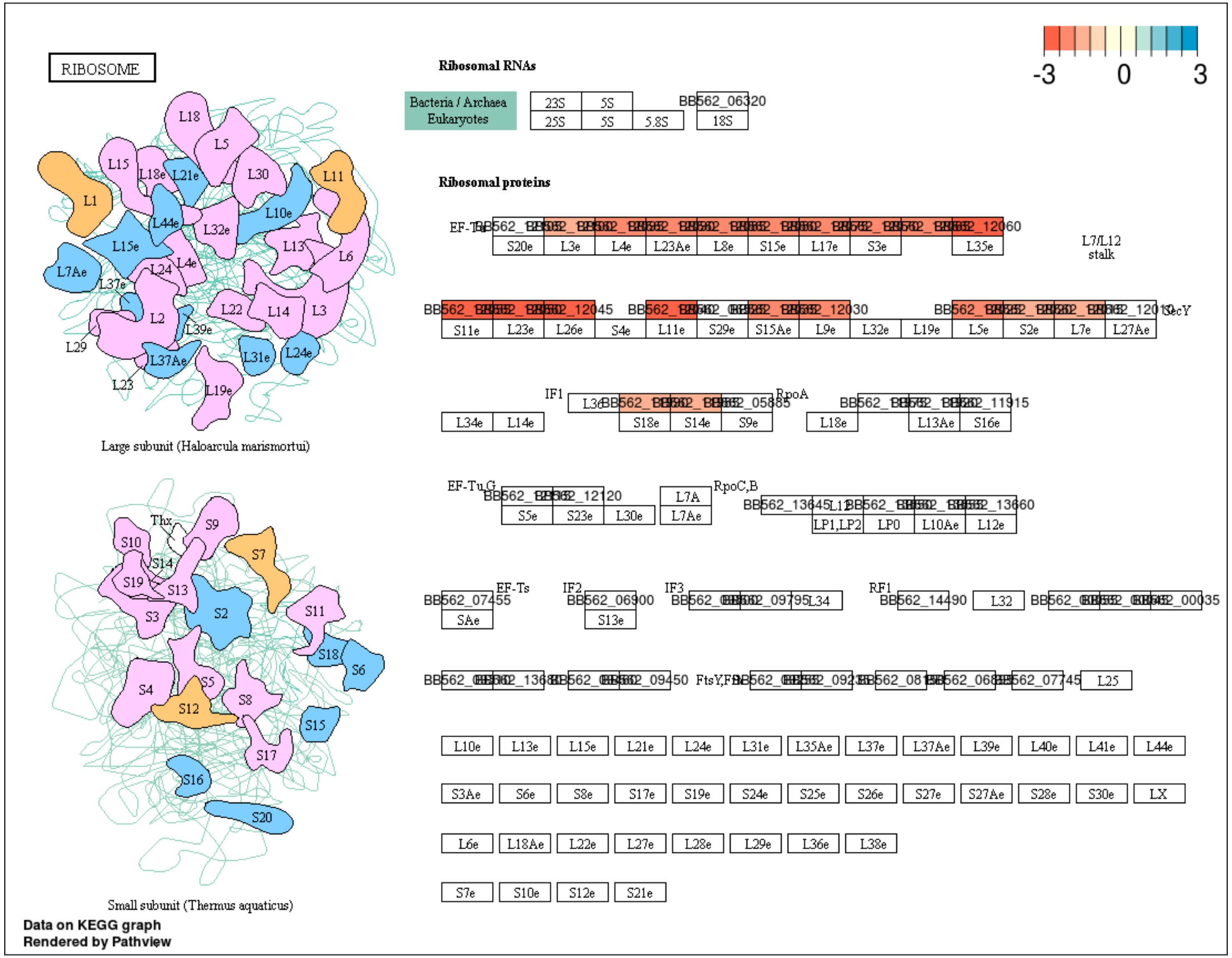
RIBOSOME
-3
0
3
Ribosomal RNAs
Bacteria / Archaea
Eukaryotes
23S
5S
25S
5S
5.8S
18S
Ribosomal proteins
L7/L12
stalk
S20e
L3e
L4e
L23Ae
L8e
S15e
L17e
S3e
L35e
S11e
L23e
L26e
S4e
L11e
S29e
S15Ae
L9e
L32e
L19e
L5e
S2e
L7e
L27Ae
SecY
IF1
RpoA
L34e
L14e
S18e
S14e
S9e
L18e
L13Ae
S16e
EF-Tu,G
RpoC,B
S5e
S23e
L30e
L7A
L7Ae
LP1,LP2
LP0
L10Ae
L12e
EF-Ts
IF2
IF3
RF1
SAe
S13e
L34
L32
FtsY,FfH
L25
L10e
L13e
L15e
L21e
L24e
L31e
L35Ae
L37e
L37Ae
L39e
L40e
L41e
L44e
S3Ae
S6e
S8e
S17e
S19e
S24e
S25e
S26e
S27e
S27Ae
S28e
S30e
LX
L6e
L18Ae
L22e
L27e
L28e
L29e
L36e
L38e
S7e
S10e
S12e
S21e
Large subunit (Haloarcula marismortui)
Small subunit (Thermus aquaticus)
Data on KEGG graph
Rendered by Pathview

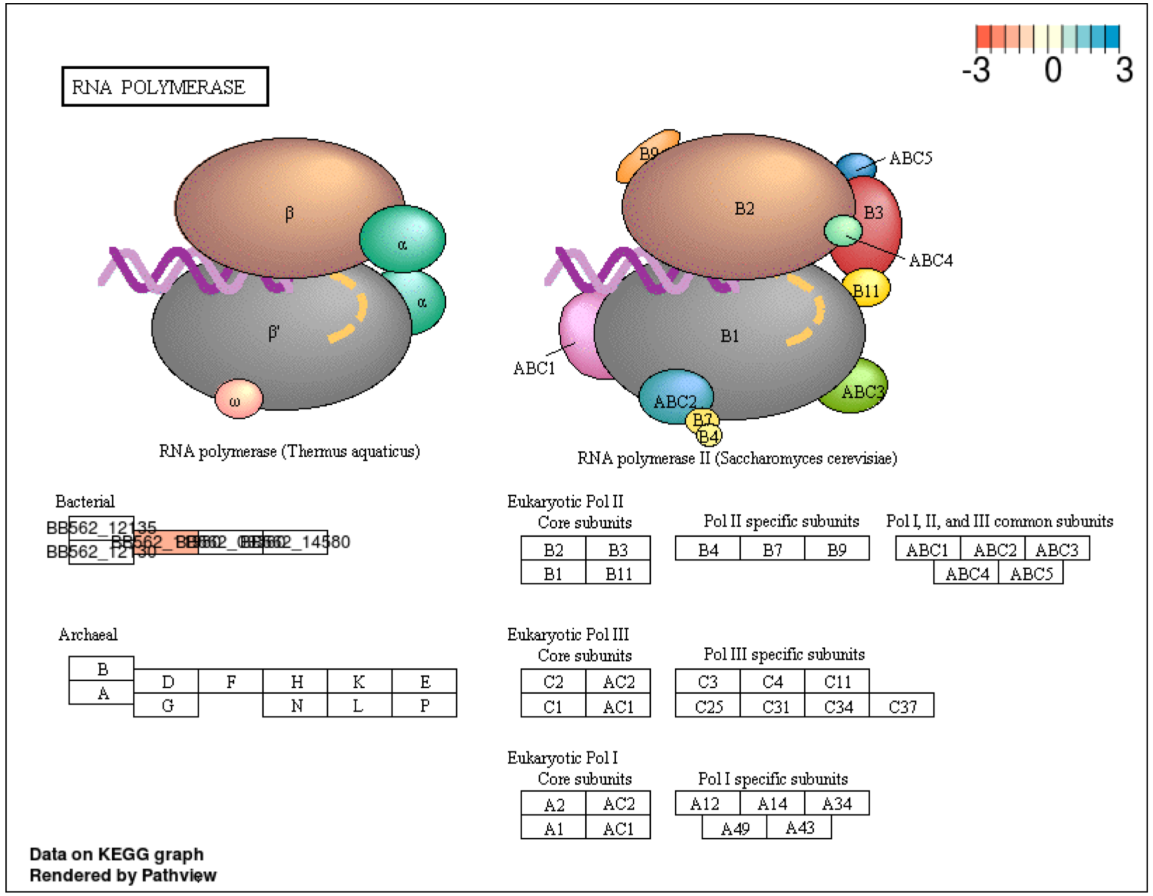
RNA POLYMERASE
-3 0 3
β
α
α
β'
ω
B9
B2
ABC5
B3
ABC4
B11
B1
ABC1
ABC2
ABC3
B7
B4
RNA polymerase (Thermus aquaticus)
RNA polymerase II (Saccharomyces cerevisiae)
Bacterial
BB562_12135
BB562_12130
BB562_14580
Eukaryotic Pol II
Core subunits
B2 B3
B1 B11
Pol II specific subunits
B4 B7 B9
Pol I, II, and III common subunits
ABC1 ABC2 ABC3
ABC4 ABC5
Archaeal
B
A
D F H K E
G N L P
Eukaryotic Pol III
Core subunits
C2 AC2
C1 AC1
Pol III specific subunits
C3 C4 C11
C25 C31 C34 C37
Eukaryotic Pol I
Core subunits
A2 AC2
A1 AC1
Pol I specific subunits
A12 A14 A34
A49 A43
Data on KEGG graph
Rendered by Pathview

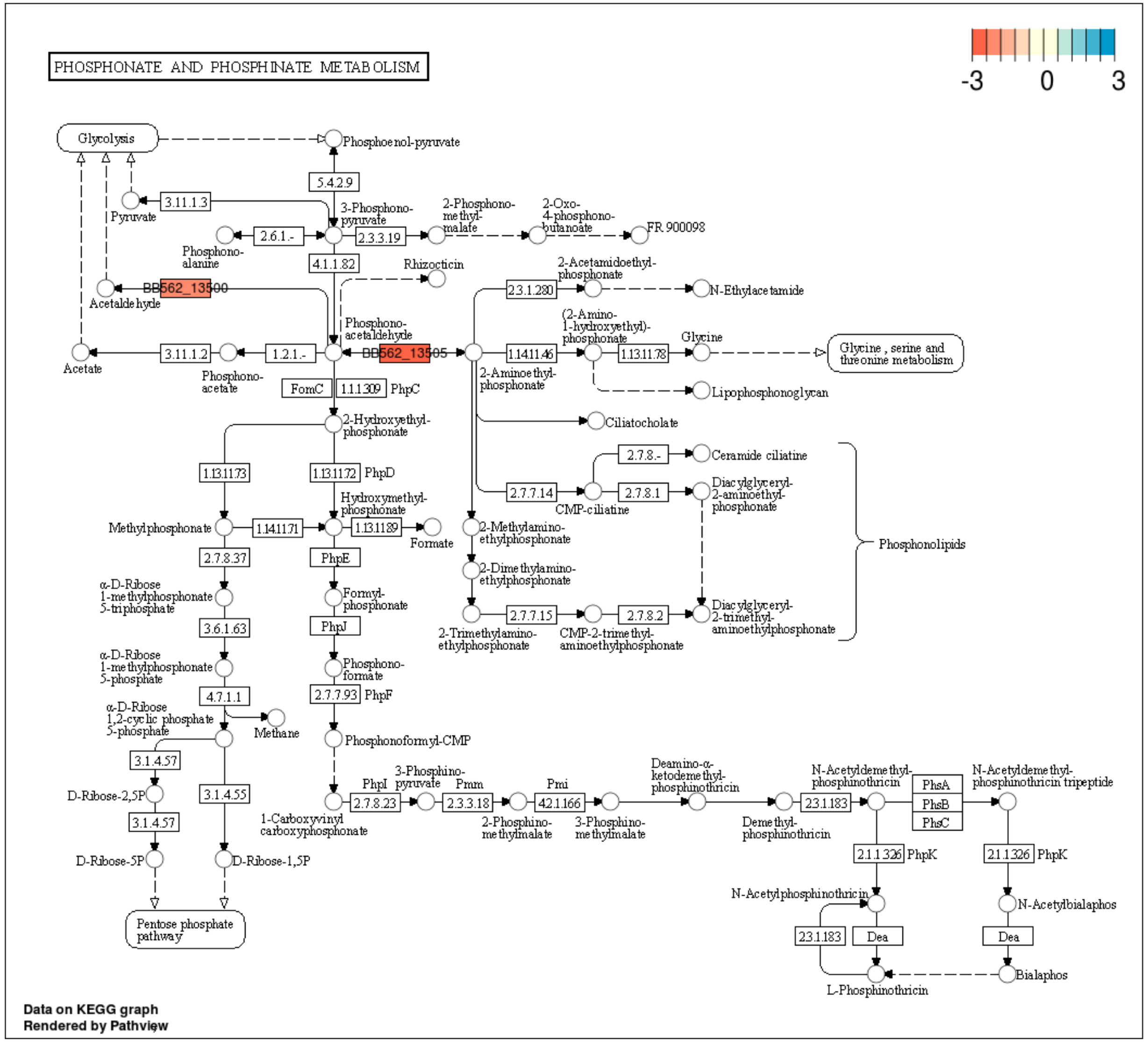
PHOSPHONATE AND PHOSPHINATE METABOLISM
-3 0 3
Glycolysis
Phosphoenol-pyruvate
5.4.2.9
3.11.1.3
Pyruvate
3-Phosphono-pyruvate
2.6.1.-
2.3.3.19
2-Phosphono-methyl-malate
2-Oxo-4-phosphono-butanoate
FR 900098
Phosphono-alanine
4.1.1.82
Rhizocticin
2-Acetamidoethyl-phosphonate
2.3.1.280
N-Ethylacetamide
BB562_13500
Acetaldehyde
Phosphono-acetaldehyde
(2-Amino-1-hydroxyethyl)-phosphonate
Glycine
Glycine, serine and threonine metabolism
Acetate
3.11.1.2
Phosphono-acetate
1.2.1.-
BB562_13505
1.14.11.46
1.13.11.78
2-Aminoethyl-phosphonate
Lipophosphonoglycan
FomC
1.1.1.309
PhpC
2-Hydroxyethyl-phosphonate
Ciliatocholate
2.7.8.-
Ceramide ciliatine
1.13.11.73
1.13.11.72
PhpD
Hydroxymethyl-phosphonate
2.7.7.14
CMP-ciliatine
2.7.8.1
Diacylglyceryl-2-aminoethyl-phosphonate
Phospholipids
Methylphosphonate
1.14.11.71
1.13.11.89
Formate
2-Methylamino-ethylphosphonate
2.7.8.37
PhpE
2-Dimethylamino-ethylphosphonate
α-D-Ribose 1-methylphosphonate 5-triphosphate
Formyl-phosphonate
2.7.7.15
2.7.8.2
Diacylglyceryl-2-trimethyl-aminoethylphosphonate
2-Trimethylamino-ethylphosphonate
CMP-2-trimethyl-aminoethylphosphonate
3.6.1.63
PhpJ
α-D-Ribose 1-methylphosphonate 5-phosphate
Phosphono-formate
4.7.1.1
2.7.7.93
PhpF
α-D-Ribose 1,2-cyclic phosphate 5-phosphate
Methane
Phosphonoformyl-CMP
3.1.4.57
3.1.4.55
D-Ribose-2,5P
3.1.4.57
D-Ribose-5P
D-Ribose-1,5P
Pentose phosphate pathway
1-Carboxyvinyl carboxyphosphonate
PhpI
2.7.8.23
3-Phosphino-pyruvate
Ppm
2.3.3.18
2-Phosphino-methylmalate
Pmi
4.2.1.166
3-Phosphino-methylmalate
Deamino-α-ketodemethyl-phosphinothricin
N-Acetyldemethyl-phosphinothricin
2.3.1.183
Demethyl-phosphinothricin
PhsA
PhsB
PhsC
N-Acetyldemethyl-phosphinothricin tripeptide
2.1.1.326
PhpK
2.1.1.326
PhpK
N-Acetylphosphinothricin
N-Acetylbialaphos
2.3.1.183
Dea
Dea
L-Phosphinothricin
Bialaphos
Data on KEGG graph
Rendered by Pathview

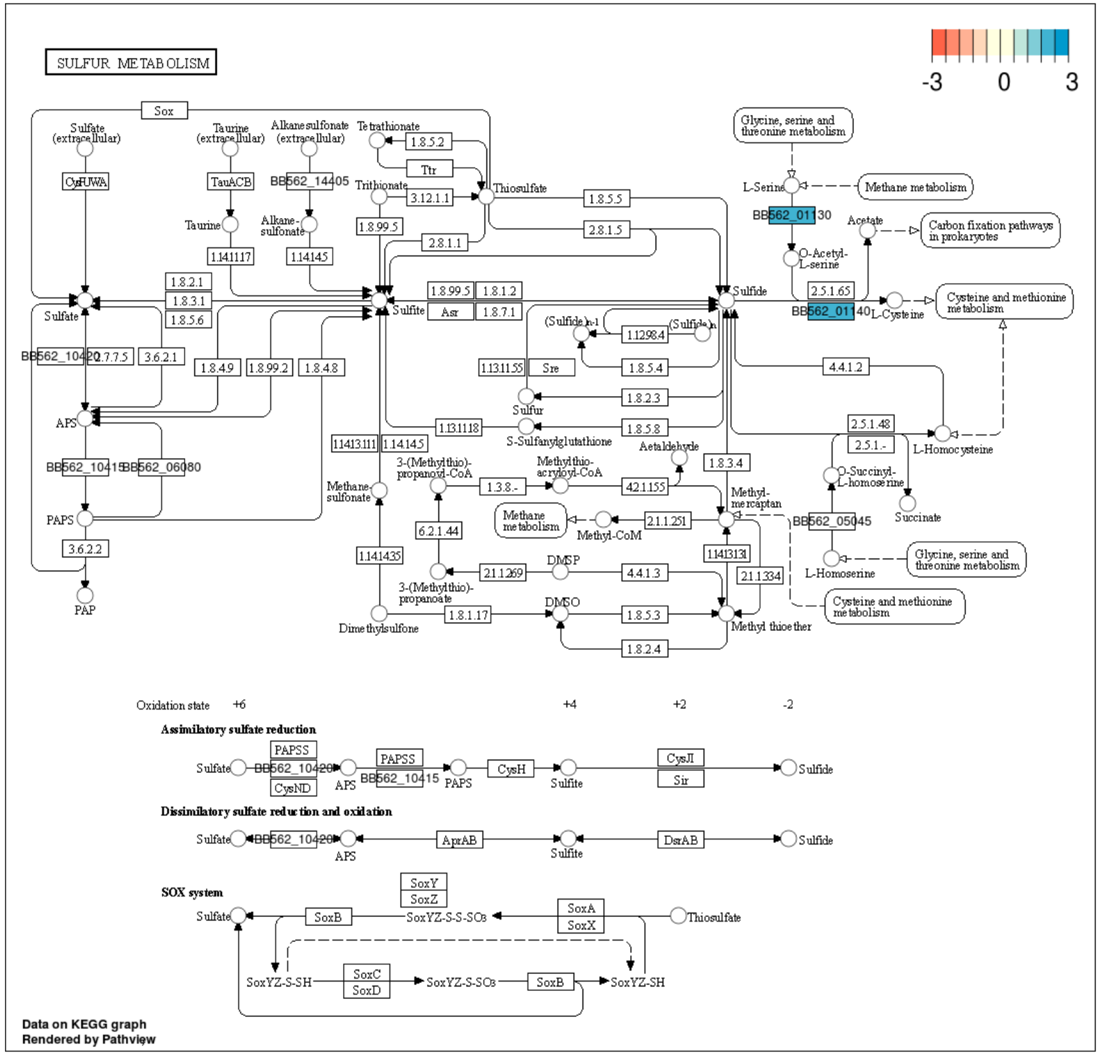
SULFUR METABOLISM
-3
0
3
Sox
Sulfate (extracellular)
Taurine (extracellular)
Alkanesulfonate (extracellular)
Tetrathionate
CysPUWA
TauACB
BB562_14405
Trithionate
Thiosulfate
Taurine
Alkane-sulfonate
Sulfate
Sulfite
Sulfide
APS
PAPS
PAP
BB562_10415
BB562_06080
BB562_10420
Sulfur
S-Sulfanylglutathione
Methanesulfonate
3-(Methylthio)-propanoyl-CoA
Methylthio-acryloyl-CoA
Acetaldehyde
Methane metabolism
Methyl-CoM
Methylmercaptan
DMSP
DMSO
3-(Methylthio)-propanoate
Dimethylsulfone
Methyl thioether
Glycine, serine and threonine metabolism
L-Serine
Methane metabolism
BB562_01130
Acetate
Carbon fixation pathways in prokaryotes
O-Acetyl-L-serine
BB562_01140
L-Cysteine
Cysteine and methionine metabolism
L-Homocysteine
O-Succinyl-L-homoserine
Succinate
BB562_05045
L-Homoserine
Glycine, serine and threonine metabolism
Cysteine and methionine metabolism
Oxidation state +6 +4 +2 -2
Assimilatory sulfate reduction
Sulfate
PAPSS
CysND
APS
PAPS
CysH
Sulfite
CysJI
Sir
Sulfide
Dissimilatory sulfate reduction and oxidation
AprAB
DsrAB
SOX system
SoxY
SoxZ
SoxYZ-S-S-SO3
SoxA
SoxX
Thiosulfate
SoxB
SoxYZ-S-SH
SoxC
SoxD
SoxYZ-S-SO3
SoxYZ-SH
Data on KEGG graph
Rendered by Pathview

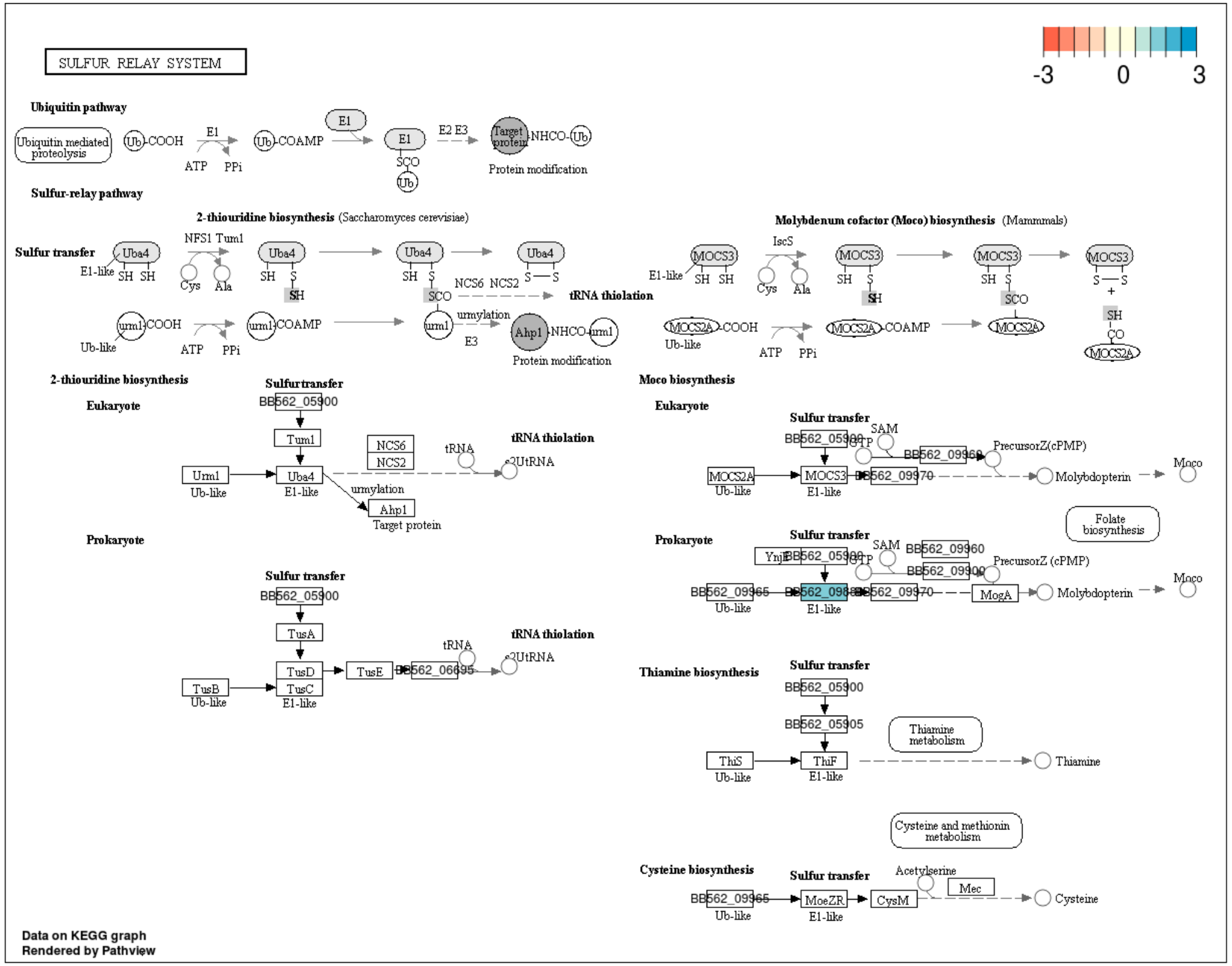
SULFUR RELAY SYSTEM
-3
0
3
Ubiquitin pathway
Ubiquitin mediated proteolysis
Ub-COOH
E1
ATP
PPi
Ub-COAMP
E2 E3
Target protein
NHCO-Ub
Protein modification
Sulfur-relay pathway
2-thiouridine biosynthesis (Saccharomyces cerevisiae)
Molybdenum cofactor (Moco) biosynthesis (Mammals)
Sulfur transfer
Uba4
E1-like
NFS1 Tum1
Cys
Ala
NCS6 NCS2
tRNA thiolation
urm1-COOH
Ub-like
urm1-COAMP
urmylation
E3
Ahp1
Protein modification
MOCS3
IscS
MOCS2A
COOH
COAMP
2-thiouridine biosynthesis
Eukaryote
Prokaryote
Sulfur transfer
BB562_05900
Tum1
Urm1
Uba4
NCS6
NCS2
tRNA
s2UtRNA
Ahp1
Target protein
TusA
TusB
TusD
TusC
TusE
BB562_06695
Moco biosynthesis
SAM
GTP
BB562_09960
PrecursorZ (cPMP)
MOCS2A
MOCS3
BB562_09970
Molybdopterin
Moco
Folate biosynthesis
YnjE
BB562_09965
MogA
Thiamine biosynthesis
BB562_05905
Thiamine metabolism
ThiS
ThiF
Thiamine
Cysteine and methionine metabolism
Cysteine biosynthesis
Acetylserine
Mec
MoeZR
CysM
Cysteine
Data on KEGG graph
Rendered by Pathview

Figure S3

Legend:

Figure S3. Overview of the KEGG metabolic pathways regulated in olive-adapted (TO) versus almond-adapted (TA) *L. pentosus* AP2-16. Metabolic pathways images were generated with the pathview v1.24.0 (Weijun L., 2013) R package and nodes were colored by adding log2FC of its genes for each comparison. The color of DEGs refers to the significance level of differential expressed genes.
